# Speciation, Structural Refinement, and Distribution of Ti Sites in Titanium Silicalite‐1 From ^47/49^Ti NMR Crystallography at 28.2 Tesla

**DOI:** 10.1002/anie.202524232

**Published:** 2026-02-24

**Authors:** Christoph J. Kaul, Jonas Koppe, Lukas Lätsch, Michael Wörle, Sadig Aghazada, Jacob B. Holmes, Christina Wartmann, Mingji Zheng, Albrecht Berkessel, Trees De Baerdemaeker, Andrei‐Nicolae Parvulescu, Karsten Seidel, J. Henrique Teles, Alexander V. Yakimov, Christophe Copéret

**Affiliations:** ^1^ Department of Chemistry and Applied Biosciences ETH Zurich Zurich Switzerland; ^2^ Centre De RMN à Très Hauts Champs de Lyon CNRS/Ecole Normale Supérieure De Lyon/Université Claude Bernard Lyon 1 Villeurbanne France; ^3^ Institut Des Sciences et Ingénierie Chimiques École Polytechnique Fédérale de Lausanne (EPFL) Lausanne Switzerland; ^4^ Department of Chemistry and Biochemistry Organic Chemistry University of Cologne Cologne Germany; ^5^ BASF SE Group Research Ludwigshafen Germany; ^6^ BASF SE Monomers Division Ludwigshafen Germany

**Keywords:** ^47/49^Ti NMR Spectroscopy, DFT Computation, NMR Crystallography, TS‐1, T‐sites

## Abstract

Titanium silicalite‐1 (TS‐1) is industrially used for selective oxidation processes. Despite being discovered over 40 years ago, TS‐1 is seeing an increase in use in the chemical industry. The catalyst structure, especially the Ti speciation and local environment in the zeolitic framework, as well as the nuclearity of the Ti sites in the pristine zeolite material, are still debated. Here, we address this issue by applying high field (28.2 T) ^47/49^Ti NMR and ^17^O NMR spectroscopy for an array of TS‐1 catalysts. For extra‐framework‐free TS‐1 catalysts, the Ti sites are associated with a distinct NMR signature—δ_iso_ (^49^Ti) = −900 ppm; C_Q,0_(^49^Ti) = 7.2 MHz—which can be translated into structural parameters and a dominant first coordination environment according to an extended Czjzek model. With the implementation of a ^47/49^Ti NMR crystallography protocol, benchmarked on molecular models, this signature was assigned to the presence of mononuclear Ti framework sites in pristine TS‐1 structures. The observed distribution of the NMR parameters presumably originates from variations of mononuclear Ti framework sites in the first and second coordination sphere, likely due to the occupancy of several T‐sites.

## Graphical abstract

Titanium silicalite‐1 (TS‐1), used in industrial selective oxidation processes, depends on specific, yet still unresolved Ti sites, dispersed within the framework of an MFI‐zeolite. Applying high field (28.2 T) ^47/49^Ti NMR and ^17^O NMR spectroscopy for an array of TS‐1 catalysts enables the development of an NMR crystallography protocol and the determination of the local environment of Ti sites and their distribution.

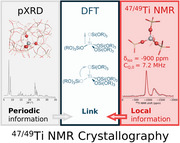

## 1 Introduction

**SCHEME 1:**
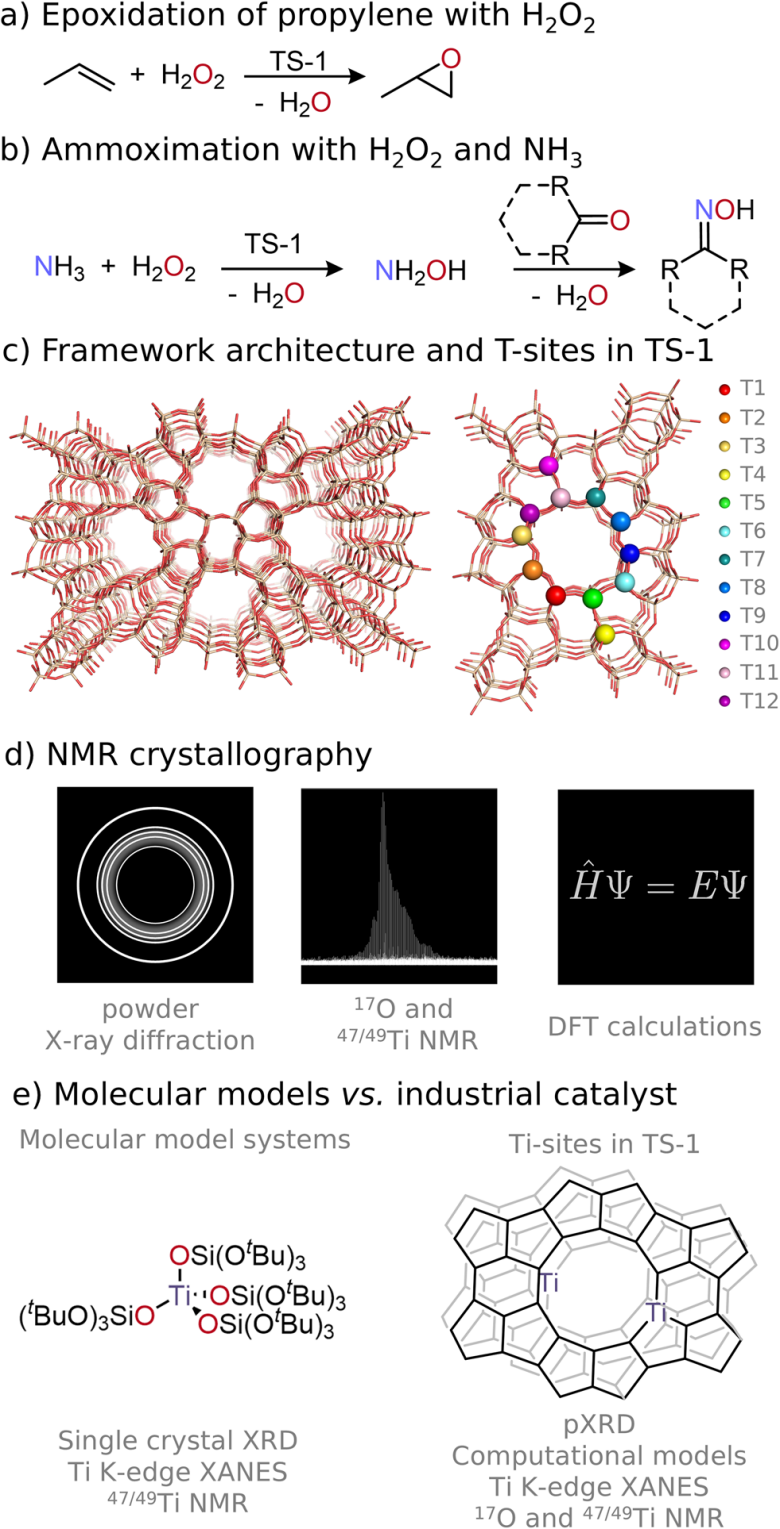
(a, b) Industrial application of TS‐1. (c) MFI framework architecture and the 12‐symmetry (MFI) independent T‐sites. (d) NMR crystallography consists of refining the pXRD structure with NMR spectroscopy (in this case ^17^O and ^47/49^Ti NMR) and DFT calculations. (e) Relating structural and spectroscopic signatures of a library of molecular models to TS‐1.

Titanium silicalite‐1 [1] (TS‐1, also known as Ti‐MFI) belongs to the large class of metal‐containing zeolites that have found industrial applications in selective oxidation reactions [1, 2, 3, 4]. Notably, TS‐1 is at the core of the environmentally friendly multimillion‐ton production of propylene oxide using hydrogen peroxide as the primary oxidant (Hydrogen Peroxide to Propylene Oxide—HPPO—process), yielding water as the sole co‐product [5, 6]. Besides propylene oxide production, the ammoximation of ketones employing TS‐1 has also been gaining importance (Scheme 1a,b) [7, 8, 9]. Since the discovery of TS‐1 ca. 40 years ago [1], the structure of the Ti sites, incorporated into the silicious zeolite matrix (Si_96−x_Ti_x_O_192_, up to *x*≈2.5) [10], has sparked interest and debates. Although a variety of characterization techniques (powder x‐ray diffraction (pXRD) [10, 11], x‐ray absorption spectroscopy (XAS) [12, 13, 14], infrared and Raman spectroscopy [15, 16], diffuse reflectance ultraviolet‐visible spectroscopy [17], and most recently solid‐state magic‐angle spinning (MAS) nuclear magnetic resonance (NMR) [11, 18, 19, 20] spectroscopy) are consistent with the isomorphous substitution of a small fraction of Si(IV) by Ti(IV) sites in the MFI [21] zeolite framework (up to ca. 2 wt%), the presence of framework‐associated defect sites is proposed to enhance catalytic activity [22]. Besides the question of Ti speciation in TS‐1, understanding the exact Ti coordination environment (local geometry, distortion, distribution of Ti sites) [3], is certainly key to establishing molecular‐level structure‐activity relationships. However, the debate has been mostly centered on the Ti location within the framework, that is, which specific T sites they occupy across the 12‐symmetry independent T‐sites (denoted T1‐T12, see Scheme 1c), because of the challenge to interrogate these structural features. Notably, even though zeolites are crystalline in nature, characterization via advanced diffraction methods, that is synchrotron radiation pXRD [23] and neutron powder diffraction (NPD) [24, 25, 26], has been challenging due to the low concentration of Ti (1–2 Ti wt%), the insufficient contrast between Ti and Si sites, the occurrence of vacancies within the framework, and the dependence of the results on the refinement strategy. Anomalous x‐ray powder diffraction at the Ti K‐edge enhances sensitivity toward the Ti location by changing its scattering factor when irradiated near‐resonance and has most recently been employed to locate preferential T‐site positions for Ti in TS‐1 [27]. However, this method exhibits similar limitations in terms of a reliable assignment of specific T‐sites due to strong dependence on the applied refinement method. In addition to diffraction methods, extended x‐ray absorption fine structure (EXAFS) has been evaluated toward finding preferred T‐sites for Ti in TS‐1 [28], but the intrinsically averaged information coming from XAS does not allow the differentiation between structurally similar tetrahedral sites via this technique. Considering the lack of experimental methods to clearly pinpoint specific Ti location, computational studies have also been employed [28, 29, 30, 31, 32, 33]. However, similar to experimental approaches, computations are not unambiguous and are limited by the lack of benchmarking.

Thus, there is a need to explore alternative element‐specific non‐averaging characterization method(s) augmented by computational approaches to determine the local structure of Ti sites in the zeolite framework as a first step toward a more rational catalyst design. Originally developed in the study of molecular organic [34] and inorganic solids [35], *NMR crystallography* [35, 36, 37, 38, 39, 40, 41, 42] has recently attracted growing interest in structural biology [40, 41, 43]. This approach, which combines x‐ray crystallography, NMR spectroscopy, and density functional theory (DFT) modelling (Scheme 1d), offers a powerful alternative in cases where single‐crystal XRD data are ambiguous or insufficient for structure refinement. Similarly, the analysis of zeolites and layered silicates also suffers from the lack of suitable single crystals, and hence, low‐resolution pXRD analysis is mostly employed for the structure determination. In fact, ^29^Si NMR in combination with pXRD measurements has been employed for the NMR‐based structure‐determination approach of zeolites [44] and molecularly ordered but noncrystalline silicate frameworks [45], highlighting the feasibility of NMR crystallography to assist refining zeolite structures.

We reason that the study of the structure of Ti sites in TS‐1 is possible by combining insights gained from pXRD measurements, the ^17^O NMR signatures of oxygen atoms in the vicinity of Ti centers, and ^47/49^Ti NMR spectroscopy augmented with DFT calculations [46]. Whereas ^17^O NMR is more widely applied in the zeolite community [47, 48, 49, 50], ^47/49^Ti NMR spectroscopy [51, 52] has emerged as a powerful method to characterize Ti for molecular [53] and high weight loading bulk materials [54, 55, 56] and only recently has been extended to heterogenous catalysts, including Ziegler–Natta pre‐catalysts [57, 58], USY [59] and TS‐1 [18, 60] zeolites. Even though ^47/49^Ti NMR spectroscopy is hampered by low sensitivity, due to the low gyromagnetic ratios, low natural abundances (NAs) and the quadrupolar nature of the two NMR active nuclei [γ(^47^Ti) = −1.5105×10^7^ rad s^−1^ T^−1^, NA(^47^Ti) = 7.44%, *I*(^47^Ti) = 5/2, *Q*(^47^Ti) = 30.2 fm^2^) and γ(^49^Ti) = −1.51095×10^7^ rad s^−1^ T^−1^, NA(^49^Ti) = 5.41%, *I*(^49^Ti) = 7/2, *Q*(^49^Ti) = 24.7 fm^2^] [61], it exhibits large responses to small structural variations. Hence, it is poised to extract detailed information regarding Ti speciation and local geometry, making it, in principle, suitable to discriminate between different Ti environments and even T‐site positions. For the Ti sites in TS‐1, only qualitative analysis of the ^47/49^Ti NMR spectroscopic signatures has been carried out, because benchmarking of the computational protocols has so far been hampered by the limited amount of molecularly defined references measured [18]. Similarly, it has not been possible to address the question of mononuclear versus dinuclear sites, which have been previously proposed to form in TS‐1 upon contact with H_2_O_2_ [62], since the ^47/49^Ti NMR signatures alone are not sensitive enough to distinguish them [18].

However, recent developments in commercial high‐field NMR spectrometers (>28 T) enhance the sensitivity toward species exhibiting large quadrupole couplings and increase the resolution for Ti species with small to moderate quadrupole couplings such that the NMR signatures for the ^47^Ti and ^49^Ti isotope can be resolved, yielding a higher precision for the extracted ^47/49^Ti NMR parameters (the central transition linewidth of half‐integer quadrupole nuclei, due to the anisotropic second order boarding, is inversely proportional to the external magnetic field strength, hence ultra‐high field increases both sensitivity and resolution) [63]. The overall increase in sensitivity leads to reduced measurement times, enabling comparisons between samples. Note that the accessibility to ultra‐high field instruments still remains limited; yet the NMR community has invested large collaborative efforts to make these instruments and advanced methods more accessible.

Here, we exploit state‐of the art ^17^O and high‐field (up to 28.2 T) ^47/49^Ti NMR spectroscopy under static and MAS conditions, involving double frequency sweeps (DFS) [64, 65] and Carr–Purcell–Meiboom–Gill (CPMG) [66, 67, 68] detection for signal enhancement; for static conditions, an ultra‐wideline methodology [69, 70, 71] employing wideband, uniform rate, smooth truncation (WURST) [72] pulses is employed to reliably extract the full spectroscopic signatures and associated NMR parameters, which are the chemical shift (δ), the span (Ω) and the asymmetry parameter (κ), as well as the quadrupole coupling constant (C_Q_) and the corresponding asymmetry parameter (η_Q_). Notably, the C_Q_ is related to the spherical symmetry in the ground state electron density around the metal center (high spherical symmetry corresponds to low C_Q_). Based on the NMR parameters, we postulate the presence of mainly mononuclear Ti sites in a dominant local Ti environment influenced by the distribution of C_Q_s. Applying this approach to a set of TS‐1 zeolites with different Ti loadings and synthesis procedures shows that the observed Ti environment is independent of metal loading across the examined samples. In addition, extra‐framework sites are visible in ^47/49^Ti NMR signatures when they are present, making this methodology powerful for the understanding of Ti speciation in TS‐1 and related materials. Finally, the information gained from the NMR spectroscopic signatures in combination with the results from pXRD measurements, Ti K‐edge x‐ray Absorption Near Edge Structure (XANES), and benchmarked computational modelling (Scheme 1e) enables the determination of the predominant Ti environment in TS‐1. Based on this analysis, we find that the dominant local Ti environment in dehydrated TS‐1 is best described by mononuclear framework Ti sites with varying first and second coordination spheres.

## 2 Results

We first focus on a prototypical—*classical*, close to the originally reported recipe [1] — TS‐1 catalyst TS‐1_1.5_ (1.5 Ti wt%) [62], containing minimal amounts of extra‐framework TiO_2_ according to UV–vis spectroscopy (see Figure S1; for synthesis details and catalytic evaluation see Supporting Information's Sections A and B, respectively). As a starting point, the powder x‐ray diffractogram of the dehydrated sample is recorded; it shows a typical MFI pattern with unit cell parameters *a* = 20.0834(6) Å, *b* = 19.9050(7) Å, *c* = 13.3847(5) Å and a cell volume of 5350.7(3) Å^3^ in the orthorhombic space group *Pnma* (see Supporting Information Section G for experimental details).

### 2.1 ^17^O NMR Signatures, Exchangeable Oxygen, and Implications for the Ti Environment

**FIGURE 1:**
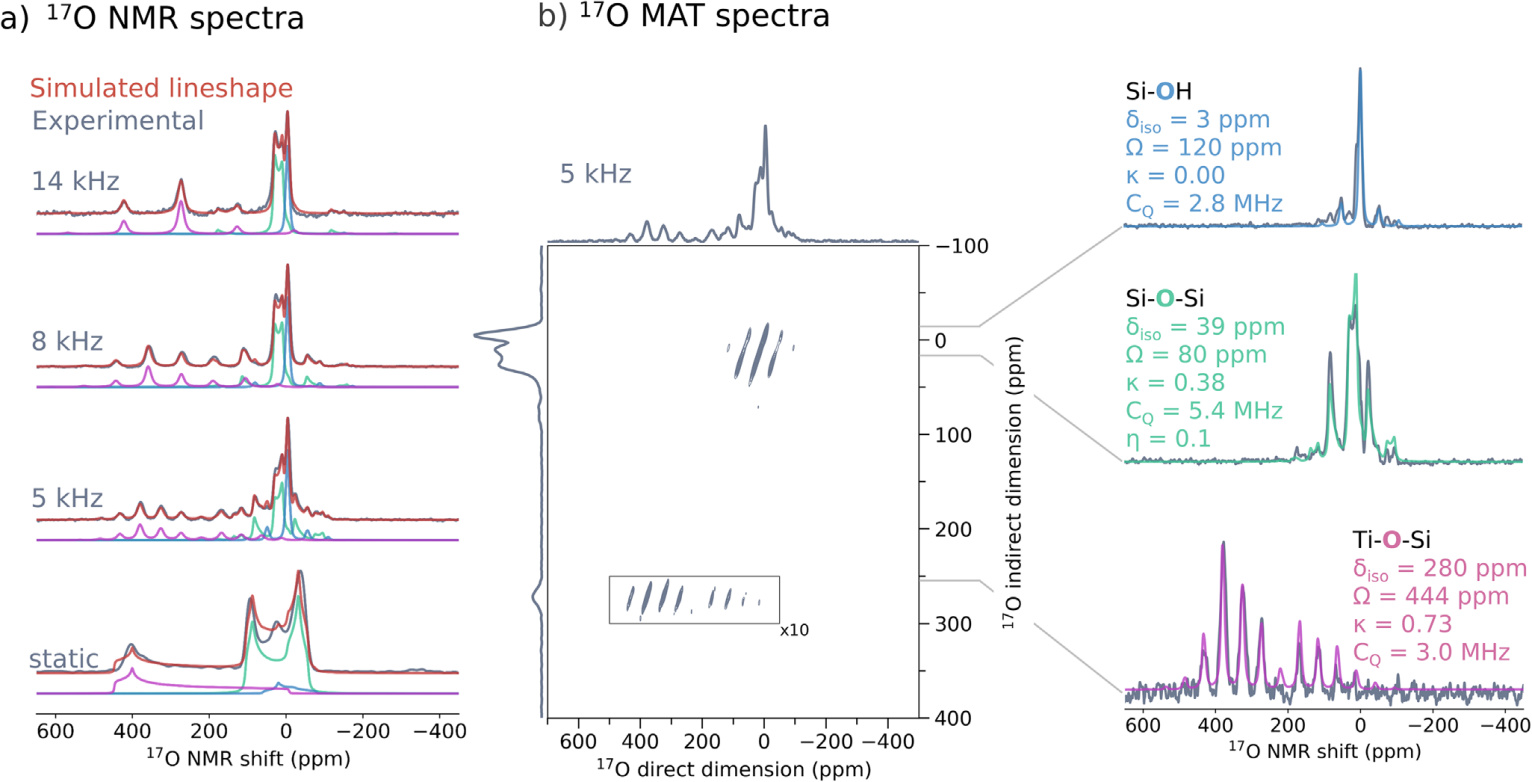
^17^O NMR measurements of TS‐1 (298 K, 16.4 T, referenced to H_2_O at room temperature (0 ppm)). (a) ^17^O NMR spectra at different spinning speeds and deconvoluted into ^17^O signatures from Si─OH (*blue*), Si─O─Si (*green*), and Ti─O─Si (*violet*). (b) ^17^O MAT spectrum (5 kHz) after application of a 2D filter function [78] and the extracted slices for the three species Si─OH (*blue*), Si─O─Si (*green*), and Ti─O─Si (*violet)*.

In the next step, we set out to probe the Ti environment via the ^17^O NMR signatures of the oxygen atoms in the direct Ti vicinity. To circumvent the extremely low natural abundance of the NMR‐active ^17^O nuclei (NA = 0.038%), ^17^O isotope labelling is needed. Therefore, the sample is subjected to H_2_^17^O (90% enriched) treatment at room temperature for ca. 1 week, followed by a dehydration step (see Supporting Information Section H.3.1 for further details), in line with literature procedures [49]. The ^17^O labelling of the zeolite framework enables the measurement of the ^17^O NMR signatures at different spinning rates and to perform ^17^O magic‐angle‐turning (MAT) experiments [73]. In contrast to earlier reports [47], the combination of 1D MAS and 2D MAT experiments enables the reliable extraction of the specific spectroscopic features of the overlapping signals in the MAS dimension (Figure 1). The obtained spectra reveal three exchangeable O‐sites in TS‐1, which are attributed to ^17^O in Si─OH, Si─O─Si (see Figure S14 for ^17^O NMR signature of Silicalite‐1), and Ti─O─Si environments. This result is in line with previous assignments based on model systems and is further confirmed by our computational studies (see Supporting Information Section I.4.2) [74, 75, 76]. Notably, no signal associated with Ti─O─Ti is observed [77], consistent with the absence of a significant amount of dinuclear sites in the pristine sample (see Supporting Information Section I.4.2 for further discussions).

The extracted ^17^O NMR parameters for the three species are as follows for (δ_iso_/Ω/κ)/[C_Q_]:

- (3 ppm, 120 ppm, 0.00) / [2.8 MHz] for Si─OH,
- (39 ppm, 80 ppm, 0.38) / [5.4 MHz] for Si─O─Si,
- (280 ppm, 444 ppm, 0.73) / [3.0 MHz] for Ti─O─Si.

Notably, the integral ratio of Ti─O─Si/ Si─O─Si (see Table S21) is much greater than expected from a statistical distribution of ^17^O in the zeolite matrix. This points to the fact that the oxygen is favourably exchanged in the vicinity of Ti, suggesting distinct ^17^O insertion kinetics for Ti─O─Si and Si─O─Si, in line with previous reports [47, 48]. In fact, as previously proposed, the mechanism of water insertion into the Ti─O─Si bond most likely proceeds via a five‐coordinated Ti intermediate, which allows for explaining the favorable oxygen exchange in the direct vicinity of the Ti site [79]. Overall, although the ^17^O NMR signature of the bridging oxo sites in principle reflects the different Ti‐site connectivities, it gives only limited structural information regarding the Ti sites themselves (see Section I.4.3 for sensitivity discussion of ^17^O NMR parameters toward structural changes of Ti sites).

### 2.2 ^47/49^Ti NMR and XANES Signatures of Model TS‐1 Catalyst

**FIGURE 2:**
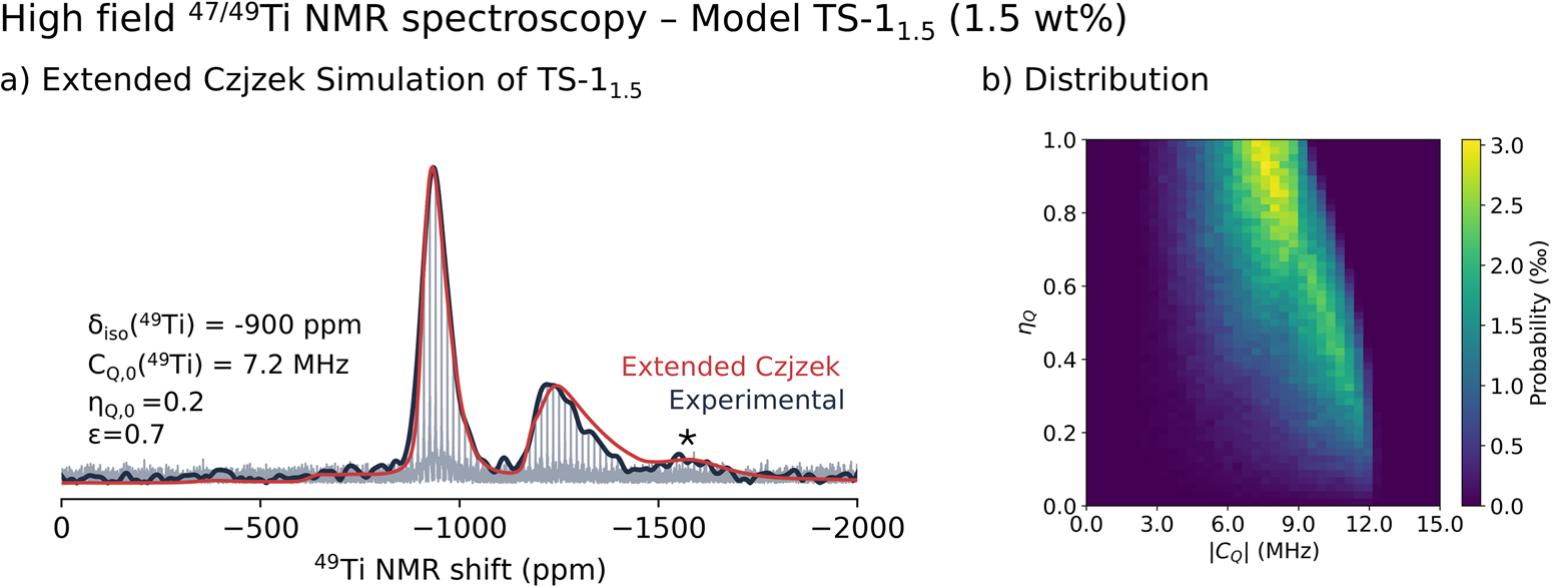
(a) ^47/49^Ti CPMG‐MAS NMR spectra of TS‐1_1.5_ recorded at 298 K, 20 kHz, and 28.2 T. The spikelet spectrum (*grey*) is obtained by Fourier transformation of the entire CPMG echo train, followed by a magnitude calculation. The echoes of the CPMG experiments were co‐added in the time domain, followed by a Fourier transformation of the resulting echo. Magnitude calculation yields the envelope spectrum (in *dark blue*; additional experimental details are provided in the Supporting Information). The extended Czjzek model simulation is shown above (in *red*). Spectra are referenced to SrTiO_3_ at room temperature (−843 ppm). Spinning sideband is marked with an asterisk. (b) The distribution of quadrupole coupling parameters used for the extended Czjzek model simulation.

As ^17^O NMR spectroscopy of the oxygen atoms in the vicinity of Ti allows only limited insights into the structure of Ti sites, we next focus on spectroscopic techniques that probe Ti directly, namely Ti K‐edge XANES and ^47/49^Ti NMR spectroscopies. The dehydrated TS‐1_1.5_ sample contains mostly tetrahedral Ti species according to the pre‐edge feature of Ti K‐edge XANES (see Figure S8). However, Ti K‐edge XANES is known to be rather insensitive to small structural changes of the tetrahedral environment (vide infra) [14]. Hence, we next investigate the ^47/49^Ti NMR spectroscopic signatures of Ti sites in the dehydrated TS‐1 via ^47/49^Ti CPMG‐MAS NMR spectroscopy (see Section H.2.1 and Figure S11 for experimental details and the static spectrum, respectively). Notably, both ^47^Ti and ^49^Ti NMR active isotopes are readily detected at 28.2 T (see Figure 2), with a spectral signature tailing toward higher field, reminiscent of a distribution of quadrupole coupling parameters [80]. Therefore, the ^47/49^Ti NMR signatures of the dehydrated TS‐1 are simulated with an extended Czjzek model [80, 81, 82] accounting for both the ^47^Ti and ^49^Ti isotopes and the presence of a dominant local structure (C_Q,0_ and η_Q,0_) and (random) disorder in the next coordination spheres, parameterized by Ɛ. Here, the absence of disorder is indicated by Ɛ = 0 (see Section H.5 for further details). This model allows us to confidently extract the NMR parameters from the dominant local Ti environment: δ_iso_ (^49^Ti) = −900 ppm and C_Q,0_(^49^Ti) = 7.2 MHz (see Figure 2a); along with a distribution of quadrupole coupling parameters, displaying a large spread of η_Q_ values (Ɛ = 0.7) (see Figure 2b). Note that these NMR signatures vanish upon hydration of the sample (vide infra for further comments), consistent with the reaction of the Ti sites with water giving penta‐coordinated species associated with very large C_Q_, hence currently not detectable by NMR at such low abundance, even at high‐fields [79].

### 2.3 Effect of Ti Wt% Loading on the Ti Environment

**FIGURE 3:**
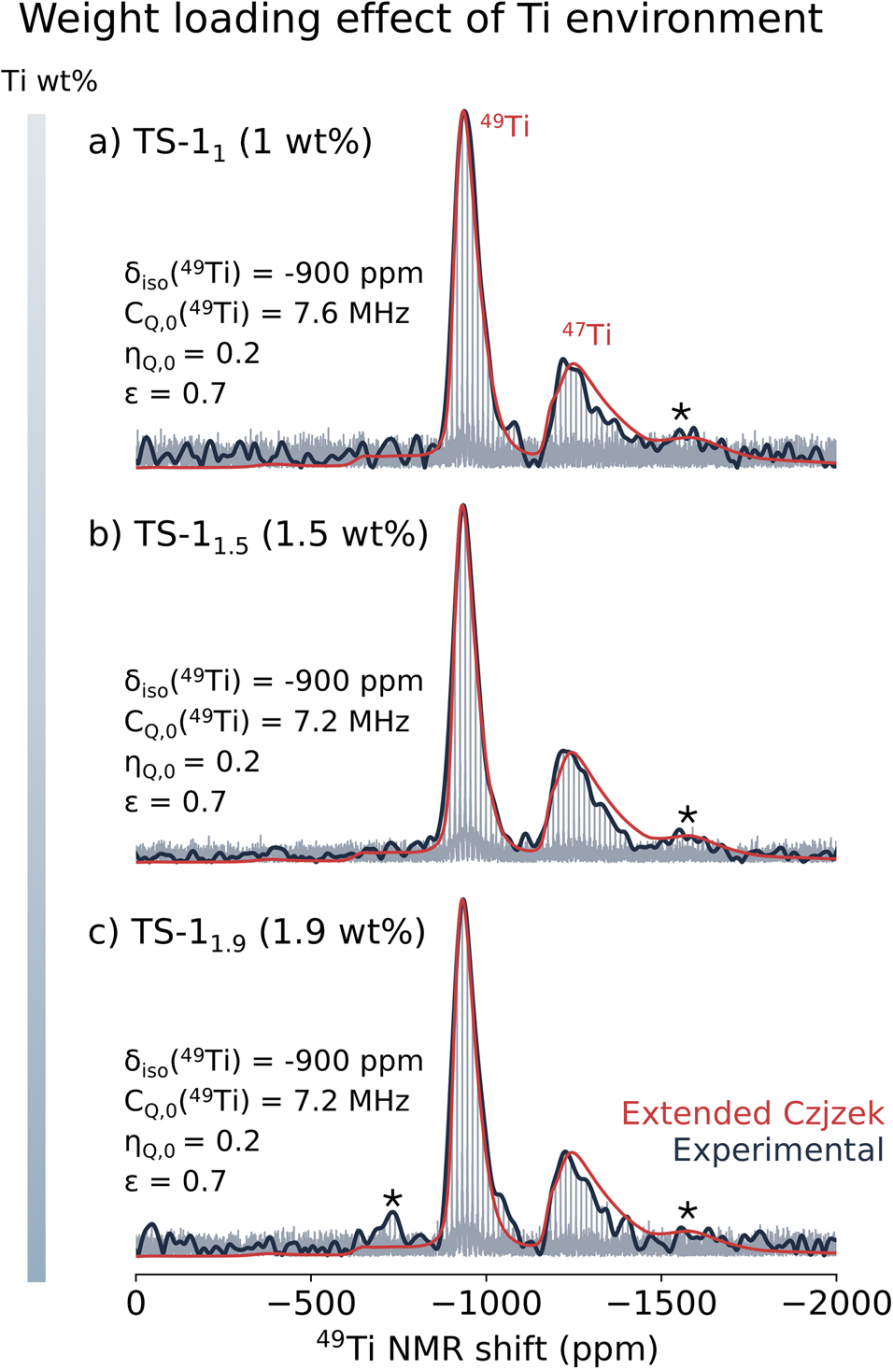
Investigation of possible effects on framework incorporation as a function of weight loading via ^47/49^Ti NMR. (a–c) TS‐1 samples with different weight loadings (TS‐1_1,_ TS‐1_1.5,_ TS‐1_1.9_). ^47/49^Ti CPMG‐MAS NMR spectra of the respective TS‐1 recorded at 298 K, 20 kHz, and 28.2 T (in *dark blue*, see caption of Figure 2 and Supporting Information for additional details). The extended Czjzek model simulations are shown in *red*. Spinning sidebands are marked with asterisks.

Next, we investigate the possible weight loading effect on the dominant Ti environment and its distribution, reflected in quadrupolar coupling parameters associated with it. Therefore, we explore additional TS‐1 samples, besides the previously investigated model TS‐1_1.5_, with different Ti weight loadings, containing minimal amounts of extra‐framework TiO_2_ (see Figure S1 for UV–vis spectra): a model TS‐1 catalyst TS‐1_1_ (1 Ti wt%) and a TS‐1 catalyst produced on an industrial scale, TS‐1_1.9_ (1.9 Ti wt%) [62]. The ^47/49^Ti MAS NMR spectra are depicted in Figure 3 along with their extended Czjzek model simulation. The distribution of ^47/49^Ti NMR parameters is basically identical, irrespective of the amount of Ti atoms incorporated into the framework matrix. This suggests that the dominant Ti environment does not change upon incorporation of additional Ti into the framework, which is consistent with similar catalyst performances (all three zeolites show high propylene epoxidation productivity, see Section B.3 for catalytic evaluations).

### 2.4 Extra‐Framework Free Versus Extra‐Framework Containing Zeolite

**FIGURE 4:**
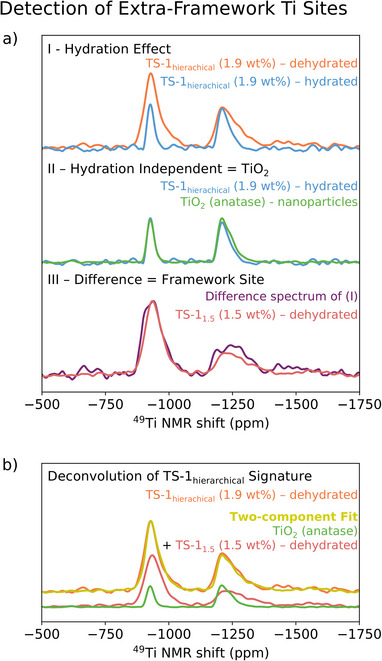
(a) (I) Effect of hydration on the signature of TS‐1_hierarchical_. (II) ^47/49^Ti NMR signature of hydrated TS‐1_hierarchical_ overlayed with the signature of TiO_2_ (anatase). (III) Difference spectrum of the normalized TS‐1_hierarchical_ hydrated/dehydrated spectra plotted with the spectrum of dehydrated TS‐1_1.5_. (b) Two‐component fit (framework Ti sites and extra‐framework TiO_2_) of the ^47/49^Ti NMR spectrum of dehydrated TS‐1_hierarchical_.

Besides these more *classical* TS‐1 catalysts, we also investigate a *hierarchical* TS‐1 (TS‐1_hierarchical_, 1.9 Ti wt%). While the synthesis of hierarchical structures aims at preserving or even enhancing the exceptionally high activity of TS‐1, it leads to (often unavoidable) formation of extra‐framework TiO_2_ [83, 84]. The investigated TS‐1_hierarchical_ indeed displays an exceptionally high activity toward the formation of propylene oxide (see section B.3 for catalytic performance data), while containing significant amounts of extra‐framework TiO_2_, as evidenced by Ti K‐edge XANES and UV–vis spectroscopy (see Figures S1 and S9). Notably, TS‐1_hierarchical_ displays ^47/49^Ti NMR signatures in both hydrated and non‐hydrated states (see Figure 4aI), in sharp contrast to the TS‐1 samples discussed before (free of extra‐framework TiO_2_), for which the ^47/49^Ti NMR signal vanishes upon hydration (see Figure S12 for spectra in the hydrated state). This suggests that the TS‐1_hierarchical_ catalyst contains some Ti sites whose NMR signatures are not affected by hydration, consistent with the presence of extra‐framework Ti (see Figure 4aI) as evidenced by Ti K‐edge XANES and UV–vis spectroscopy. Notably, the ^47/49^Ti NMR signatures of hydrated TS‐1_hierarchical_ and anatase (TiO_2_) nanoparticles show a striking resemblance (see Figure 4aII). This strongly indicates that the extra‐framework Ti species are anatase‐like rather than rutile‐like, considering that the latter would exhibit significantly broader ^47/49^Ti NMR signatures (see Figure S13) due to a low spherical symmetry of the Ti site [51]. The absence of rutile, which promotes H_2_O_2_ decomposition [48], is also consistent with the good catalyst performance observed for this sample.

Furthermore, the difference spectrum of the hydrated and dehydrated Ti spectra of TS‐1_hierarchical_ (normalized by the number of scans) shows strong resemblance with the ^47/49^Ti NMR signature of the dehydrated TS‐1_1.5_ free of extra‐framework TiO_2_ (see Figure 4aIII). In fact, the ^47/49^Ti NMR spectrum of dehydrated TS‐1_hierarchical_ can be decomposed via a two‐component fit (see Figure 4b) into the signatures of anatase TiO_2_ and TS‐1_1.5_ (Ti framework sites).

Using spin counting (assuming similar effective T2 relaxation constants for extra‐framework TiO_2_ structures and bulk anatase TiO_2_, see Section H.6 for further details), one can determine the amount of anatase‐like TiO_2_ which corresponds to ca. 0.8 Ti wt% of the TS‐1_hierarchical_ sample, in line with the pre‐edge feature analysis of the Ti K‐edge spectra, which suggests that ca. half of the Ti sites (equivalent to ca. 0.9 Ti wt%) of the sample are hexa‐coordinated Ti sites (see Section F.3).

In general, comparing the ^47/49^Ti NMR signatures of hydrated and dehydrated samples allows differentiation between framework and extra‐framework Ti sites in TS‐1, and provides insight into the polymorphism of the extra‐framework TiO_2_. In fact, we additionally studied a classical TS‐1 catalyst with a Ti weight loading of 3.5 wt%, exceeding the limit of full Ti framework incorporation [10]. This catalyst displays a ^47/49^Ti NMR signature also in the hydrated state, clearly pointing to the presence of extra‐framework TiO_2_ (see Section H.7 for a more detailed discussion). This methodology is therefore a complementary tool to existing techniques (that is, Ti K‐ and L‐edge XAS), allowing to also interrogate small structural variations in both tetrahedral and octahedral environments of Ti sites in TS‐1.

## 3 Discussion

### 3.1 Sensitivity of ^47/49^Ti NMR and XANES Signatures to Local Environment From a Molecular Library

In the next step, we set out to obtain molecular‐level information from the ^47/49^Ti NMR spectroscopic signatures of the dehydrated TS‐1_1.5_ catalyst, representative of the main Ti site in the studied TS‐1 catalysts. Hence, we study tailored molecular analogues in various coordination environments to better relate spectroscopic signatures to structural and electronic parameters. As molecular analogues, we have selected compounds closely related to framework Ti sites, namely [TiX(OTBOS)_3_], X = OTBOS = tris(*tert*‐butoxy)siloxy, (**1**) as well as related compounds with various X‐ligands (**2–4**, with X = O*^i^* Pr, NMe_2_, and Cl), to evaluate the effect of electronic asymmetry on ^47/49^Ti NMR parameters. This is noteworthy since these molecular models allow assessment of the response for ^47/49^Ti NMR signatures in comparison to Ti K‐edge XANES toward singular ligand exchange (as found for TiOH vs. fully framework incorporated Ti sites). We also include compounds with hexa‐coordinated Ti sites, closely related to alternative possible sites (framework‐associated Ti sites) or small clusters (**5, 6**).

**FIGURE 5:**
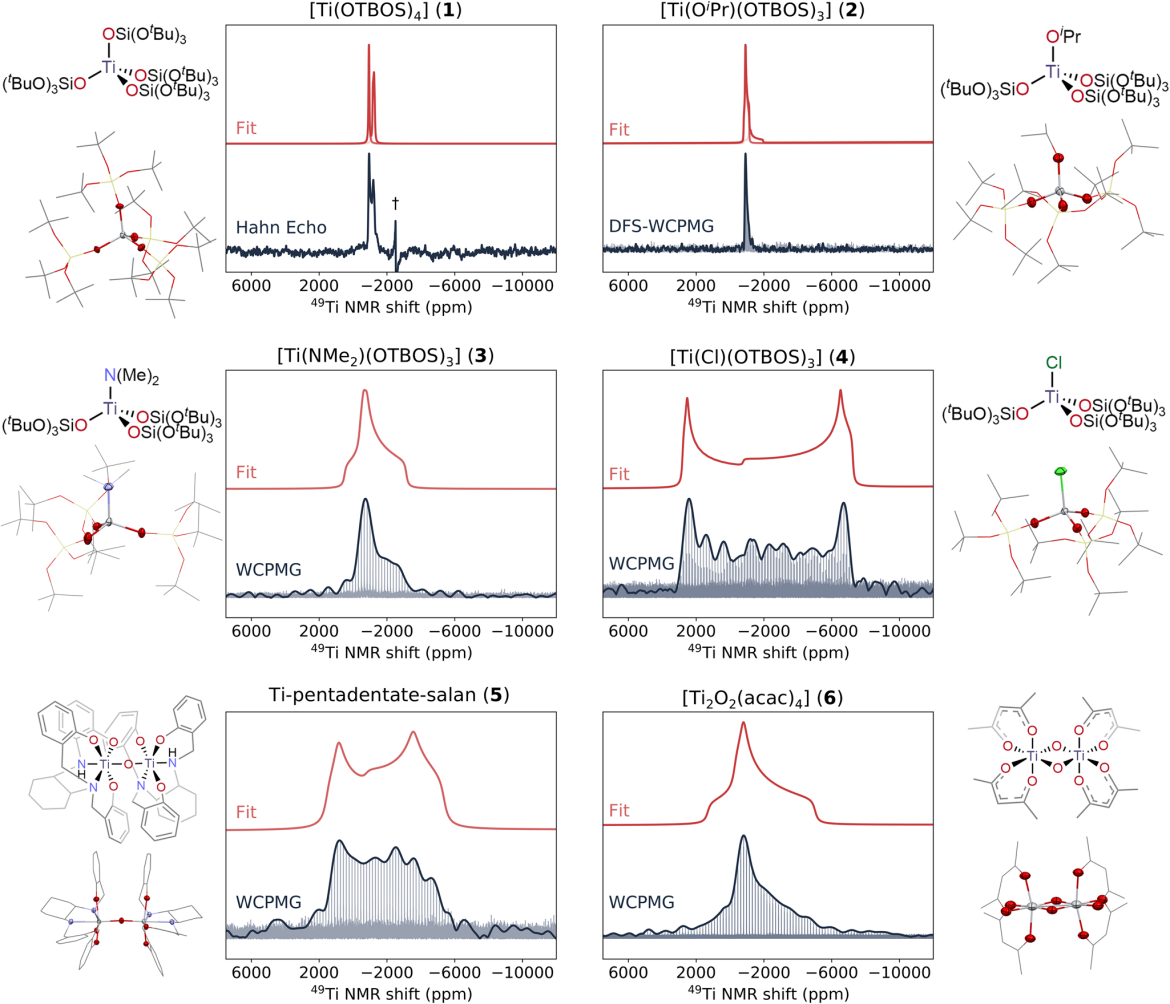
Low temperature ^47/49^Ti NMR signatures of the molecular library members: Tetrahedral complexes [TiX(OTBOS)_3_] (with X = OTBOS, O*^i^* Pr, NMe_2_, Cl, (**1–4**) Ti‐pentadentate‐salan (**5**) and [Ti_2_O_2_(acac)_4_] (**6**). Each panel contains the single crystal XRD structure and the static ^47/49^Ti NMR spectrum recorded at 100 K and 14.1 T (in *dark blue*, see caption of Figure 2 and Supporting Information for additional details), and the corresponding fit of the ^49^Ti NMR signature (*red*, for complexes **1** and **2**, both ^47^Ti and ^49^Ti are fitted). The † marks a measurement artifact.

Note that the four tetracoordinated Ti complexes (**1‐4**) show close to perfect tetrahedral symmetry according to single crystal x‐ray crystallography with all τ_4_^′^ [85] between 0.96 and 0.99 and a range of Ti‐X bond lengths: 1.782 Å for OTBOS [18], 1.748 Å for O*^i^* Pr [86], 1.875 Å for NMe_2_ [87] and 2.226 Å for Cl (see Table 1). Notably, while the Ti K‐edge XANES signatures are consistent with tetrahedral environments, only marginal differences are observed in the pre‐edge feature between the samples, despite the significant difference in the geometry and electronic structure (see Figure S6), showing the limited insights provided by Ti K‐edge XANES across this family of compounds. In sharp contrast, these tetrahedral complexes (**1–4**) display significantly different ^49^Ti NMR signatures (see Figures 5 and S26 for individually magnified spectra) and associated NMR parameters, in terms of both isotropic chemical shift (δ_iso_) and C_Q_ values. For instance, [Ti(OTBOS)_4_] and [Ti(O*^i^* Pr)(OTBOS)_3_], differing solely by one siloxy versus alkoxy ligand, exhibit similar yet distinct ^49^Ti isotropic chemical shifts δ_iso_ of −933 ppm and −914 ppm, with significant differences in their respective quadrupole coupling constant (C_Q_) 1.7 versus 4.0 MHz for [Ti(OTBOS)_4_] and [Ti(O*^i^* Pr)(OTBOS)_3_], respectively. This is in line with the decreased spherical symmetry around the metal center, consistent with previous reports [18]. Notably, all ^47/49^Ti NMR parameters for molecular complexes have been obtained with low temperature (100 K) measurements at 14.1 T, to be consistent with the single crystal XRD measurements. Not surprisingly, exchange of the OTBOS ligand by less electronegative X‐ligands (OR>Cl>NR_2_, see Table 1) [88] leads to significantly increased chemical shifts and larger quadrupole coupling constants (δ_iso_/C_Q_), (−776 ppm/28.7 MHz) and (−716 ppm/13.1 MHz) for [TiCl(OTBOS)_3_] and [Ti(NMe_2_)(OTBOS)_3_], respectively. The sensitivity of the NMR parameters (δ_iso_/C_Q_) toward the spherical symmetry of the Ti coordination environment confirms that ^47/49^Ti NMR spectroscopy is well‐suited to differentiate between ligand environments and local symmetry. This is of crucial importance for distinguishing subtle geometrical variations between framework T‐sites (imposed by the framework) as well as those associated with the presence of titanols, framework, and framework‐associated sites. Notably, while the chemical shift is driven by the electronegativity (EN) difference between Ti and X, the change in the quadrupole coupling constant is highly affected by the Ti‐X bond length as shown across this molecular series (see Table 1).

**TABLE 1:** Computed ^49^Ti NMR parameters (experimental given in parentheses) of molecular complexes [TiX(OTBOS)_3_] (with X = OTBOS (**1**), O*^i^* Pr (**2**), NMe2 (**3**), Cl (**4**), Ti‐pentadentate‐salan (**5**), and [Ti_2_O_2_(acac)_4_] (**6**) and framework sites of TS‐1 catalyst.

	1 X = OTBOS	2 X = O*^i^* Pr	3 X = NMe_2_	4 X = Cl	5	6	TS‐1 Framework site
δ_iso_ (ppm)	−918 (−933)	−929 (−914)	−657 (−716)	−765 (−776)	−844 (−840)	−768 (−798)	−822–−891 (−900)
C_Q_ (MHz)	3.3 (1.7)	3.6 (4.0)	12.6 (13.1)	26.3 (28.7)	23.0 (22.1)	20.2 (16.9)	6.7–11.1 (7.2)
ƞ_Q_	–	0.95 (0.90)	0.79 (0.92)	0.22 (0.05)	0.15 (0.25)	0.95 (1.00)	—
Pauling EN of X	3.44	3.44	3.04	3.16	—	—	—
d(Ti‐X) (Å)	1.821 (1.782)	1.784 (1.748)	1.893 (1.875)	2.255 (2.226)	—	—	—

Regarding the octahedral Ti dinuclear complexes studied in this work, their Ti K‐edge XANES features display a significant reduction in the pre‐edge feature height (forbidden dipole transition, no p/d mixing) with the appearance of additional new features in the pre‐edge region (additional low intensity quadruple transitions), as expected for an octahedral geometry [14]. Besides the pronounced change of the pre‐edge feature, the octahedral molecular Ti complexes are associated with large C_Q_ values, due to their distorted and/or heteroleptic (N, O) ligand spheres: Ti‐pentadentate‐salan: δ_iso_ = −840 ppm, C_Q_ = 22.1 MHz, η_Q_ = 0.25, [Ti_2_O_2_(acac)_4_]: δ_iso_ = −798 ppm, C_Q_ = 16.9 MHz, η_Q_ = 1.0. This further demonstrates the capability of ^47/49^Ti NMR to detect and differentiate between a wide range of species.

### 3.2 Sensitivity of ^47/49^Ti NMR Toward Local Environment

The ^47/49^Ti NMR signature of TS‐1_1.5_ is consistent with one dominant local Ti environment. The additional line broadening reflects a distribution of quadrupolar parameters and nearby environments (in the first and second coordination sphere, vide infra). Considering the significant effect of small changes in Ti‐X bond distances and bond angles on the ^47/49^Ti NMR signatures (chemical shift and C_Q_), they are expected to be sensitive to Ti speciation and location in framework T‐site positions. The sensitivity of NMR parameters and possible information contained in the NMR signatures motivates further analysis. Thus, we investigate the effects of: (i) Ti speciation, and (ii) distortion in the first and second coordination sphere (to quantitatively address the structural variations), with the ultimate goal to understand the observed Ti signatures, and address Ti speciation [or location in T‐site (s)] in TS‐1.

In order to better understand the origin of these NMR spectroscopic signatures and relate them to specific structural features, we employ DFT calculations, which are based on a two‐step approach: (i) periodic structure optimization [89, 90, 91, 92, 93] and (ii) NMR parameter calculations of single molecules for molecular references, or cluster models for zeolite structures [93, 94, 95, 96, 97, 98] (see Section I of the Supporting Information for more details). The computed δ_iso_ for [Ti(OTBOS)_4_], [TiCl(OTBOS)_3_], and [Ti(NMe_2_)(OTBOS)_3_] show the same chemical shift trend as experimentally observed (values given in parentheses): −918 (−933), −765 (−776) and −657 (−716) ppm, respectively, highlighting the validity of the computational approach (see Table 1). Similarly, the trend in quadrupole coupling constants matches well between computed and experimental values across the series of tetracoordinated complexes: C_Q_ = 3.3 (1.7), 3.6 (4.0), 12.6 (13.1) MHz, and 26.3 (28.7) MHz for [Ti(OTBOS)_4_], [Ti(O*^i^* Pr)(OTBOS)_3_], [Ti(NMe_2_)(OTBOS)_3_], and [TiCl(OTBOS)_3_], respectively. Similar good agreements are also found for octahedral dinuclear Ti compounds, with one (**5**) or two μ_2_‐oxo (**6**) bridges: δ_iso calc_(δ_iso exp_)/C_Q,calc_(C_Q,exp_)/η_Q, calc_(η_Q, exp_) = −844 (−840) ppm/23.0 (22.1) MHz/0.15 (0.25) and −768 (−798) ppm/20.2 (16.9) MHz/0.95 (1.00) for **5** and **6**, respectively (see Supporting Information for further details and discussion, in particular Figure S26).

Furthermore, the computed and experimental data open the possibility to determine the global uncertainties for the predicted NMR parameters a priori (root mean square error (RMSE) for both isotropic chemical shift δ_iso RMSE_(^49^Ti) = 29 ppm and quadrupole coupling constant C_Q RMSE_(^49^Ti) = 1.9 MHz, see Supporting Information Section I.5.1), thereby enabling *^47/49^Ti NMR crystallography* based on the reduced *χ* ^2^ statistic to be implemented for Ti‐zeotypes. We note that so far, this approach of structure selection has been mainly employed for biological systems (that is, active site characterization of proteins) [40, 41, 43]. Commonly, 10 or more chemical shifts (most common nuclei are ^1^H, ^13^C, ^15^N, ^17^O) are required to fully distinguish between structures [40, 41, 43]. Herein, we will focus on the ^47/49^Ti NMR parameters, since they are the most sensitive toward small differences in electronic and structural changes (vide infra and Supporting Information Section I.3.3 for further discussion), hence best suited to distinguish between proposed structures of Ti sites in TS‐1.

### 3.3 Ti Speciation in TS‐1

**FIGURE 6:**
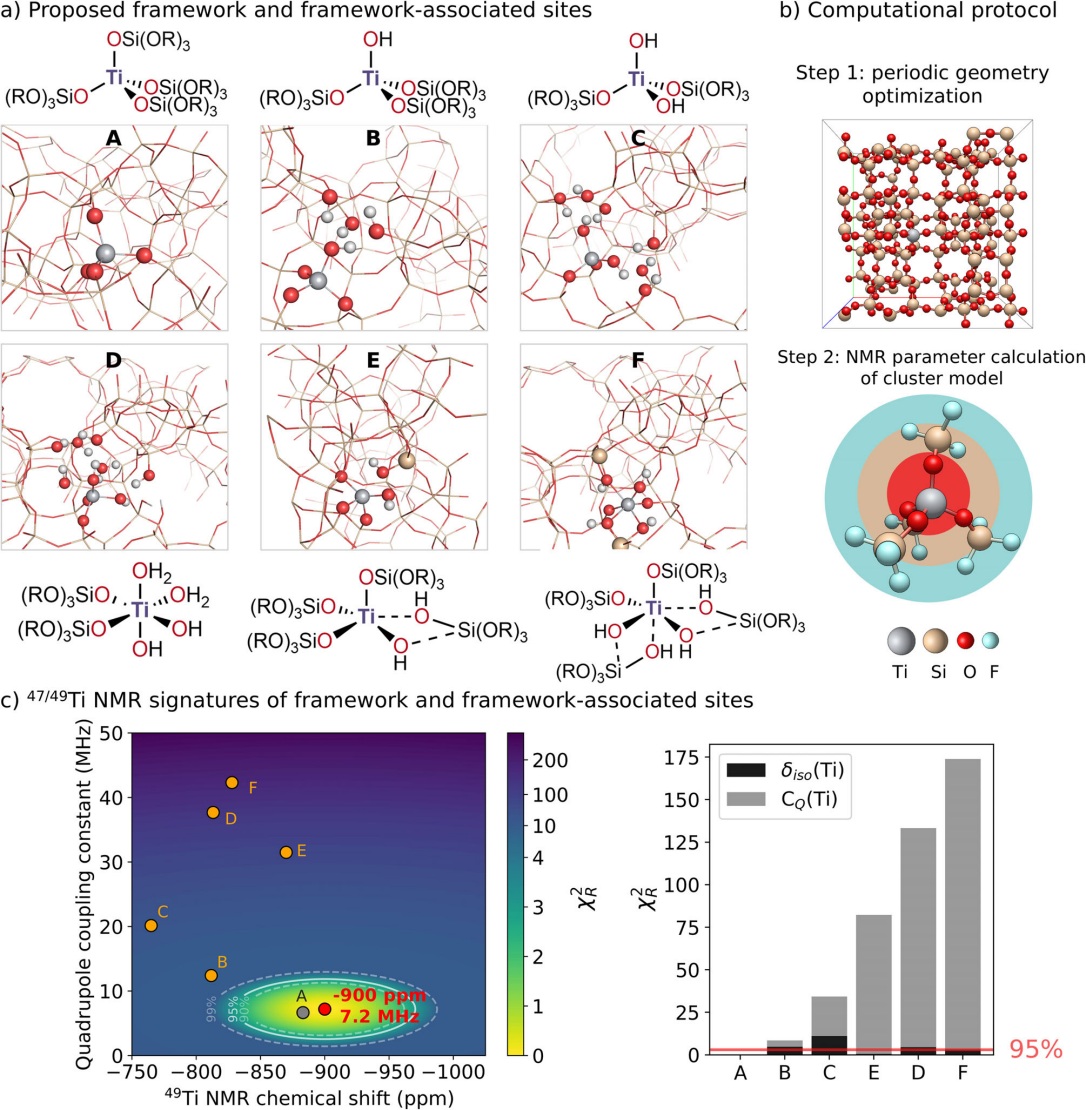
Elucidating Ti‐speciation in TS‐1 via ^47/49^Ti NMR crystallography. (a) Proposed framework and framework‐associated structures. (b) Periodic Silicalite‐1 model with isomorphous substitution of one Si atom with one Ti used for periodic optimization, with the cell parameters adjusted according to the pXRD measurement of the dehydrated material. Cluster model cut from the periodically optimized model and terminated by fluorine atoms. (c) ^49^Ti NMR parameters (isotropic chemical shift and the quadrupole coupling constant) obtained at 28.2 T, 298 K, with the associated *χ_R_* ^2^ around the experimental value of TS‐1 (*left*). Ranking of candidate models based on their *χ_R_* ^2^ statistic (*right*).

We thus explore Ti site speciation from a ^47/49^Ti NMR crystallography perspective, focusing on mononuclear sites considering the aforementioned ^17^O‐results (see Supporting Information Section I.4.2 for discussion and computational analysis). We consider previously proposed structures [22, 79, 99], that is, mononuclear framework and framework‐associated sites (see Figure 6a), as possible structural candidates. This set of sites includes the fully incorporated Ti framework structure (**A**), tetrahedral titanol (**B**) and dititanol (**C**) sites, as well as higher coordination Ti sites, such as hexa‐coordinated dititanol species (**D**), penta‐coordinated bridging titanol (**E**) and hexa‐coordinated bridging dititanol (**F**) sites. Whereas structures **A**—**C** consist of framework or defect sites, structures **D**—**F** are sites which might form upon hydration and/or hydrolysis of Ti─O─Si bond(s).

All models are built by introducing Ti upon isomorphous substitution of one Si atom with one Ti in the Silicalite‐1 unit cell [100] (Figure 6b, equalling ca. Ti 0.8 wt.%), adjusted to the experimental cell parameters obtained from the pXRD measurement (vide supra). Models for defect sites are generated by the selective removal of neighboring Si atoms and the termination of dangling bonds by hydrogen atoms. These periodic structures are used as a starting point to model the Ti‐zeotype and are subjected to a similar two‐step computational protocol discussed above and established for the molecular complexes. Namely, (i) periodic computation of zeolite structures followed by extraction of clusters from the periodic structures and termination by fluorine in the third coordination sphere (see Figure 6b) and (ii) calculation of NMR parameters on the same level of theory as the benchmark set.

In order to visualize the respective computationally predicted NMR parameters of the constructed models, we next construct a 2D *χ_R_* ^2^ heatmap (see Figure 6c) with δ_iso_ and C_Q_ representing the horizontal and vertical axes, respectively. The *z*‐dimension represents the associated reduced *χ* ^2^ value (*χ_R_* ^2^) based on the predicted ^47/49^Ti NMR parameters, while accounting for the global uncertainties and the experimental values of TS‐1 (*red point*, in Figure 6c). The experimental value is surrounded by the 90%, 95%, and 99% confidence limits based on the *χ_R_* ^2^ statistic [39, 43]. This approach is used to visualize confidence threshold values and guide the evaluation of candidate models. Finally, the calculated ^49^Ti isotropic chemical shift and C_Q_ values for the proposed framework and framework‐associated sites in TS‐1 (see Supporting Information for calculated ^49^Ti NMR parameters of the candidate structures, Table S28) are inserted into the 2D heatmap. For clearer visualization of the model selection, the candidate structures are ranked according to their *χ_R_* ^2^ values (agreement with the experimental data, see Section I.5.2 for further details).

For the tetrahedral framework and framework‐associated sites (**A**—**C**) among the pool of candidate structures, the calculated ^49^Ti NMR parameters are found to be closest to experimental observations, namely, quadrupole couplings are below 20 MHz. At the same time, penta‐ and hexacoordinated defect or hydrated sites (**D**—**F**) have large quadrupole couplings, unlike the observed sites. Although the presence of high C_Q_ species (>30 MHz) cannot be ruled out due to sensitivity limitations of ^47/49^Ti NMR, they can only be present in small amounts, based on the Ti K‐edge XAS spectra that point to the presence of mostly tetrahedral sites (see Section S8). Focusing on tetrahedral sites, only the fully framework incorporated Ti site (**A**: δ_iso_(^49^Ti) = −883 ppm C_Q_(^49^Ti) = 6.7 MHz) is associated with δ_iso_(^49^Ti) and C_Q_(^49^Ti) values that lie within the 95% confidence limits of the *χ_R_* ^2^. In contrast, tetrahedral titanol (**B**: δ_iso_(^49^Ti) = −812 ppm C_Q_(^49^Ti) = 12.4 MHz) and dititanol sites (**C**: δ_iso_(^49^Ti) = −765 ppm C_Q_(^49^Ti) = 20.2 MHz) display predicted ^49^Ti NMR parameters that significantly diverge from the experimental finding, in line with our ^17^O NMR results. Thus, the ^47/49^Ti NMR signatures observed for the dehydrated TS‐1 catalyst are assigned to fully incorporated framework sites, corresponding to model **A** (for a discussion related to T‐site position, see below Section *Location of Ti in the framework*).

### 3.4 Distortion of Ti Sites in TS‐1

**FIGURE 7:**
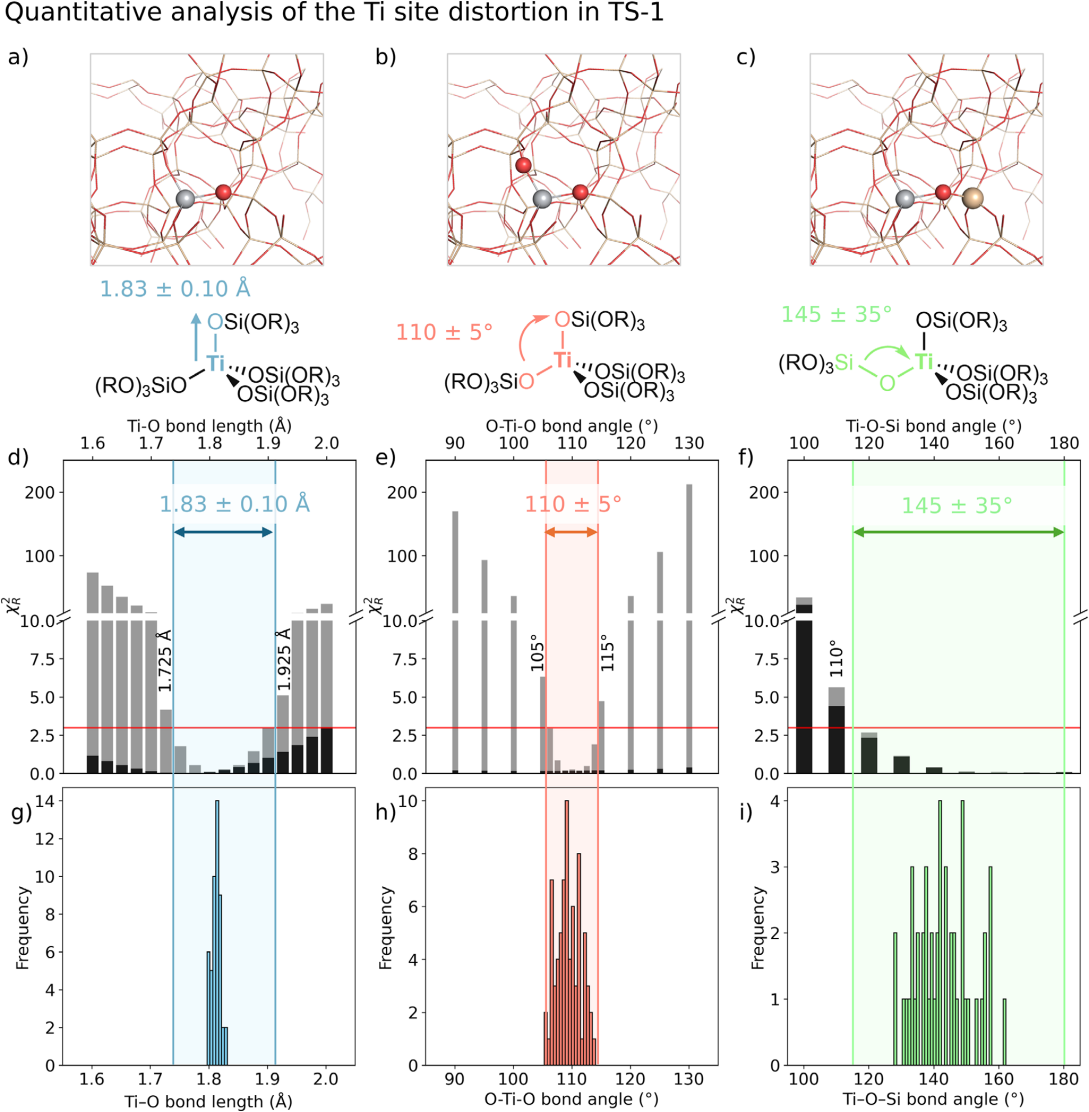
Quantitative analysis of the Ti tetrahedron distortion on a [Ti(OSiF_3_)_4_] model system. (top, a–c) Variation of bond angles and distances of the first and second coordination sphere (middle, d–f) *χ_R_* ^2^ accounting for ^49^Ti NMR parameters (*black*: δ_iso_, *grey*: C_Q_), and the colored interval displays all structures of the geometrical scans within the 95% confidence limit. (bottom, g–i) Respective distribution of bond angles and distances for all T‐sites, extracted from the periodically optimized structures. (a, d, g) Ti─O bond length, (b, e, h) O─Ti─O bond angle, (c, f, i) Ti─O─Si bond angle.

Since the ^47/49^Ti NMR signature is best described by a distribution of fully incorporated Ti framework sites, we next address the effect of structural perturbation on the ^47/49^Ti NMR parameters using a model framework site, namely [Ti(OSiF_3_)_4_] (**A**). The goal of this analysis is to determine cut‐off values for geometrical parameters, which will fall outside the 95% confidence limit (determined by the benchmark set) to describe the experimental values of TS‐1 (δ_iso_(^49^Ti) and C_Q_(^49^Ti), see Figure 7). We denote the interval between these cut‐off values (maximal geometric variation which still describes the experimental values) as the *interval of confidence* for the respective geometric parameter.

First, we computationally investigate the effect of structural changes in the first and second coordination sphere, namely Ti─O bond length, as well as O─Ti─O and Ti─O─Si bond angle variations (see Supporting Information Section I.3.3 for details). Changing one of the Ti─O bond lengths in a range between 1.6 and 2.0 Å has a notable effect on the C_Q_ value, with an increase up to C_Q_ = 30 MHz, while it has a moderate impact on δ_iso_ (all models within 60 ppm of the unperturbed structure **A**). Similarly, δ_iso_ is not responsive (variation of less than 10 ppm) to alterations of the O─Ti─O angle in the range between 90 and 130°, whereas C_Q_ significantly increases (>45 MHz). More notable is that even a slight O─Ti─O distortion (ca. 10°) away from the framework Ti site (model **A**) produces a pronounced change in the quadrupole coupling constant, with C_Q_ values exceeding 20 MHz (see Supporting Information, Section I.3.3 for δ_iso_ and C_Q_ dependency on structural variations). In contrast, changes in the second coordination sphere, manifested in the Ti─O─Si angle between 100 and 180°, lead to rather small changes in C_Q_ (less than 10 MHz compared to the unperturbed structure **A**), whereas increase in the Ti─O─Si angle leads to the decreased chemical shifts of more than 170 ppm, as a consequence of a more facile electron delocalization due to more linear Ti─O─Si angles (modulation of the p‐donation from O to the Ti sites) [18].

Evaluation of the general trends upon geometrical variations enables the determination of the interval of confidence for the respective geometric perturbations, determined by models that fall inside the 95% confidence limits of the reduced *χ* ^2^ (Figure 7d–f). This methodology reveals that the observed Ti sites exhibit the following structural parameters: Ti─O bond length = 1.83 ± 0.10 Å, O─Ti─O bond angle = 110 ± 5°, and Ti─O─Si angle = 145 ± 35° (Figure 7a–c). Note that this analysis neglects the coupling of these parameters (simultaneous change of more than one structural parameter). We note that for future investigations, an extension of the reported method of positional variance determination (developed for small organic molecules) [101], could be considered. Overall, the current analysis shows the sensitivity of ^47/49^Ti NMR signatures to small geometric perturbations, highlighting structural features that are not readily captured and/or resolved by other methods, for example, XAS (K‐ or even L‐edge) [14].

Moreover, it highlights that structural models of TS‐1, which neglect the Ti─O bond length variations and associated angles upon substitution, do not reflect the Ti local environment and are therefore intrinsically limited.

Next, we compare the estimated interval of confidence for the respective geometric perturbations to framework models of Ti in the 12 symmetrically inequivalent T‐sites of the MFI framework (see Supporting Information for details). The extracted geometrical parameters for these models are plotted as histograms (Figure 7g–i) and overlaid with the previously determined intervals of confidence of the conformational space (described by Ti─O bond length, O─Ti─O bond angle, and Ti─O─Si angle, Figure 7d–f). Notably, all Ti─O bond lengths of the framework models are well within the estimated interval of confidence for the Ti─O bond length and are within 1.80–1.83 Å, in line with the previous EXAFS study [99]. For the O─Ti─O bond angle, the spread between the different T‐sites is significantly closer to the maximal estimated bond angle variation, which would still allow for describing the experimental signature, with a variation between all T‐sites of 105–114°. Lastly, the response of the ^47/49^Ti NMR parameters toward variations of the Ti─O─Si angle is shown to be rather insensitive, due to the small response of the quadrupole coupling constant. This is reflected by the finding that, although the spread in the calculated Ti─O─Si angles for all T‐sites is between 127° and 163°, they are all well within the estimated maximum angle variation (cut‐off values). When investigating all three geometric parameters of the T‐site models separately, they are all within the respective intervals of confidence.

However, the combination of perturbations (four Ti─O bond lengths, six O─Ti─O angles, and four Ti─O─Si angles) originating from the strain imposed by the framework is likely to accumulate for given T‐sites. Therefore, models of Ti sites sitting in different T‐site positions of MFI differ on the basis of several distortions at once, reflected in their ^49^Ti NMR parameters.

### 3.5 Location of Ti in the Framework

Next, we employ the same protocol as implemented for the investigation of the Ti speciation in order to evaluate the Ti location in the zeolite framework, focusing on candidate structures, namely the models with Ti located in the 12 symmetrically inequivalent T‐sites of the MFI framework.

**FIGURE 8:**
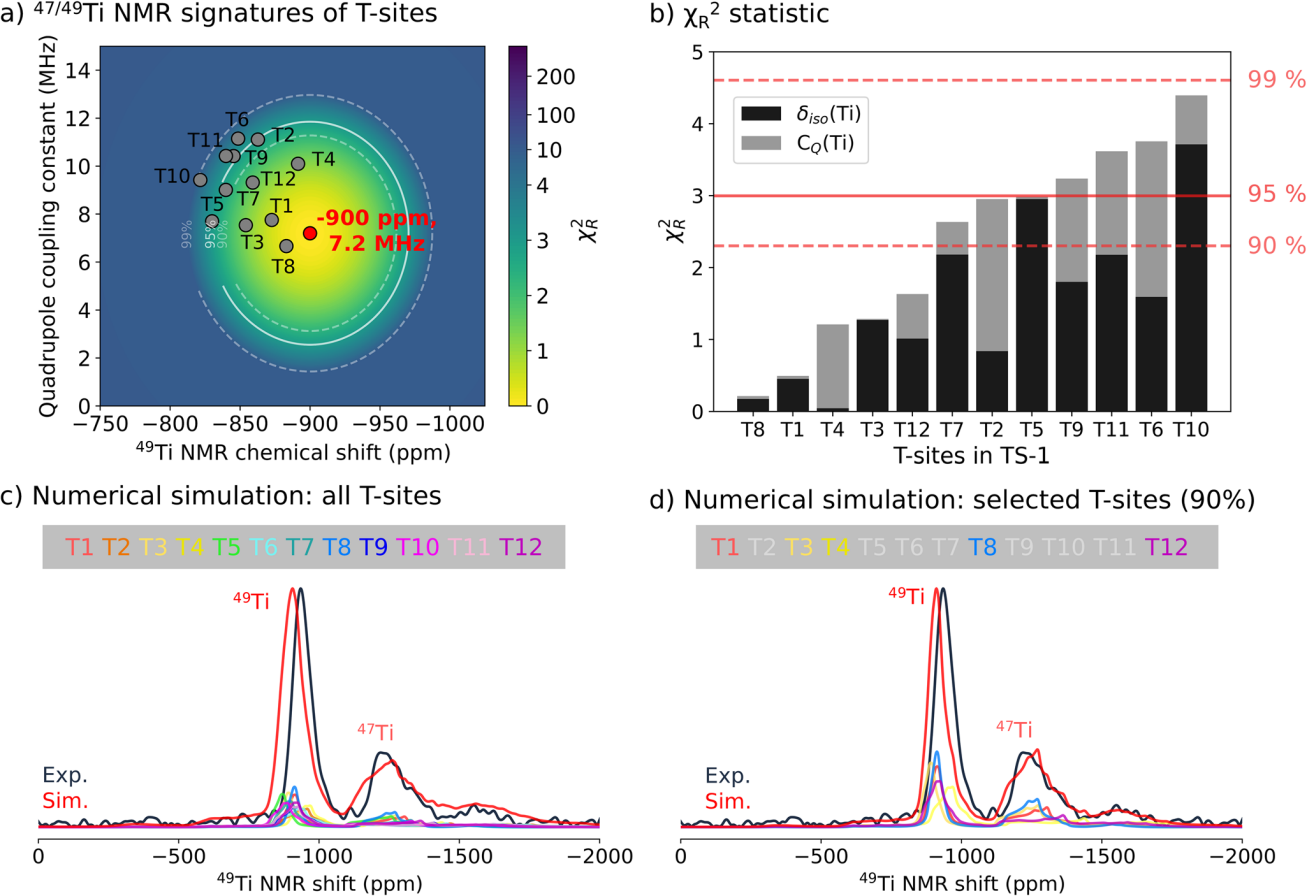
Ti T‐site location(s) in TS‐1 addressed with NMR crystallography. (a) ^49^Ti NMR parameters (NMR chemical shift and the quadrupole coupling constant) obtained at 28.2 T, 298 K, with the associated *χ_R_* ^2^ around the experimental value of TS‐1. (b) T‐sites ordered according to their respective *χ_R_* ^2^ value. (c, d) Comparison between experimental ^47/49^Ti NMR signatures of TS‐1_1.5_ and the numeric simulation based on (c) all T‐sites and (d) selected T‐sites (below the 90% confidence limit threshold), assuming equal population of the considered sites.

Correspondingly, we calculate the respective ^49^Ti isotropic chemical shift and C_Q_ values for the previously constructed models (see Supporting Information for calculated ^49^Ti signatures for all T‐sites, Table S29) and insert them into a 2D *χ_R_* ^2^ heatmap to compare with the experimentally extracted ^49^Ti NMR parameters of the dominant Ti site in TS‐1 (see Figure 8a). As expected, calculations for all 12 T‐sites are generally in good agreement with the experimental ^49^Ti NMR parameters and fall within the 99% confidence interval (all framework structures form a closely related ensemble of structures). The subtle differences are again emphasized by the *χ_R_* ^2^ ranking in Figure 8b. At this point, we note that including additional constraints like ^17^O NMR parameters of the oxygen bonded to the Ti center did not result in a more accurate refinement, since the ^17^O NMR signatures are not sensitive to small Ti‐site perturbations (see Supporting Information Section I.4.3 for further details). Based on the observation that the ^47/49^Ti NMR signature of TS‐1 indicates a distribution of local Ti environments, we perform a numerical simulation considering an equal distribution of all 12 T‐sites, shown in Figure 8c. While this numerical model describes the experimental line shape reasonably well, better agreement can be obtained by only including T‐sites with associated *χ_R_* ^2^ values within the 90% confidence intervals, namely T1, T3, T4, T8, and T12, as demonstrated in Figure 8d (see Supporting Information Section I.3.2.2 for further details and static spectra). These results suggest preferential T‐site occupation in the MFI framework. We note that further refinement might in principle be possible by included weighted population of the different T‐sites. In summary, the experimentally observed ^47/49^Ti NMR signatures depict a dominant Ti site within the investigated TS‐1 samples assigned to framework Ti, which can be further refined considering an equal occupation for a well‐defined set of slightly different environments, most likely originating from the occupancy of several T‐sites, which is in line with our calculations.

## 4 Conclusion

We have developed a ^47/49^Ti NMR crystallography approach aided by ^17^O NMR measurements to understand the speciation of Ti sites as well as to determine their structure and location in TS‐1 in the initial dehydrated state. The high‐field ^47/49^Ti NMR signatures in combination with ^17^O MAT measurements reveal that Ti in TS‐1 occupies isolated mononuclear Ti sites in the pristine state that are fully incorporated into the framework zeolite matrix. The ^47/49^Ti NMR signature is best fitted using an extended Czjzek model, which accounts for a dominant Ti environment perturbed by Czjzek noise. The Czjzek noise can be interpreted as small variations in the first and second coordination spheres. These variations originate either from (i) small perturbations of the Ti environment in one T‐site, (ii) a distribution of Ti across several T‐sites, or (iii) a combination of both effects. Based on the observation that the ^47/49^Ti NMR signatures originate from presumably more than one site, we computationally evaluated site perturbations in the first and second coordination spheres, which would still be able to describe the experimental finding. This gives rise to specific intervals of confidence for geometrical perturbations of the Ti sites in TS‐1: Ti─O = 1.83 ± 0.10 Å, O─Ti─O = 110 ± 5°, and Ti─O─Si = 145 ± 35°. These site variations are in agreement with the occupation of several framework T‐site positions by Ti. Numeric simulations for either all T‐sites occupied or preferential T‐site occupation enable the capture of the observed ^47/49^Ti NMR signatures. More specifically, based on the numeric simulations and a 90% confidence limit in the *χ_R_* ^2^ statistics, a preferential T‐site occupation (across T1, T3, T4, T8, and T12) is suggested.

We have also shown that the use of ^47/49^Ti NMR spectroscopy across samples containing various Ti loadings shows that the dominant Ti environment is independent of the amount of Ti as long as no extra‐framework TiO_2_ is present. Furthermore, we show that ^47/49^Ti NMR spectroscopy at high field also enables the resolution of framework and extra‐framework sites in hierarchical TS‐1.

Overall, we have developed a ^47/49^Ti NMR crystallography approach that provides ways to understand the speciation and the local Ti structure of industrially relevant TS‐1 catalysts, and we are currently further exploring the universality of these findings and expanding the NMR crystallography approach to other zeotypes.

## Conflicts of Interest

The authors declare no conflicts of interest.

## Supporting information




**Supporting File 1**: Zeolite synthesis, zeolite characterization and catalytic performance, synthesis protocols of molecular library and corresponding solution NMR spectra, single‐crystal XRD structures, solution 47/49Ti NMR spectra of molecular library, Ti K‐edge XANES analysis of molecular library and zeotypes, powder XRD measurements, 17O and 47/49Ti solid‐state NMR measurements and fitting parameters, DFT calculation protocols for periodic geometry optimization and cluster NMR parameters calculations.

**Supporting File 2**: anie71223‐sup‐0002‐Data.zip.

## Data Availability

The data that support the findings of this study are available from the corresponding author upon reasonable request. CCDC 2482638 contains the supplementary crystallographic data for this paper. This data can be obtained free of charge from The Cambridge Crystallographic Data Centre via www.ccdc.cam.ac.uk/data_request/cif.

## References

[1] M. Taramasso , G. Perego , and B. Notari , (1983), Preparation of Porous Crystalline Synthetic Material Comprised of Silicon and Titanium Oxides. US4410501A.

[2] V. Smeets , E. M. Gaigneaux , and D. P. Debecker , “Titanosilicate Epoxidation Catalysts: A Review of Challenges and Opportunities,” Chemcatchem 14, no. 1 (2022), 10.1002/cctc.202101132.

[3] G. N. Vayssilov , “Structural and Physicochemical Features of Titanium Silicalites,” Catalysis Reviews 39, no. 3 (1997): 209–251, 10.1080/01614949709353777.

[4] B. Wang , Y. Guo , J. Zhu , J. Ma , and Q. Qin , “A Review on Titanosilicate‐1 (TS‐1) Catalysts: Research Progress of Regulating Titanium Species,” Coordination Chemistry Reviews 476 (2023): 214931, 10.1016/j.ccr.2022.214931.

[5] M. Lin , C. Xia , B. Zhu , H. Li , and X. Shu , “Green and Efficient Epoxidation of Propylene With Hydrogen Peroxide (HPPO Process) Catalyzed by Hollow TS‐1 Zeolite: A 1.0 Kt/a Pilot‐Scale Study,” Chemical Engineering Journal 295 (2016): 370–375, 10.1016/j.cej.2016.02.072.

[6] H. Xu and P. Wu , “Recent Progresses in Titanosilicates,” Chinese Journal of Chemistry 35, no. 6 (2017): 836–844, 10.1002/cjoc.201600739.

[7] Z. Li , R. Chen , W. Xing , W. Jin , and N. Xu , “Continuous Acetone Ammoximation Over TS‐1 in a Tubular Membrane Reactor,” Industrial & Engineering Chemistry Research 49, no. 14 (2010): 6309–6316, 10.1021/ie901912e.

[8] Q. Ge , J. Lu , and M. Zhu , “Research Progress of Titanium Silicalite Molecular Sieve for Cyclohexanone Ammoximation,” Hecheng Xiangjiao Gongye 38, no. 1 (2015): 54–58.

[9] M. Li , J. Liu , and H. Na , “Application and Advancements in Synthesis of 2‐Butanone Oxime,” Huaxue Gongchengshi 20, no. 7 (2006): 42–43.

[10] R. Millini , “Framework Composition of Titanium Silicalite‐1,” Journal of Catalysis 137, no. 2 (1992): 497–503, 10.1016/0021-9517(92)90176-I.

[11] G. Peregot , G. Bellussi , C. Corno , M. Taramasso , F. Buonomot , and A. Esposito , “Titanium‐Silicalite: A Novel Derivative in the Pentasil Family,” Studies in Surface Science and Catalysis 28 (1986): 129–136, 10.1016/S0167-2991(09)60865-X.

[12] S. Bordiga , F. Boscherini , S. Coluccia , et al., “XAFS Study of Ti‐silicalite: Structure of Framework Ti(IV) in Presence and in Absence of Reactive Molecules (H_2_O, NH_3_),” Catal Letters 26, no. 1–2 (1994): 195–208, 10.1007/BF00824045.

[13] S. Pei , G. W. Zajac , J. A. Kaduk , et al., “Re‐Investigation of Titanium Silicalite by X‐Ray Absorption Spectroscopy: Are the Novel Titanium Sites Real?,” Catal Letters 21, no. 3–4 (1993): 333–344, 10.1007/BF00769485.

[14] L. Lätsch , S. A. Guda , V. Romankov , et al., “Tracking Coordination Environment and Reaction Intermediates in Homogeneous and Heterogeneous Epoxidation Catalysts via Ti L_2,3_ ‐Edge Near‐Edge X‐ray Absorption Fine Structures,” Journal of the American Chemical Society 146, no. 11 (2024): 7456–7466, 10.1021/jacs.3c12831. 38447178

[15] G. Ricchiardi , A. Damin , S. Bordiga , et al., “Vibrational Structure of Titanium Silicate Catalysts. A Spectroscopic and Theoretical Study,” Journal of the American Chemical Society 123, no. 46 (2001): 11409–11419, 10.1021/ja010607v. 11707118

[16] M. R. Boccuti , K. M. Rao , A. Zecchina , G. Leofanti , and G. Petrini , “Spectroscopic Characterization of Silicalite and Titanium‐Silicalite,” Studies in Surface Science and Catalysis 48 (1989): 133–144, 10.1016/S0167-2991(08)60677-1.

[17] F. Geobaldo , S. Bordiga , A. Zecchina , E. Giamello , G. Leofanti , and G. Petrini , “DRS UV‐Vis and EPR Spectroscopy of Hydroperoxo and Superoxo Complexes in Titanium Silicalite,” Catal Letters 16, no. 1–2 (1992): 109–115, 10.1007/BF00764360.

[18] L. Lätsch , C. J. Kaul , A. V. Yakimov , et al., “NMR Signatures and Electronic Structure of Ti Sites in Titanosilicalite‐1 From Solid‐State ^47/49^Ti NMR Spectroscopy,” Journal of the American Chemical Society 145, no. 28 (2023): 15018–15023, 10.1021/jacs.2c09867. 37418311

[19] W. O. Parker and R. Millini , “Ti Coordination in Titanium Silicalite‐1,” Journal of the American Chemical Society 128, no. 5 (2006): 1450–1451, 10.1021/ja0576785. 16448106

[20] A. J. H. P. van der Pol , A. J. Verduyn , and J. H. C. van Hooff , “Why Are some Titanium Silicalite‐1 Samples Active and Others Not?,” Applied Catalysis A: General 92, no. 2 (1992): 113–130, 10.1016/0926-860X(92)80310-9.

[21] C. Baerlocher , D. Brouwer , B. Marler , and L. B. McCusker , https://www.iza‐structure.org/databases/, *Database of Zeolite Structures*.

[22] Q. Guo , K. Sun , Z. Feng , et al., “A Thorough Investigation of the Active Titanium Species in TS‐1 Zeolite by in Situ UV Resonance Raman Spectroscopy,” Chemistry—A European Journal 18, no. 43 (2012): 13854–13860, 10.1002/chem.201201319. 22969000

[23] C. Lamberti , S. Bordiga , A. Zecchina , et al., “Structural Characterization of Ti‐Silicalite‐1: A Synchrotron Radiation X‐Ray Powder Diffraction Study,” Journal of Catalysis 183, no. 2 (1999): 222–231, 10.1006/jcat.1999.2403.

[24] C. A. Hijar , R. M. Jacubinas , J. Eckert , N. J. Henson , P. J. Hay , and K. C. Ott , “The Siting of Ti in TS‐1 Is Non‐Random. Powder Neutron Diffraction Studies and Theoretical Calculations of TS‐1 and FeS‐1,” Journal of Physical Chemistry B 104, no. 51 (2000): 12157–12164, 10.1021/jp002167k.

[25] P. F. Henry , M. T. Weller , and C. C. Wilson , “Structural Investigation of TS‐1: Determination of the True Nonrandom Titanium Framework Substitution and Silicon Vacancy Distribution From Powder Neutron Diffraction Studies Using Isotopes,” Journal of Physical Chemistry B 105, no. 31 (2001): 7452–7458, 10.1021/jp0107715.

[26] C. Lamberti , S. Bordiga , A. Zecchina , G. Artioli , G. Marra , and G. Spanò , “Ti Location in the MFI Framework of Ti−Silicalite‐1: A Neutron Powder Diffraction Study,” Journal of the American Chemical Society 123, no. 10 (2001): 2204–2212, 10.1021/ja003657t. 11456866

[27] P. Rzepka , M. Signorile , T. Huthwelker , et al., “Quantitative Localisation of Titanium in the Framework of Titanium Silicalite‐1 Using Anomalous X‐ray Powder Diffraction,” Nature Communications 15, no. 1 (2024): 7757, 10.1038/s41467-024-51788-7. PMC11377426 39237487

[28] J. Dong , H. Zhu , Y. Xiang , et al., “Toward a Unified Identification of Ti Location in the MFI Framework of High‐Ti‐Loaded TS‐1: Combined EXAFS, XANES, and DFT Study,” The Journal of Physical Chemistry C 120, no. 36 (2016): 20114–20124, 10.1021/acs.jpcc.6b05087.

[29] M. Signorile , A. Damin , F. Bonino , et al., “Computational Assessment of Relative Sites Stabilities and Site‐Specific Adsorptive Properties of Titanium Silicalite‐1,” The Journal of Physical Chemistry C 122, no. 3 (2018): 1612–1621, 10.1021/acs.jpcc.7b10104.

[30] G. Ricchiardi , A. de Man , and J. Sauer , “The Effect of Hydration on Structure and Location of Ti‐Sites in Ti‐Silicalite Catalysts. A Computational Study,” Physical Chemistry Chemical Physics 2, no. 10 (2000): 2195–2204, 10.1039/a909617e.

[31] R. C.h. Deka , V. A. Nasluzov , E. A. Ivanova Shor , A. M. Shor , G. N. Vayssilov , and N. Rösch , “Comparison of all Sites for Ti Substitution in Zeolite TS‐1 by an Accurate Embedded‐Cluster Method,” Journal of Physical Chemistry B 109, no. 51 (2005): 24304–24310, 10.1021/jp050056l. 16375428

[32] J. D. Gale , “A Periodic Density Functional Study of the Location of Titanium Within TS‐1,” Solid State Sciences 8, no. 3–4 (2006): 234–240, 10.1016/j.solidstatesciences.2006.02.011.

[33] S. L. Njo , H. van Koningsveld , and B. van de Graaf , “A Combination of the Monte Carlo Method and Molecular Mechanics Calculations: A Novel Way To Study the Ti(IV) Distribution in Titanium Silicalite‐1,” Journal of Physical Chemistry B 101, no. 48 (1997): 10065–10068, 10.1021/jp971451h.

[34] J. C. Facelli and D. M. Grant , “Determination of Molecular Symmetry in Crystalline Naphthalene Using Solid‐State NMR,” Nature 365, no. 6444 (1993): 325–327, 10.1038/365325a0. 8377823

[35] C. Martineau , J. Senker , and F. Taulelle , “NMR Crystallography,” Annual Reports on NMR Spectroscopy 82 (2014): 1–57, 10.1016/B978-0-12-800184-4.00001-1.

[36] R. K. N. M. R. Harris , “NMR Crystallography: The Use of Chemical Shifts,” Solid State Sciences 6, no. 10 (2004): 1025–1037, 10.1016/j.solidstatesciences.2004.03.040.

[37] S. E. Ashbrook and D. McKay , “Combining Solid‐State NMR Spectroscopy With First‐principles Calculations—A Guide to NMR Crystallography,” Chemical Communications 52, no. 45 (2016): 7186–7204, 10.1039/C6CC02542K. 27117884

[38] R. K. Harris , R. D. Wasylishen , and M. J. Duer , NMR Crystallography (John Wiley & Sons Ltd: Chichester, 2009).

[39] L. J. Mueller , “Uniform Chi‐Squared Model Probabilities in NMR Crystallography,” Faraday Discussions 255, no. 0 (2025): 203–221, 10.1039/D4FD00114A. 39311003 PMC11710992

[40] L. J. Mueller and M. F. Dunn , “NMR Crystallography of Enzyme Active Sites: Probing Chemically Detailed, Three‐Dimensional Structure in Tryptophan Synthase,” Accounts of Chemical Research 46, no. 9 (2013): 2008–2017, 10.1021/ar3003333. 23537227 PMC3778090

[41] J. B. Holmes , V. Liu , B. G. Caulkins , et al., “Imaging Active Site Chemistry and Protonation States: NMR Crystallography of the Tryptophan Synthase α‐Aminoacrylate Intermediate,” Proceedings of the National Academy of Sciences, no. 2 (2022): 119, 10.1073/pnas.2109235119. PMC8764694 34996869

[42] Bryce, D. L. , ed., Modern NMR Crystallography (Royal Society of Chemistry, 2025), 10.1039/9781837673179.

[43] B. G. Caulkins , R. P. Young , R. A. Kudla , et al., “NMR Crystallography of a Carbanionic Intermediate in Tryptophan Synthase: Chemical Structure, Tautomerization, and Reaction Specificity,” Journal of the American Chemical Society 138, no. 46 (2016): 15214–15226, 10.1021/jacs.6b08937. 27779384 PMC5129030

[44] Brouwer , R. J. Darton , R. E. Morris , and M. H. Levitt , “A Solid‐State NMR Method for Solution of Zeolite Crystal Structures,” Journal of the American Chemical Society 127, no. 29 (2005): 10365–10370, 10.1021/ja052306h. 16028949

[45] D. H. Brouwer , S. Cadars , J. Eckert , Z. Liu , O. Terasaki , and B. F. Chmelka , “A General Protocol for Determining the Structures of Molecularly Ordered but Noncrystalline Silicate Frameworks,” Journal of the American Chemical Society 135, no. 15 (2013): 5641–5655, 10.1021/ja311649m. 23560776

[46] Z. J. Berkson , W. Cao , D. Gioffrè , et al., “NMR Signatures of Transition‐Metal Nuclei: From Local Environments and Electronic Structures to Reactivity Descriptors in Molecular and Heterogeneous Catalysis,” JACS Au 5, no. 7 (2025): 2911–2931, 10.1021/jacsau.5c00061. 40747012 PMC12308410

[47] G. Bellussi , “Reactions of Titanium Silicalite With Protic Molecules and Hydrogen Peroxide,” Journal of Catalysis 133, no. 1 (1992): 220–230, 10.1016/0021-9517(92)90199-R.

[48] L. Lätsch , I. B. Müller , C. J. Kaul , et al., “Formation and Stability of μ_2_‐Peroxo on Titanosilicates, Anatase, and Rutile: Implications for Zeotype Catalysts,” The Journal of Physical Chemistry C 128, no. 13 (2024): 5553–5558, 10.1021/acs.jpcc.3c08292.

[49] S. M. Pugh , P. A. Wright , D. J. Law , N. Thompson , and S. E. F. Ashbrook , “Facile, Room‐Temperature ^17^O Enrichment of Zeolite Frameworks Revealed by Solid‐State NMR Spectroscopy,” Journal of the American Chemical Society 142, no. 2 (2020): 900–906, 10.1021/jacs.9b10528. 31875398

[50] S. E. Ashbrook , Z. H. Davis , R. E. Morris , and C. M. Rice , “ ^17^O NMR Spectroscopy of Crystalline Microporous Materials,” Chemical Science 12, no. 14 (2021): 5016–5036, 10.1039/D1SC00552A. 34163746 PMC8179582

[51] B. E. G. Lucier and Y. Huang , “Reviewing 47/49 Solid‐State NMR Spectroscopy: From Alloys and Simple Compounds to Catalysts and Porous Materials,” Annual Reports on NMR Spectroscopy 88 (2016): 1–78, 10.1016/bs.arnmr.2015.10.001.

[52] D. Padro , V. Jennings , M. E. Smith , R. Hoppe , P. A. Thomas , and R. Dupree , “Variations of Titanium Interactions in Solid State NMRCorrelations to Local Structure,” Journal of Physical Chemistry B 106, no. 51 (2002): 13176–13185, 10.1021/jp021583x.

[53] A. J. Rossini , I. Hung , and R. W. Schurko , “Solid‐State^47/49^ Ti NMR of Titanocene Chlorides,” Journal of Physical Chemistry Letters 1, no. 20 (2010): 2989–2998, 10.1021/jz1012017.

[54] C. Gervais , M. E. Smith , A. Pottier , J.‐P. Jolivet , and F. Babonneau , “Solid‐State^47,49^ Ti NMR Determination of the Phase Distribution of Titania Nanoparticles,” Chemistry of Materials 13, no. 2 (2001): 462–467, 10.1021/cm0011918.

[55] J. Xu , B. E. G. Lucier , Z. Lin , A. Sutrisno , V. V. Terskikh , and Y. Huang , “New Insights Into the Short‐Range Structures of Microporous Titanosilicates As Revealed by^47/49^Ti,^23^Na,^39^K, and^29^Si Solid‐State NMR Spectroscopy,” The Journal of Physical Chemistry C 118, no. 47 (2014): 27353–27365, 10.1021/jp5077966.

[56] K. J. Stephens , G. Zichittella , A. J. Saadun , et al., “Transformation of Titanium Carbide Into Mesoporous Titania for Catalysed HBr Oxidation,” Catalysis Science & Technology 10, no. 12 (2020): 4072–4083, 10.1039/D0CY00805B.

[57] E. S. Blaakmeer (M) , F. J. Wensink , E. R. H. van Eck , G. A. de Wijs , and A. P. M. Kentgens , “Preactive Site in Ziegler–Natta Catalysts,” The Journal of Physical Chemistry C 123, no. 23 (2019): 14490–14500, 10.1021/acs.jpcc.9b02617.

[58] A. V. Yakimov , C. J. Kaul , Y. Kakiuchi , et al., “Well‐Defined Ti Surface Sites in Ziegler–Natta Pre‐Catalysts from^47/49^Ti Solid‐State Nuclear Magnetic Resonance Spectroscopy,” Journal of Physical Chemistry Letters 15, no. 11 (2024): 3178–3184, 10.1021/acs.jpclett.3c03119. 38478985

[59] S. Ganapathy , K. U. Gore , R. Kumar , and J.‐P. Amoureux , “Multinuclear (^27^Al, ^29^Si, ^47,49^Ti) Solid‐State NMR of Titanium Substituted Zeolite USY,” Solid State Nuclear Magnetic Resonance 24, no. (2–3) (2003): 184–195, 10.1016/S0926-2040(03)00044-4. 12943913

[60] A. Lopez , M. H. Tuilier , J. L. Guth , L. Delmotte , and J. M. Popa , “Titanium in MFI‐Type Zeolites: A Characterization by XANES, EXAFS, IR, and ^74,49^Ti and ^17^O MAS NMR Spectroscopy and H_2_O Adsorption,” Journal of Solid State Chemistry 102, no. 2 (1993): 480–491, 10.1006/jssc.1993.1060.

[61] R. K. Harris , E. D. Becker , S. M. Cabral de Menezes , R. Goodfellow , and P. Granger , “NMR Nomenclature: Nuclear Spin Properties and Conventions for Chemical Shifts,” Solid State Nuclear Magnetic Resonance 22, no. 4 (2002): 458–483, 10.1006/snmr.2002.0063. 12637147

[62] C. P. Gordon , H. Engler , A. S. Tragl , et al., “Efficient Epoxidation Over Dinuclear Sites in Titanium Silicalite‐1,” Nature 586, no. 7831 (2020): 708–713, 10.1038/s41586-020-2826-3. 33116285

[63] M. H. Cohen and F. Reif , “Quadrupole Effects in Nuclear Magnetic Resonance Studies of Solids,” Solid State Physics—Advances in Research and Applications 5, no. C (1957), 10.1016/S0081-1947(08)60105-8.

[64] A. P. M. Kentgens and R. Verhagen , “Advantages of Double Frequency Sweeps in Static, MAS and MQMAS NMR of Spin I=3/2 Nuclei,” Chemical Physics Letters 300, no. 3–4 (1999): 435–443, 10.1016/S0009-2614(98)01402-X.

[65] D. Iuga , H. Schäfer , R. Verhagen , and A. P. M. Kentgens , “Population and Coherence Transfer Induced by Double Frequency Sweeps in Half‐Integer Quadrupolar Spin Systems,” Journal of Magnetic Resonance 147, no. 2 (2000): 192–209, 10.1006/jmre.2000.2192. 11097810

[66] F. H. Larsen , H. J. Jakobsen , P. D. Ellis , and N. C. Nielsen , “QCPMG‐MAS NMR of Half‐Integer Quadrupolar Nuclei,” Journal of Magnetic Resonance 131, no. 1 (1998): 144–147, 10.1006/jmre.1997.1341. 9533917

[67] H. Y. Carr and E. M. Purcell , “Effects of Diffusion on Free Precession in Nuclear Magnetic Resonance Experiments,” Physical Review 94, no. 3 (1954): 630–638, 10.1103/PhysRev.94.630.

[68] S. Meiboom and D. Gill , “Modified Spin‐Echo Method for Measuring Nuclear Relaxation Times,” Review of Scientific Instruments 29, no. 8 (1958): 688–691, 10.1063/1.1716296.

[69] L. A. O'Dell and R. W. Schurko , “QCPMG Using Adiabatic Pulses for Faster Acquisition of Ultra‐Wideline NMR Spectra,” Chemical Physics Letters 464, no. 1–3 (2008): 97–102, 10.1016/j.cplett.2008.08.095.

[70] J. Koppe , J. E. Frerichs , and M. R. Hansen , “Pushing the Detection Limit of Static Wideline NMR Spectroscopy Using Ultrafast Frequency‐Swept Pulses,” Journal of Physical Chemistry Letters 14, no. 48 (2023): 10748–10753, 10.1021/acs.jpclett.3c02758. 38010530

[71] R. Bhattacharyya and L. Frydman , “Quadrupolar Nuclear Magnetic Resonance Spectroscopy in Solids Using Frequency‐Swept Echoing Pulses,” Journal of Chemical Physics 127, no. 19 (2007), 10.1063/1.2793783. 18035888

[72] E. Kupce and R. Freeman , “Adiabatic Pulses for Wideband Inversion and Broadband Decoupling,” Journal of Magnetic Resonance A 115, no. 2 (1995): 273–276, 10.1006/jmra.1995.1179.

[73] J. Z. Hu , D. W. Alderman , C. H. Ye , R. J. Pugmire , and D. M. Grant , “An Isotropic Chemical Shift‐Chemical Shift Anisotropy Magic‐Angle Slow‐Spinning 2D NMR Experiment,” Journal of Magnetic Resonance A 105, no. 1 (1993): 82–87, 10.1006/jmra.1993.1252.

[74] C. Gervais , F. Babonneau , D. Hoebbel , and M. E. Smith , “Solid State NMR Interaction Parameters of Oxygens Linking Titanium and Silicon in Crystalline Cyclic Titanodiphenylsiloxanes,” Solid State Nuclear Magnetic Resonance 17, no. 1–4 (2000): 2–14, 10.1006/snmr.2000.0001. 11235025

[75] E. Scolan , C. Magnenet , D. Massiot , and C. Sanchez , “Surface and Bulk Characterisation of Titanium–oxo Clusters and Nanosized Titania Particles Through ^17^O Solid state NMR,” Journal of Materials Chemistry 9, no. 10 (1999): 2467–2474, 10.1039/a903714d.

[76] E. R. H. van Eck , M. E. Smith , and S. C. Kohn , “Observation of Hydroxyl Groups by Solid‐state Multiple Quantum MAS NMR in Sol–gel‐produced Silica,” Solid State Nuclear Magnetic Resonance 15, no. 3 (1999): 181–188, 10.1016/S0926-2040(99)00055-7. 10672942

[77] Y. Li , X.‐P. Wu , N. Jiang , et al., “Distinguishing Faceted Oxide Nanocrystals With ^17^O Solid‐State NMR Spectroscopy,” Nature Communications 8, no. 1 (2017): 581, 10.1038/s41467-017-00603-7. PMC5603560 28924155

[78] J. Koppe , K. J. Sanders , T. C. Robinson , et al., “Resolving Structures of Paramagnetic Systems in Chemistry and Materials Science by Solid‐State NMR: The Revolving Power of Ultra‐Fast MAS,” Angewandte Chemie International Edition 64, no. 1 (2025): e202408704, 10.1002/anie.202408704. 39388344 PMC11701365

[79] L. Lätsch , C. J. Kaul , A. V. Yakimov , et al., “Nature of Reactive Sites in TS‐1 From^15^N Solid‐State NMR and Ti K‐Edge X‐Ray Absorption Spectroscopic Signatures Upon Pyridine Adsorption,” Journal of the American Chemical Society 146, no. 43 (2024): 29675–29683, 10.1021/jacs.4c10604. 39428628

[80] G. L.e Caër , B. Bureau , and D. Massiot , “An Extension of the Czjzek Model for the Distributions of Electric Field Gradients in Disordered Solids and an Application to NMR Spectra of 71Ga in Chalcogenide Glasses,” Journal of Physics: Condensed Matter 22, no. 6 (2010): 065402, 10.1088/0953-8984/22/6/065402. 21389367

[81] F. Vasconcelos , S. Cristol , J.‐F. Paul , et al., “Extended Czjzek Model Applied to NMR Parameter Distributions in Sodium Metaphosphate Glass,” Journal of Physics: Condensed Matter 25, no. 25 (2013): 255402, 10.1088/0953-8984/25/25/255402. 23719213

[82] M. Bak , J. T. Rasmussen , and N. C. S. Nielsen , “SIMPSON: A General Simulation Program for Solid‐State NMR Spectroscopy,” Journal of Magnetic Resonance 147, no. 2 (2000): 296–330, 10.1006/jmre.2000.2179. 11097821

[83] T. Weissenberger , R. Leonhardt , B. A. Zubiri , et al., “Synthesis and Characterisation of Hierarchically Structured Titanium Silicalite‐1 Zeolites With Large Intracrystalline Macropores,” Chemistry—A European Journal 25, no. 63 (2019): 14430–14440, 10.1002/chem.201903287. 31478582

[84] R. Sanz , D. P. Serrano , P. Pizarro , and I. Moreno , “Hierarchical TS‐1 Zeolite Synthesized From SiO_2_ TiO_2_ Xerogels Imprinted With Silanized Protozeolitic Units,” Chemical Engineering Journal 171, no. 3 (2011): 1428–1438, 10.1016/j.cej.2011.02.036.

[85] A. Okuniewski , D. Rosiak , J. Chojnacki , and B. Becker , “Coordination Polymers and Molecular Structures Among Complexes of Mercury(II) Halides With Selected 1‐Benzoylthioureas,” Polyhedron 90 (2015): 47–57, 10.1016/j.poly.2015.01.035.

[86] G. Noh , E. Lam , J. L. Alfke , et al., “Selective Hydrogenation of CO_2_ to CH_3_OH on Supported Cu Nanoparticles Promoted by Isolated Ti^IV^ Surface Sites on SiO_2_ ,” Chemsuschem 12, no. 5 (2019): 968–972, 10.1002/cssc.201900134. 30644172

[87] J. Pérez‐Pérez , A. Gallardo‐Garibay , D. Martínez‐Otero , U. Hernández‐Balderas , and V. Jancik , “Formation of Titanosilicate N,N‐Dialkyl Carbamates by CO_2_ Insertion Into the [(TBuO)_3_SiO]_3_TiNR_2_ Scaffold,” European Journal of Inorganic Chemistry 27, no. 28 (2024): e202400337, 10.1002/ejic.202400337.

[88] L. Pauling , “The Nature of the Chemical Bond. IV. The Energy of Single Bonds and the Relative Electronegativity of Atoms,” Journal of the American Chemical Society 54, no. 9 (1932): 3570–3582, 10.1021/ja01348a011.

[89] E. S. Kadantsev , R. Klooster , P. L. De Boeij , and T. Ziegler , “The Formulation and Implementation of Analytic Energy Gradients for Periodic Density Functional Calculations With STO/NAO Bloch Basis Set,” Molecular Physics 105, no. 19–22 (2007): 2583–2596, 10.1080/00268970701598063.

[90] BAND 2023.101. SCM, Theoretical Chemistry, Vrije Universiteit: Amsterdam, The Netherlands 2023, http://www.scm.com (accessed 2025‐01‐28).

[91] A. D. Becke , “Density‐Functional Exchange‐Energy Approximation With Correct Asymptotic Behavior,” Physical Review A (Coll Park) 38, no. 6 (1988): 3098–3100, 10.1103/PhysRevA.38.3098. 9900728

[92] J. P. Perdew , “Density‐Functional Approximation for the Correlation Energy of the Inhomogeneous Electron Gas,” Physical Review B 33, no. 12 (1986): 8822–8824, 10.1103/PhysRevB.33.8822. 9938299

[93] E. Van Lenthe and E. J. Baerends , “Optimized Slater‐Type Basis Sets for the Elements 1–118,” Journal of Computational Chemistry 24, no. 9 (2003): 1142–1156, 10.1002/jcc.10255. 12759913

[94] G. Schreckenbach and T. Ziegler , “Calculation of NMR Shielding Tensors Using Gauge‐Including Atomic Orbitals and Modern Density Functional Theory,” Journal of Physical Chemistry 99, no. 2 (1995): 606–611, 10.1021/j100002a024.

[95] M. Krykunov , T. Ziegler , and E. v. Lenthe , “Hybrid Density Functional Calculations of Nuclear Magnetic Shieldings Using Slater‐Type Orbitals and the Zeroth‐Order Regular Approximation,” International Journal of Quantum Chemistry 109, no. 8 (2009): 1676–1683, 10.1002/qua.21985.

[96] E. van Lenthe and E. Jan Baerends , “Density Functional Calculations of Nuclear Quadrupole Coupling Constants in the Zero‐Order Regular Approximation for Relativistic Effects,” Journal of Chemical Physics 112, no. 19 (2000): 8279–8292, 10.1063/1.481433.

[97] ADF 2023.101. SCM, Theoretical Chemistry, Vrije Universiteit: Amsterdam, The Netherlands 2023, http://www.scm.com (accessed 2025‐01‐28).

[98] C. Adamo and V. Barone , “Toward Reliable Density Functional Methods Without Adjustable Parameters: The PBE0 Model,” Journal of Chemical Physics 110, no. 13 (1999): 6158–6170, 10.1063/1.478522.

[99] D. Gleeson , G. Sankar , C. Richard , et al., “The Architecture of Catalytically Active Centers in Titanosilicate (TS‐1) and Related Selective‐Oxidation Catalysts,” Physical Chemistry Chemical Physics 2, no. 20 (2000): 4812–4817, 10.1039/b005780k.

[100] G. Artioli , C. Lamberti , and G. L. Marra , “Neutron Powder Diffraction Study of Orthorhombic and Monoclinic Defective Silicalite,” Acta Crystallographica. Section B, Structural Science 56, no. 1 (2000): 2–10, 10.1107/S0108768199008927. 10735438

[101] A. Hofstetter and L. Emsley , “Positional Variance in NMR Crystallography,” Journal of the American Chemical Society 139, no. 7 (2017): 2573–2576, 10.1021/jacs.6b12705. 28146348

