# Supplementary material for: Speciation, Structural Refinement, and Distribution of Ti Sites in Titanium Silicalite‐1 From 47/49Ti NMR Crystallography at 28.2 Tesla
Source: Angew Chem Int Ed Engl. 2026 Feb 24;65(15):e24232. doi: 10.1002/anie.202524232 (PMC13053924; doi:10.1002/anie.202524232)
Supplement: Supplementary file 1 — Supporting File 1: Zeolite synthesis, zeolite characterization and catalytic performance, synthesis protocols of molecular library and corresponding solution NMR spectra, single‐crystal XRD structures, solution 47/49Ti NMR spectra of molecular library, Ti K‐edge XANES analysis of molecular library and zeotypes, powder XRD measurements, 17O and 47/49Ti solid‐state NMR measurements and fitting parameters, DFT calculation protocols for periodic geometry optimization and cluster NMR parameters calculations. [file ANIE-65-e24232-s001.pdf]

Supporting Information

Speciation, Structural Refinement, and Distribution of Ti Sites in Titanium Silicalite-1 From $^{47/49}$Ti NMR Crystallography at 28.2 Tesla

Christoph J. Kaul,[1] Jonas Koppe,[2] Lukas Lätsch,[1] Michael Wörle,[1] Sadig Aghazada,[1] Jacob B. Holmes,[3] Christina Wartmann,[4] Mingji Zheng,[1] Albrecht Berkessel,[4] Trees De Baerdemaeker,[5] Andrei-Nicolae Parvulescu,[6] Karsten Seidel,[5] J. Henrique Teles,[5] Alexander V. Yakimov,[1,*] Christophe Copéret[1,*]

1 Department of Chemistry and Applied Biosciences, ETH Zurich, Vladimir-Prelog Weg 2, CH-8093 Zurich, Switzerland
2 Centre de RMN à Très Hauts Champs de Lyon, CNRS/Ecole Normale Supérieure de Lyon/Université Claude Bernard Lyon 1, Villeurbanne, France
3 Institut des Sciences et Ingénierie Chimiques, École Polytechnique Fédérale de Lausanne (EPFL), CH-1015 Lausanne, Switzerland
4 Department of Chemistry and Biochemistry, Organic Chemistry, University of Cologne, Greinstraße 4, 50939 Cologne, Germany
5 BASF SE, Group Research, Carl-Bosch-Straße 38, Ludwigshafen am Rhein, Germany
6 BASF SE, Monomers Division, Carl-Bosch-Straße 38, Ludwigshafen am Rhein, Germany

Corresponding Authors
*Alexander V. Yakimov yakimov@inorg.chem.ethz.ch
*Christophe Copéret ccoperet@ethz.ch

Contents

A Zeolite Synthesis **S1**
A.1 TS-1_1 and TS-$1_{1.5}$ . . . S1
A.2 TS-$1_{1.9}$ . . . S1
A.3 TS-$1_{3.5,\ \text{extra-framework}}$ . . . S1
A.4 TS-$1_{\text{hierarchical}}$ . . . S1
A.5 Silicalite-1 . . . S2

B Zeolite Characterization and Catalytic Performance **S3**
B.1 Surface Area Determination . . . S3
B.2 UV-Vis Spectra . . . S3
B.3 Zeotype Catalyzed Epoxidation of Propylene . . . S4

C Synthesis of Molecular Library and TiO_2 References **S5**
C.1 General Details . . . S5
C.2 TiO_2 References . . . S5
C.3 $[Ti(OTBOS)_4]$, $[Ti(O^iPr)(OTBOS)_3]$, $[Ti(NMe_2)(OTBOS)_3]$, Ti-pentadentate-salan and $[Ti_2O_2(acac)_4]$ S5
C.4 $[TiCl(OTBOS)_3]$ . . . S6

D Single Crystal XRD Structures **S8**
D.1 General Details . . . S8
D.2 $[Ti(OTBOS)_4]$, $[Ti(O^iPr)(OTBOS)_3]$, Ti-pentadentate-salan and $[Ti_2O_2(acac)_4]$ . . . S8
D.3 $[TiCl(OTBOS)_3]$ . . . S9

E Solution $^{47/49}$Ti NMR of Molecular Library **S10**

F Ti K-edge XANES Analysis of Molecular Library and Zeotypes **S11**
F.1 General Details . . . S11
F.2 Molecular Library . . . S11
F.3 Dehydrated and Hydrated Ti-Zeotypes . . . S12

G Powder XRD Measurements **S13**
G.1 General Details . . . S13
G.2 Ti-Zeotypes . . . S13

H Solid-State NMR Measurements **S15**
H.1 General Details . . . S15
H.2 $^{47/49}$Ti NMR Measurements . . . S16
H.2.1 Experimental Details of $^{47/49}$Ti NMR Measurements . . . S16
H.2.2 Additional $^{47/49}$Ti NMR Spectra . . . S19
H.3 ^{17}O NMR Measurements . . . S22
H.3.1 ^{17}O Labeling of Silicalite-1 and TS-1 . . . S22
H.3.2 Experimental Details of ^{17}O 1D NMR Measurements . . . S22
H.3.3 Additional ^{17}O NMR Spectra . . . S24
H.3.4 2D ^{17}O Magic-Angle-Turning (MAT) . . . S27
H.4 Fitting of Solid State NMR Spectra . . . S29
H.5 Extended Czjzek Simulations . . . S34
H.6 Spin Counting . . . S35
H.7 Discussion of Extra-framework TiO_2 in high weight loading classical TS-1 . . . S36

I DFT Calculations **S37**
I.1 General Details . . . S37
I.2 Periodic Geometry Optimizations . . . S37
I.2.1 Molecules . . . S37
I.2.2 Silicalite-1 and TS-1 Framework (T-Site) Model Generation . . . S37
I.2.3 TS-1 Framework Associated Site Model Generation . . . S37
I.3 ^{49}Ti NMR Parameter Calculations . . . S38
I.3.1 ^{49}Ti NMR Parameter Benchmark and Molecular Model Systems . . . S38

I.3.2 ^{49}Ti NMR Signatures of Ti-zeotypes . . . S43
I.3.3 ^{49}Ti NMR Parameters Dependency of $Ti(OSiF_3)_4$ Cluster Model on Ti-O Bond Length, O-Ti-O and Ti-O-Si Bond Angle . . . S45
I.4 ^{17}O NMR Parameter Calculations . . . S47
I.4.1 Benchmark and Molecular Model Systems . . . S47
I.4.2 ^{17}O NMR Signatures of Ti-Zeotypes . . . S51
I.4.3 ^{17}O NMR Parameters Dependency of $Ti(OSiF_3)_4$ Cluster Model on Ti-O Bond Length, O-Ti-O and Ti-O-Si Bond Angle . . . S52
I.5 Statistical Evaluation . . . S54
I.5.1 Global Uncertainties in the Computational Protocol . . . S54
I.5.2 χ^2_R Statistic . . . S54

References **S56**

A Zeolite Synthesis

A.1 TS-1_1 and TS-$1_{1.5}$

The titanium silicalite-1s (TS-1s) TS-1_1 and TS-$1_{1.5}$ were prepared following synthesis procedures previously reported as Samples 3 and 4 in Reference [1]. Tetraethyl orthosilicate (TEOS) (500 g) was added together with tetraethyl orthotitanate (TEOT) (7.5 g and 11.25 g for the samples with 1.0 wt% (TS-1_1) and 1.5 wt% (TS-$1_{1.5}$), respectively) to a round-bottom flask. 220 g of a 40 wt% solution of tetrapropylammonium hydroxide (TPAOH) were mixed with 300 g of distilled water and then added under stirring to the flask containing the Si and Ti source. The mixture was further kept under stirring for 1 h until the hydrolysis of the silica and titanium sources was finished; the temperature of the mixture was held constant at 60 °C. The ethanol resulting from the hydrolysis of TEOS and TEOT was separated by distillation from the synthesis mixture at 95 °C for 2 h, during which the solution was continuously stirred at 200 rpm. After the distillation, 546 g of distilled water were added and the solution was stirred for another hour at room temperature. Finally, the suspension was transferred to a 2.5-l stainless-steel autoclave equipped with mechanical stirring. The autoclave was heated to 175 °C and kept for 16 h under continuous stirring (200 rpm). After 16 h, the autoclave was cooled to room temperature and distilled water was added to the suspension in a volumetric ratio of 1:1 (the pH of the resulting solution was about 12). The suspension was brought to a pH of around 7 by the addition of a solution of 10-wt% HNO_3. The suspension was then filtered on a Buchner filter and the solid was washed several times with water. The white solid was dried for 4 h at 120 °C and calcined for 5 h at 490 °C under air, using the following calcination program: 60 min to 120 °C, 240 min at 120 °C, 370 min from 120 °C to 490 °C and 300 min at 490 °C. The resulting TS-1_1 had a Si content of 43 wt% and a Ti content of 1.0 wt%. The resulting TS-$1_{1.5}$ had a Si content of 42 wt% and a Ti content of 1.5 wt%.

A.2 TS-$1_{1.9}$

The TS-$1_{1.9}$ zeolite was prepared following the synthesis procedure previously reported as Sample 1 in Reference [1]. 400 kg of TPAOH were added to 550 kg deionized water under stirring. The mixture was stirred for 1 h. In a separate reaction vessel, a mixture of 80 kg of TEOS and 16 kg of TEOT was added to 300 kg of TEOS under stirring. Subsequently, an additional 340 kg of TEOS were added. The TPAOH solution was then added, and the resulting mixture was stirred for another hour. Then, the reaction vessel was heated and the ethanol obtained was separated by distillation. When the internal temperature of the vessel had reached 95 °C, the reaction vessel was cooled. 1143 kg of deionized water were added to the resulting suspension in the vessel, and the mixture was stirred for another hour. Crystallization was performed at 175 °C within 24 h at autogenous pressure. The obtained TS-1 crystals were separated, dried and calcined at a temperature of 500 °C in air. The resulting TS-1 was found to contain 1.9 wt% Ti and 43 wt% Si.

A.3 TS-$1_{\text{3.5, extra-framework}}$

TEOT was added to 485.3 g TEOS in a round bottom flask. 213 g TPAOH (40 wt.%) was diluted with 257 g distilled water and added to the flask containing TEOS and TEOT under stirring. The mixture was stirred for 1 h to complete the TEOS and TEOT hydrolysis during which the temperature increased to 60 °C. The resulting ethanol was distilled at 95 °C for 2h under stirring. After cooling down to 40 °C, 476 g distilled water was added under stirring. 400 g of this gel was placed in a beaker, to which 16.95 g ammonium carbonate was added under stirring. The gel was placed in Teflon lined steel autoclaves, which were closed and placed in a static oven for 24 h at 175 °C. The obtained suspension was filtered on a Buchner filter. After washing the solids several times with water, they were dried for 4h at 120 °C and calcined in air for 5h at 490 °C (heating ramp 2 °C/min). The final TS-1 contained 3.5 wt.% Ti.

A.4 TS-$1_{\text{hierarchical}}$

A TS-1 powder was prepared according to the following recipe: 500 g TEOS and 22.4 g TBOT were loaded into a four-neck flask at room temperature and stirring (200 rpm) was started. Then, 220 g of an aqueous solution comprising 40 wt-% TPAOH and 300 g of deionized water were added. Stirring was continued for 60 min, whereby the temperature of the mixture rose to 60 °C. Crystallization was performed in an autoclave under stirring at 175 °C within 16 h at autogenous pressure. The obtained suspension was worked-up as follows. The suspension was filled into beakers and centrifuged for 60 min with 4000 rpm. The solids were dried in an oven for 10 h at 120 °C and then calcined for 5 h at 500 °C (heating rate 2 °C/min) in air. Next, 675.0 g deionized water and 270 g of an aqueous solution comprising 40 wt-% TPAOH were provided in a beaker. Then, 126 g of the prepared TS-1 powder were added under stirring (200 rpm, anchor stirrer). This mixture was stirred for 60 min, and then

transferred in an autoclave. The mixture was then heated to 170 °C under stirring and stirred for 90 h at 170 °C under autogenous pressure. The obtained suspension was worked-up as follows. The suspension was filled into beakers and centrifuged for 60 min with 4000 rpm. The solids were dried in an oven for 10 h at 120 °C and then calcined for 5 h at 500 °C (heating rate 2 °C/min) in air. The resulting TS-$1_{\mathrm{hierarchical}}$ had a Ti content of 1.9 wt%.

A.5 Silicalite-1

The Silicalite-1 was prepared following the synthesis procedure previously reported as "MFI Zeolite" in Reference [2]. For the gel preparation, 750 g TEOS were filled into a beaker. Then, a solution of 450 g deionized water and 330 g aqueous TPAOH (40 wt% in water) was added under stirring (200 rpm). The mixture was hydrolyzed at room temperature for 60 min during which the temperature rose to 55 °C. Afterwards the ethanol was distilled off until the sump reached a temperature of 95 °C. The synthesis gel was then cooled to 40 °C under stirring and deionized water was added to compensate the mass lost during distillation. The synthesis gel was then transferred into an autoclave. The synthesis gel was heated under stirring in the autoclave to a temperature of 175 °C and stirred at said temperature for 16 h under autogenous pressure. The resulting suspension was then worked-up. To this effect, the resulting suspension was diluted with deionized water, wherein the weight ratio of the suspension to de-ionized water was 1:1. Then, about 235 g nitric acid (10 wt% in water) were added. The obtained solids were filtered off and washed three times with deionized water (each time 1000 ml deionized water was used). Subsequently, the solids were dried in an oven in air at 120 °C for 4 h and then calcined in air at 500 °C for 5 h, wherein the heating rate for calcining was 2 °C/min. The resulting Silicalite-1 material had a Si content of 43 wt%, and a total loss of carbon of less than 0.1 wt%.

B Zeolite Characterization and Catalytic Performance

B.1 Surface Area Determination

N_2 adsorption isotherms to determine the BET surface areas were collected on a Micromeritics TriStar device after outgassing the samples for 16 h at 200 °C under vacuum. The BET specific surface area of the studied Ti-Zeoytpes are displayed in Table S1.

Table S1: The BET specific surface area for the Ti-zeotype samples studied in this work.

Sample	BET specific surface area (m^2/g)
TS-1_1	450[1]
TS-$1_{1.5}$	448[1]
TS-$1_{1.9}$	471[1]
TS-$1_{3.5,\ \text{extra-framework}}$	372
TS-$1_{\text{hierarchical}}$	433
Silicalite-1	435[2]

B.2 UV-Vis Spectra

The UV-Vis measurements were performed with a PerkinElmer Lambda 950 instrument. The measurements were performed at room temperature, in the spectral range 200 to 800 nm with an integration time of 0.2 s and a scan speed of 267 nm/min. Before the measurement the powder sample was pressed into a powder cuvette for solid samples.

The UV-Vis spectrum of TS-1_1 appears to be free of TiO_2 agglomerates, while the spectra of TS-$1_{1.5}$ and TS-$1_{1.9}$ (see Reference [1] for UV-Vis spectrum of TS-$1_{1.9}$) show minimal amounts of extra-framework TiO_2. TS-$1_{3.5,\ \text{extra-framework}}$ and TS-$1_{\text{hierarchical}}$ display large amounts of extra-framework Ti sites.

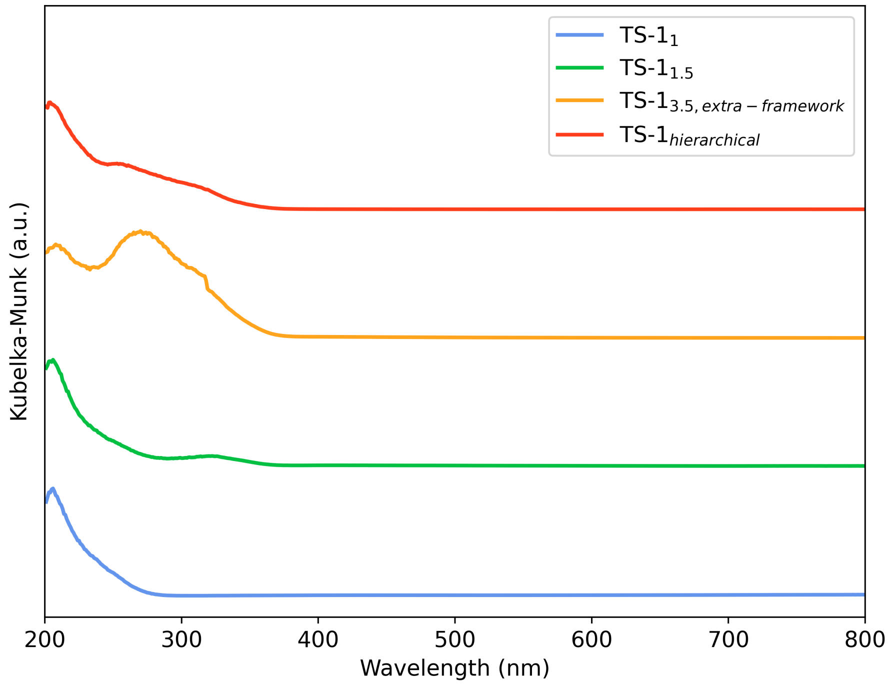

Figure S1: UV–Vis spectra of Ti-zeotypes (TS-1_1 (*blue*), TS-$1_{1.5}$ (*green*), TS-$1_{1.9}$ (see Reference [1] for UV-Vis spectrum), TS-$1_{3.5,\ \text{extra-framework}}$ (*orange*) and TS-$1_{\text{hierarchical}}$ (*red*)) studied in this work.

B.3 Zeotype Catalyzed Epoxidation of Propylene

The TS-1 samples were tested for their catalytic productivity as previously reported in Reference [1]. 0.5 g of TS-1 zeolite powder (half the amount used for the hierarchical TS-1) and 45 ml of methanol were introduced in a 250 ml glass reactor. The reactor was closed and cooled to -25 °C. 20 ml of liquid propylene (at -25 °C, 10 bar) were added to the vessel under vigorous stirring. The reactor was heated to 0 °C. 18 g of H_2O_2 (30-wt% aqueous solution) were subsequently fed by means of a HPLC pump. The reaction was run for 5 h at 0 °C under vigorous stirring. After 5 h, the reaction was stopped, the mixture was depressurized and the reactor was heated to room temperature. The liquid phase was collected and analysed using gas chromatography. The products were quantified by internal standard and by a calibration curve.

The catalytic test evaluating propylene epoxidation performance was carried out in a batch reactor and allows to determine the amount of propylene oxide formed in a given reaction time in the reaction mixture (Table S2). Since all catalysts were tested under the same reaction conditions and reaction time, the resulting propylene oxide concentration does not correlate with the epoxidation rate as a function of the catalyst used. For *classical* TS-1s the yields are found to be similar across different weight loadings (TS-1_1, TS-$1_{1.5}$ and TS-$1_{1.9}$). The catalyst evaluation can be complemented by measuring the pressure drop during the PO test, which is indicative of how fast the propylene is consumed; it is thereby related to the activity of the catalyst. For TS-1_1, TS-$1_{1.5}$ and TS-$1_{1.9}$ the pressure drop rate amounts to 0.0025 bar/min, 0.0042 bar/min, and 0.0082 bar/min, respectively, which indicates that the reaction is faster with higher Ti content. The TS-$1_{\text{hierarchical}}$ catalyst demonstrates markedly higher activity per Ti and larger yields (see Table S2), despite similar Ti content. In contrast TS-$1_{3.5,\ \text{extra-framework}}$ displays both reduced yield and a smaller pressure drop rate pointing towards the reduced activity due to the presence of extra-framework TiO_2.

Table S2: Catalytic productivity and pressure drop rate obtained for the Ti-zeotype samples studied in this work. The propylene oxide (PO) in the product solution was determined; the theoretical maximum concentration is approximately 15 wt-%.

Sample	Ti (wt%)	catalyst amount (g)	PO concentration (wt%)	pressure drop rate (bar/min)
TS-1_1	1.0	0.5	7.7[1]	0.0025
TS-$1_{1.5}$	1.5	0.5	8.2[1]	0.0042
TS-$1_{1.9}$	1.9	0.5	8.0[1]	0.0082
TS-$1_{3.5,\ \text{extra-framework}}$	3.5	0.5	4.8	0.0028
TS-$1_{\text{hierarchical}}$	1.9	0.25	10.4	0.0081

C Synthesis of Molecular Library and TiO_2 References

C.1 General Details

All manipulations involving air- and moisture-sensitive compounds were carried out under an argon atmosphere using standard Schlenk technique or an argon-filled M-Braun glovebox. Toluene and *n*-pentane were dried by passage through double M-Braun SPS alumina solvent purification columns, and further degassed by three freeze-pump-thaw cycles. Tetrahydrofuran (THF) and benzene-d6 were dried over Na/benzophenone and distilled before use. All solvents were stored over activated 4 Å molecular sieves prior to use. Celite® and 4 Å molecular sieves were activated overnight at 320 °C under high vacuum. $TiCl_4$ (99.9%), $Ti(O^iPr)_4$ (99.999%), $Ti(NMe_2)_4$ (99.999%), $(^tBuO)_3SiOH$ (≥99.999%) and NaH were purchased from Sigma-Aldrich and used as received. $NaOSi(OtBu)_3$[3] and $TiCl_4(thf)_2$[4] were synthesized via adapted literature procedures. Elemental analysis of molecular samples was provided by the in-house Molecular and Biomolecular Analysis Service (MoBiAS) of ETH Zürich. Solution ^1H and ^{13}C NMR spectra were recorded in Teflon J. Young valve-sealed NMR tubes on a Bruker 300 MHz DRX spectrometer at room temperature and the chemical shifts are referred in ppm referenced to the solvent. Spectra were processed, analyzed, and plotted using the MestreNova software package. Signal multiplicity is reported as s=singlet, d=doublet, t=triplet, hept=heptet, m=multiplet, or combinations thereof.

C.2 TiO_2 References

Anatase (Titanium(IV) oxide, anatase, nanopowder, < 25 nm particle size, 99.7 % trace metals basis, LOT# MKBP2332V) and Rutile (Titanium(IV) oxide, rutile, nanopowder, < 100 nm particle size, 99.5 % trace metals basis, LOT# BCBF1496V) were purchased from Sigma-Aldrich and used without further purification.

C.3 $[Ti(OTBOS)_4]$, $[Ti(O^iPr)(OTBOS)_3]$, $[Ti(NMe_2)(OTBOS)_3]$, Ti-pentadentate-salan and $[Ti_2O_2(acac)_4]$

$[Ti(OTBOS)_4]$[5] (**1**), $[Ti(O^iPr)(OTBOS)_3]$[6] (**2**), $[Ti(NMe_2)(OTBOS)_3]$[7] (**3**) and Ti-pentadentate-salan[8] (**5**) were synthesized according to literature procedures.

$[Ti_2O_2(acac)_4]$ (**6**) (min. 95%) was purchased from Strem Chemicals, Inc. (STR) and was used as received.

C.4 [TiCl(OTBOS)$_3$]

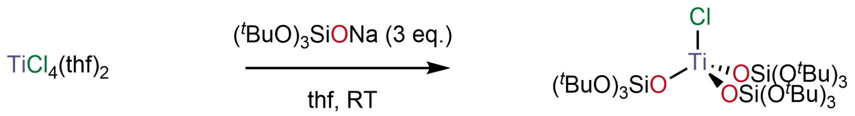

$TiCl_4(thf)_2$ (500 mg, 1.50 mmol, 1.0 equiv.) and $NaOSi(OtBu)_3$ (1284 mg, 4.49 mmol, 3.0 equiv.) were dissolved separately in 20 ml of THF each. The $TiCl_4(thf)_2$ was cooled with an ice bath, and while cooled, the $NaOSi(OtBu)_3$ solution was slowly added. The reaction mixture was allowed to warm to room temperature and stirred for 19 h. All volatiles were removed under reduced pressure to give a white solid. The reaction mixture was extracted with pentane and filtered through celite. The concentration of the solution and the cooling to -30 ° C yielded colorless platelets of [TiCl(OTBOS)$_3$] (922 mg, 1.06 mmol, yield = 70 %). Recrystallization in HMDSO afforded single crystals suitable for XRD diffraction.

^{1}H NMR (300 MHz, benzene−d$_6$) δ (ppm) = 1.51 (s, 81H, -OSi(O^{t}Bu)$_3$). **^{13}C NMR** (75 MHz, benzene−d$_6$) δ (ppm) = 32.0 (−C(CH$_3$)$_3$), 73.4 (−C(CH$_3$)$_3$). **Elemental analysis** calcd (%) for $C_{36}H_{81}ClO_{12}Si_3Ti$: C 49.50, H 9.35; found: C 49.50, H 9.62.

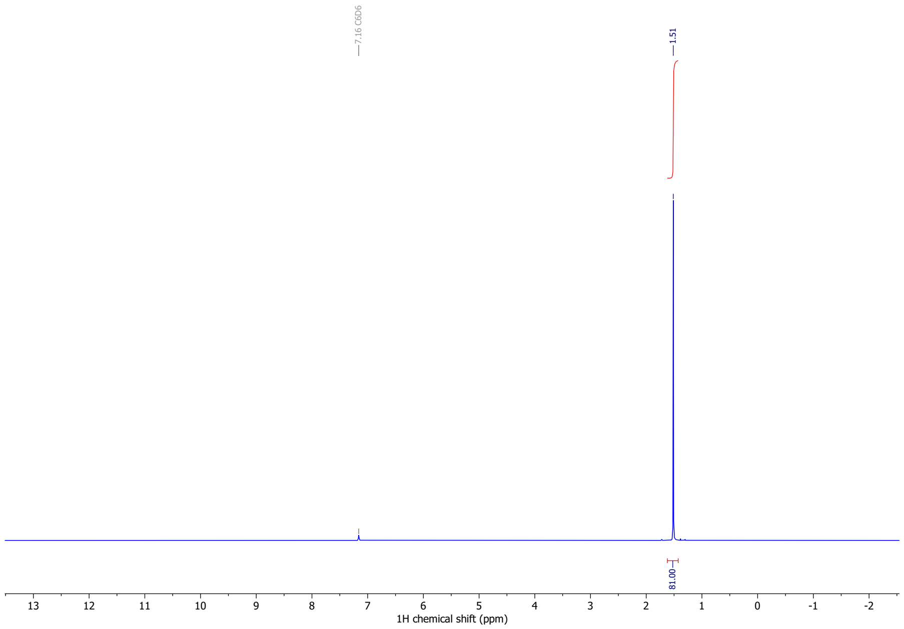

Figure S2: ^{1}H NMR spectrum of [TiCl(OTBOS)$_3$] in C_6D_6 at room temperature.

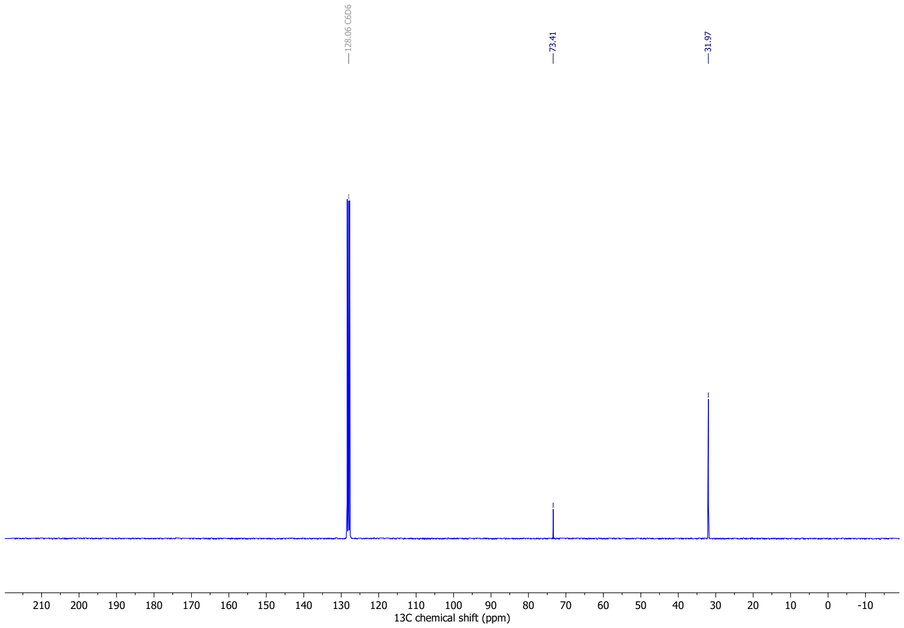

Figure S3: ^{13}C NMR spectrum of [TiCl(OTBOS)$_3$] in C_6D_6 at room temperature.

D Single Crystal XRD Structures

D.1 General Details

For single crystal X-ray diffraction analysis, suitable crystals were placed onto MiTeGen loop pins coated in paratone oil and mounted under a flow of nitrogen at 100 K on a Rigaku Synergy-S diffractometer with a Rigaku HyPix-6000HE area detector using Cu Kα radiation. Using Olex2,[9] the structures were solved with SHELXT[10] structure solution program and refined using SHELXL.[11] All non-hydrogen atoms were refined with anisotropic displacement parameters. Hydrogen atoms were placed in calculated positions and refined as riding atoms.

D.2 [Ti(OTBOS)$_4$], [Ti(O^{i}Pr)(OTBOS)$_3$], Ti-pentadentate-salan and [Ti$_2$O$_2$(acac)$_4$]

The single crystal XRD data for the previously reported complexes in the molecular library, can be found in the Cambridge Crystallographic Data Centre: [Ti(OTBOS)$_4$] (CCDC no. 2205478),[5] [Ti(O^{i}Pr)(OTBOS)$_3$] (CCDC no. 1882540),[12] [Ti(NMe$_2$)(OTBOS)$_3$] (CCDC no. 2361357),[7] Ti-pentadentate-salan (CCDC no. 2306102),[8] and [Ti$_2$O$_2$(acac)$_4$] (CCDC no. 177977).[13]

D.3 [TiCl(OTBOS)$_3$]

The crystal structure of [TiCl(OTBOS)$_3$] was measured and deposited at the Cambridge Crystallographic Data Centre under the number CCDC no. 2482638.

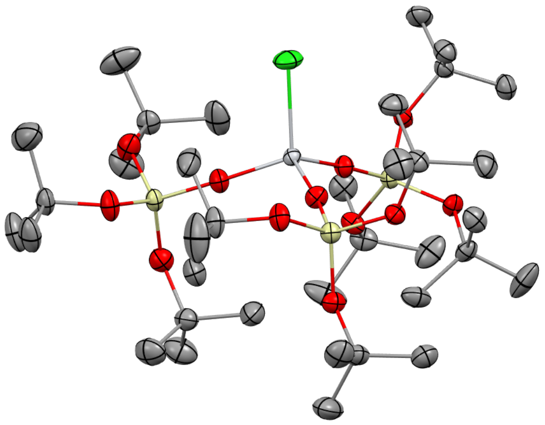

Figure S4: ORTEP drawing of [TiCl(OTBOS)$_3$] with ellipsoids at the 50% probability level. Hydrogen atoms were omitted for clarity.

Table S3: Crystal Data and Data Collection Parameters of [TiCl(OTBOS)$_3$].

Structure	[TiCl(OTBOS)$_3$]
CCDC Deposition Number	2482638
Empirical formula	$C_{36}H_{81}ClO_{12}Si_3Ti$
Formula weight (g/mol)	873.62
Crystal system	monoclinic
Space group	$P2_1/c$
a/Å	21.33605(10)
b/Å	9.07989(6)
c/Å	26.28462(12)
α/°	90
β/°	96.4527(4)
γ/°	90
Volume/Å^3	5059.83(5)
Z	4
ρ_{calc}/g cm^{-3}	1.147
μ[Cu-Kα]/mm^{-1}	3.003
F(000)	1896.0
Temperature/K	100.00(10)
Crystal size/mm^3	0.2 × 0.2 × 0.2
Radiation	Cu Kα (λ = 1.54184)
2Θ range for data collection/°	6.768 to 160.658
Reflections collected	124868
Independent reflections	10889 [R_{int} = 0.0338, R_{sigma} = 0.0143]
Data/restraints/parameters	10889/3/536
Goodness-of-fit on F^2	1.037
$R1$[a]($I \geq 2.0\sigma(I)$)	0.0335
$wR2$[b]($I \geq 2.0\sigma(I)$)	0.0869
$R1$[a](all data)	0.0340
$wR2$[b](all data)	0.0873
Largest diff. peak/hole/ e Å^{-3}	0.93/-0.38

[a] $R1 = \frac{\sum ||F_{obs}|-|F_{calc}||}{\sum |F_{obs}|}$

[b] $wR2 = \sqrt{\frac{\sum w(F_{obs}^2 - F_{calc}^2)^2}{\sum w(F_{obs}^2)^2}}$

E Solution $^{47/49}$Ti NMR of Molecular Library

$^{47/49}$Ti solution NMR spectra were measured on a 500 MHz (11.7 T) Bruker Avance II HD at 298 K using one-pulse experiments (see Figure S5) and compared with the isotropic ^{49}Ti chemical shift parameters extracted from $^{47/49}$Ti solid-state NMR experiments (see Table S18) and the calculated isotropic ^{49}Ti NMR chemical shifts (see Table S26). This highlights that the error between predicted and experimental solution isotropic chemical shift is smaller for the molecular references ([Ti(OTBOS)$_4$], [Ti(O^{i}Pr)(OTBOS)$_3$], [Ti(NMe$_2$)(OTBOS)$_3$]) than between the solid-state NMR measurements and the predicted values (see Section I.5.1). However, for consistency with the experimental data acquired for the TS-1 catalysts the error determination has been conducted based on the solid-state NMR data. For the other molecular references, no $^{47/49}$Ti solution NMR signal was detected.

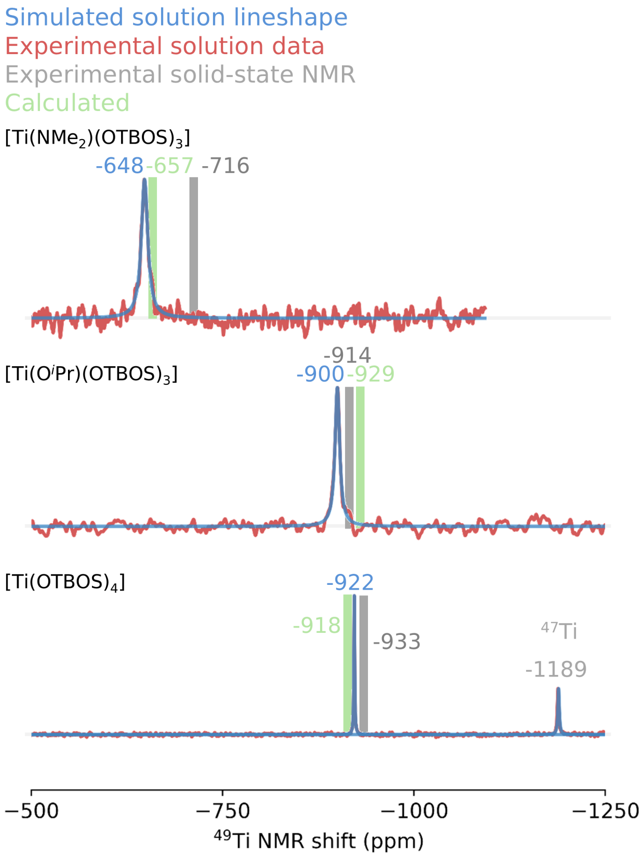

Figure S5: $^{47/49}$Ti NMR spectra of [Ti(OTBOS)$_4$], [Ti(O^{i}Pr)(OTBOS)$_3$], and [Ti(NMe$_2$)(OTBOS)$_3$] in C_6D_6 at room temperature (experimental solution NMR data in *red*, simulated lineshapes in *blue*). The solution NMR data were compared with the isotropic chemical shifts extracted from the solid-state NMR measurements (*grey*) and the predicted isotropic chemical shifts (*green*).

F Ti K-edge XANES Analysis of Molecular Library and Zeotypes

F.1 General Details

Ti K-edge XANES (X-ray Absorption Near Edge Structure) spectra were recorded at the European Synchrotron Radiation Facility (Grenoble, France). The X-ray beam was monochromatized using a liquid nitrogen cooled Si(111) monochromator. A calibration of the energy was performed using Ti reference foil (Ti K-edge position at 4966 eV). The XAS K-edge measurements were carried out with a fluorescent scheme of detection using a silicon drift diode with associated digital electronics. Demeter software (0.9.24) from the Ifeffit software package (Version 1.2.11) was used for the XAS data-analysis.[14] The hydrated TS-1 sample (hydrated at ambient air, relative humidity ca. 50%) was measured as pellet, whereas the dehydrated TS-1 samples (dehydrated for 10 h at ca. 400 °C under high vacuum, heating ramp 200 °C/h) and the molecular analogues ([Ti(OTBOS)$_4$], [Ti(O^{i}Pr)(OTBOS)$_3$], [Ti(NMe$_2$)(OTBOS)$_3$], [TiCl(OTBOS)$_3$], Ti-pentadentate-salan and [Ti$_2$O$_2$(acac)$_4$]) were measured in sealed quartz capillaries.

F.2 Molecular Library

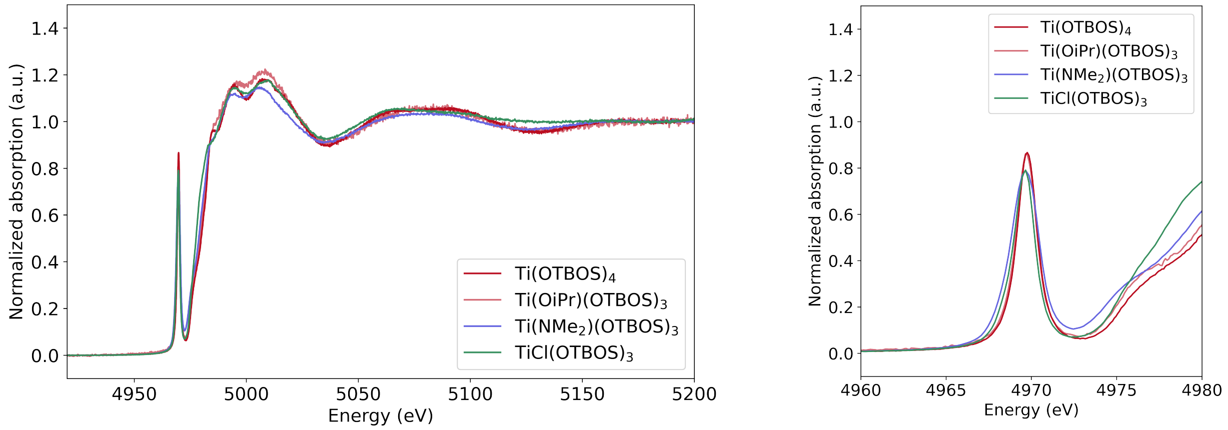

Figure S6: Ti K-edge XANES of tetrahedral complexes in the molecular library: [Ti(OTBOS)$_4$][5] (*red*), [Ti(O^{i}Pr)(OTBOS)$_3$][5] (*light red*), [Ti(NMe$_2$)(OTBOS)$_3$] (*blue*) and [TiCl(OTBOS)$_3$] (*green*).

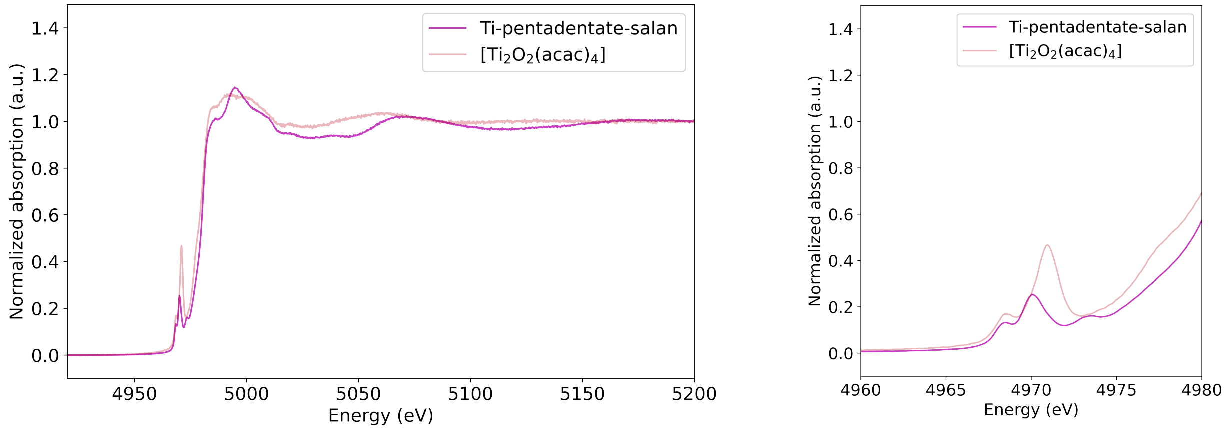

Figure S7: Ti K-edge XANES of octahedral complexes in the molecular library: Ti-pentadentate-salan (*pink*) and [Ti$_2$O$_2$(acac)$_4$][15] (*light red*).

F.3 Dehydrated and Hydrated Ti-Zeotypes

The dehydrated TS-$1_{1.5}$ sample shows a slightly reduced height of the main pre-edge peak of 0.81 compared to the [Ti(OTBOS)$_4$] (0.87) reference compound, which we attributed to the presence of minimal amounts octahedral Ti species. The hydrated TS-$1_{1.5}$ sample shows a to ca. 50 % reduced pre-edge feature (0.45) compared to the dehydrated sample, indicating water coordination to Ti (higher coordination number). In contrast to the dehydrated TS-$1_{1.5}$ sample, the dehydrated TS-$1_{\mathrm{hierarchical}}$ sample exhibits large amounts of extra-framework TiO_2 since the main pre-edge peak height is reduced to 0.56.

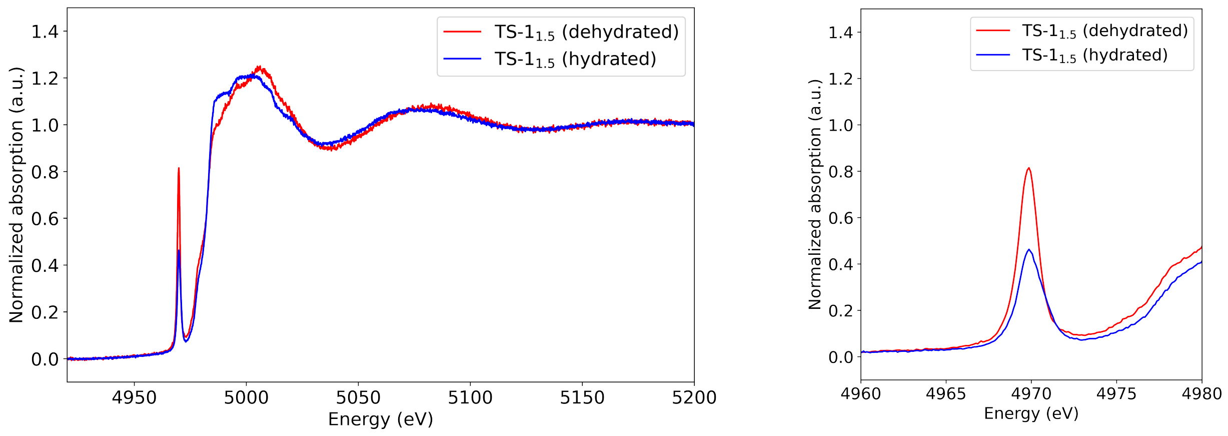

Figure S8: Ti K-edge XANES of hydrated (*blue*)[5] and dehydrated (*red*) TS-$1_{1.5}$.[5]

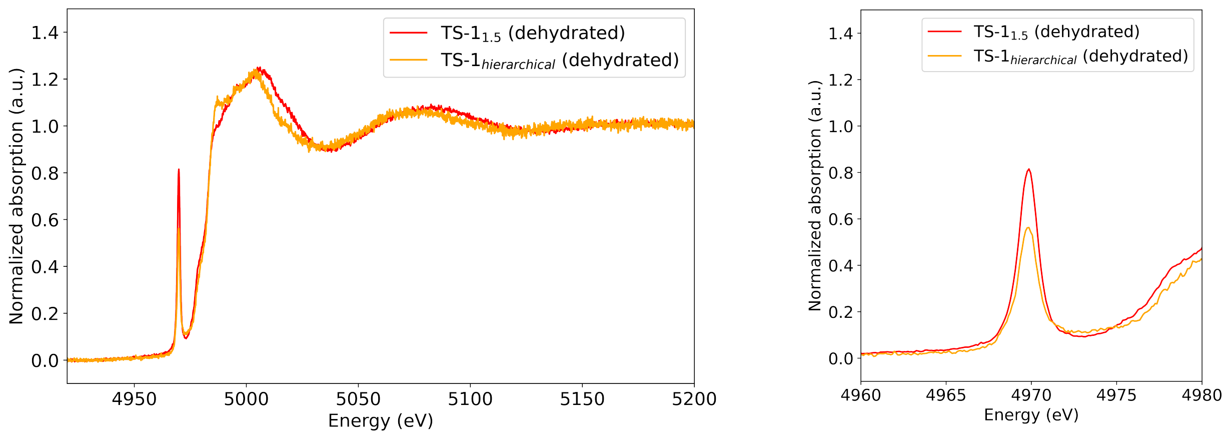

Figure S9: Ti K-edge XANES of dehydrated TS-$1_{1.5}$[5] (*red*) and TS-$1_{\mathrm{hierarchical}}$ (*orange*).

G Powder XRD Measurements

G.1 General Details

Powder diffraction experiments at 296 K were performed on a Stoe STADIP diffractometer (Cu-Kα1-radiation, λ = 1.540510 Å, 2-50° 2θ, curved Ge-monochromator, Dectris Mythen 1K silicon-strip detector). The lattice constants were refined with the program GSAS II,[16] using the values given in the literature for the parent Silicalite-1 framework (Inorganic Crystal Structure Database, Silicalite-1[17] (ICSD Collection Code: 280364, ICSD release 2025.1):[18] *Pnma*, a = 20.05109(10) Å, b = 19.87569(10) Å, c = 13.36823(9) Å) as starting values. The reflection profile employed in the fit has been used as obtained from an evaluation of the powder pattern of a Si-standard (NIST 640c)[19] while only the asymmetry parameter SH/L and the crystallite-size related reflection broadening were allowed to refine further. The samples were prepared in glass capillaries with a diameter of 0.7 mm, which were sealed in the case of the dehydrated TS-1 samples (dehydrated for 10 h at ca. 400 °C under high vacuum, heating ramp 200 °C/h).

G.2 Ti-Zeotypes

Table S4: Lattice constants of classical TS-1 samples with minimal amount of TiO_2 (TS-1_1, TS-$1_{1.5}$ and TS-$1_{1.9}$) obtained from pXRD as described in Section G.1. From experience it is known that the standard uncertainties of the lattice constants as obtained from pXRD are often underestimated.

Sample	Temp. (K)	Space group	Lattice constants a (Å)	b (Å)	c (Å)	Volume (Å^3)
TS-1_1	296	*Pnma*	20.0760(4)	19.8987(4)	13.3812(3)	5345.6(2)
TS-$1_{1.5}$	296	*Pnma*	20.0834(6)	19.9050(7)	13.3847(5)	5350.7(3)
TS-$1_{1.9}$	296	*Pnma*	20.0922(6)	19.9158(6)	13.3960(5)	5360.4(2)

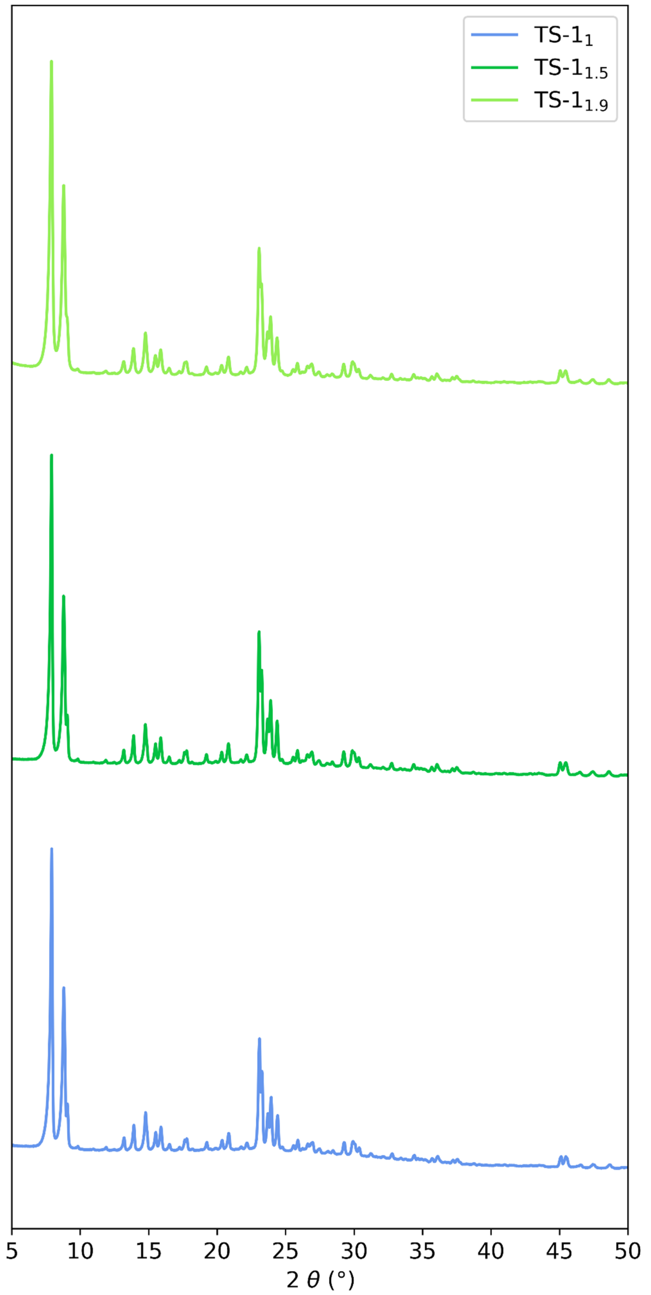

Figure S10: Powder X-ray diffractograms of the dehydrated Ti-zeotypes measured at 296 K (TS-1_1 (*blue*), TS-$1_{1.5}$ (*dark green*), and TS-$1_{1.9}$ (*light green*).

H Solid-State NMR Measurements

H.1 General Details

Air-and moisture-sensitive samples (dehydrated TS-1 samples (dehydrated for 10 h at ca. 400 °C under high vacuum, heating ramp 200 °C/h), [(Ti(OTBOS)$_4$], [Ti(O^{i}Pr)(OTBOS)$_3$], [Ti(NMe$_2$)(OTBOS)$_3$], [TiCl(OTBOS)$_3$]) were packed in an Ar-filled glovebox (< 0.5 ppm O_2, < 0.5 ppm H_2O) and transferred to the NMR spectrometer in a tightly sealed screw-cap vial. The solid-state $^{47/49}$Ti NMR spectra were acquired on Bruker 600 MHz (14.1 T) and 1200 MHz (28.2 T) NMR spectrometers equipped with 3.2 mm double-resonance Magic Angle Spinning (MAS) probes. The solid-state ^{17}O NMR spectra were acquired on Bruker 400 MHz (9.4 T) and 700 MHz (16.4 T) NMR spectrometers equipped with 3.2 mm double-resonance Magic Angle Spinning (MAS) probes. The samples were packed in 3.2 mm thin-wall ZrO_2 rotors closed with Vespel drive caps. The dehydrated TS-1 samples were measured with SiO_2 plugs. All molecular samples were measured at low temperature $\sim$ 100 K. For these low temperature experiments three layers of Sharpie^{TM} marker were applied, to compensate for plastic contraction at $\sim$ 100 K. The acquired NMR spectra were fitted using DMFit software (see Section H.4 for more details).[20]

H.2 $^{47/49}$Ti NMR Measurements

^{49}Ti NMR chemical shifts were referenced with respect to $SrTiO_3$ measured at room temperature, for which the most deshielded peak corresponding to the ^{49}Ti isotope, was assigned to -843 ppm.[21]

H.2.1 Experimental Details of $^{47/49}$Ti NMR Measurements

The $^{47/49}$Ti static NMR spectrum of [Ti(OTBOS)$_4$] was recorded with a Hahn-echo pulse sequence see Table S5 for measurement details.

Table S5: Measurement details of static $^{47/49}$Ti Hahn-echo NMR (14.1 T) experiment of [Ti(OTBOS)$_4$] (**1**) displayed in Figure 5.

	**1**
Larmor frequency (MHz)	33.8015
MAS (kHz)	0
Recycle delay (s)	0.2
Dwell time (μs)	0.5
Number of scans	381264
Ring-down delay τ (μs)	10
90 ° Pulse length P_1 (μs)	3.5
180 ° Pulse length P_2 (μs)	7.0
ν_{RF}^{a} (kHz)	18.1
Decoupling (kHz)	28 (cw)

^{a} The ν_{RF} was calibrated using the Bloch-Siegert shift method[22] at low temperature (100 K).

Besides the spectrum of [Ti(OTBOS)$_4$], all $^{47/49}$Ti NMR spectra in this work were recorded with (W)CPMG-echo train acquisition.[23,24] Dfs pulses[25,26] were used for signal enhancement. This method was employed for the acquisition of spectra of the Ti complex [Ti(O^{i}Pr)(OTBOS)$_3$] (pulse length = 1000 μs, $\Delta\nu$= ± 150 to ± 1000 kHz, ν_{rf} = 6.7 kHz), anatase (pulse length = 500 μs, $\Delta\nu$= ± 100 to ± 500 kHz, ν_{rf} = 18.1 kHz) and all TS-1 samples (pulse length = 500 μs, $\Delta\nu$= ± 100 to ± 500 kHz, ν_{rf} = 18.1 kHz). For static measurements wideband uniform-rate smooth truncation (WURST)[27] pulses were used for the direct excitation. For all (W)CPMG measurements, the envelope spectra were obtained by coadding the individual echoes of the (W)CPMG-echo train in the time domain. The resulting full echoes were then apodised using a Hamming window function with α = 0.54. When the full $^{47/49}$Ti NMR signatures were reconstructed from two subspectra, the skyline projection method was used. The experimental parameters are summarized in Tables S6 - S11. The spikelet spectra were obtained by Fourier transformation of the entire CPMG echo train with the Hamming window applied to all echos individually, followed by a magnitude calculation. In general, signals, which are boarder than 250 kHz, were acquired with offsets separated by less than $\Delta\nu$= 135000 kHz.

Table S6: Measurement details of static $^{47/49}$Ti WCPMG NMR (14.1 T) experiments of the molecular compounds [Ti(O^{i}Pr)(OTBOS)$_3$] (**2**), [Ti(NMe$_2$)(OTBOS)$_3$] (**3**), [TiCl(OTBOS)$_3$] (**4**), Ti-pentadentate-salan (**5**), and [Ti_2O_2(acac)$_4$] (**6**) displayed in Figure 5.

	**2**	**3**	**4**	**5**	**6**
Larmor frequency (MHz)	33.8015	33.8015	(33.6662, 33.8015)	33.7677	(33.6688, 33.7677, 33.8665)
MAS (kHz)	0	0	0	0	0
Recycle delay (s)	0.2	0.4	0.4	0.4	0.6
Dwell time (μs)	0.5	0.5	0.5	0.5	0.5
Processed echoes	5	49	99	69	289
Number of scans	1503328	533680	(502344, 619928)	638520	(102400, 102400, 102400)
Echo delay τ1 (μs)	800	60	60	60	60
Ring-down delay τ2 (μs)	51	51	51	51	51
WURST-pulse length (μs)	10	10	10	10	10
Sweep width (kHz)	1000	1000	1000	1000	1000
WURST-pulse shape parameter	10	10	10	10	10
$\nu_{rf,\ max}^{opt,\ static\,a}$ (kHz)	20.6	20.6	20.6	20.6	20.6
Decoupling (kHz)	28 (cw)	28 (cw)	28 (cw)	28 (cw)	28 (cw)

^{a} The ν_{RF} was calibrated using the Bloch-Siegert shift method[22] at low temperature (100 K).

Table S7: Measurement details of $^{47/49}$Ti CPMG-MAS NMR (28.2 T) experiments of the dehydrated TS-1 samples displayed in Figure 2, 3, 4 and S20.

	TS-1_1	TS-$1_{1.5}$	TS-$1_{1.9}$	TS-$1_{\text{hierarchical}}$	TS-$1_{3.5,\ \text{extra-framework}}$
Larmor frequency (MHz)	67.6329	67.6391	67.6329	67.6391	67.6329
MAS (kHz)	20	20	20	20	20
Recycle delay (s)	0.25	0.25	0.25	0.25	0.25
Dwell time (μs)	0.5	0.5	0.5	0.5	0.5
Processed echoes	25	25	25	25	25
Number of scans	353136	478400	365648	308272	322160
Echo delay $\tau 1$ (μs)	973.2	973.0	973.2	973.0	973.1
Ring-down delay $\tau 2$ (μs)	11	11	11	11	11
90 ° Pulse length P_1 (μs)	2.4	2.5	2.4	2.5	2.4
180 ° Pulse length P_2 (μs)	4.8	5.0	4.8	5.0	4.9
$\nu_{\text{RF}}{}^{a}$ (kHz)	26.0	24.7	26.0	24.7	25.5

[a] The ν_{RF} was calibrated using neat Ti(O^{i}Pr)$_4$ at room temperature.

Table S8: Measurement details of $^{47/49}$Ti CPMG-MAS NMR (28.2 T) experiments of the hydrated TS-1 samples displayed in Figures 4, S12 and S20.

	TS-1_1	TS-$1_{1.5}$	TS-$1_{1.9}$	TS-$1_{\text{hierarchical}}$	TS-$1_{3.5,\ \text{extra-framework}}$
Larmor frequency (MHz)	67.6329	67.6329	67.6329	67.6329	67.6329
MAS (kHz)	20	20	20	20	20
Recycle delay (s)	0.25	0.25	0.25	0.25	0.25
Dwell time (μs)	0.5	0.5	0.5	0.5	0.5
Processed echoes	25	25	25	25	25
Number of scans	68712	240680	179024	370728	287176
Echo delay $\tau 1$ (μs)	973.4	973.2	973.4	973.4	973.1
Ring-down delay $\tau 2$ (μs)	11	11	11	11	11
90 ° Pulse length P_1 (μs)	2.3	2.4	2.3	2.3	2.4
180 ° Pulse length P_2 (μs)	4.6	4.8	4.6	4.6	4.9
$\nu_{\text{RF}}{}^{a}$ (kHz)	27.1	25.5	27.1	27.1	25.5

[a] The ν_{RF} was calibrated using neat Ti(O^{i}Pr)$_4$ at room temperature.

Table S9: Measurement details of $^{47/49}$Ti CPMG-MAS NMR (28.2 T) experiments of the TiO_2 references displayed in Figures 4, S13 and S20.

	Anatase	Rutile
Larmor frequency (MHz)	67.6391	67.6329
MAS (kHz)	20	20
Recycle delay (s)	0.25	0.25
Dwell time (μs)	0.5	0.5
Processed echoes	25	50
Number of scans	208	14592
Echo delay $\tau 1$ (μs)	973.0	973.4
Ring-down delay $\tau 2$ (μs)	11	11
90 ° Pulse length P_1 (μs)	2.5	2.3
180 ° Pulse length P_2 (μs)	5.0	4.6
$\nu_{\text{RF}}{}^{a}$ (kHz)	24.7	27.4

[a] The ν_{RF} was calibrated using neat Ti(O^{i}Pr)$_4$ at room temperature.

Table S10: Measurement details of static $^{47/49}$Ti WCPMG NMR (28.2 T) experiments of the dehydrated TS-1 samples displayed in Figure S11.

	TS-1_1	TS-$1_{1.5}$	TS-$1_{1.9}$	TS-$1_{\text{hierarchical}}$
Larmor frequency (MHz)	67.6329	67.6391	67.6329	67.6391
MAS (kHz)	0	0	0	0
Recycle delay (s)	0.25	0.25	0.25	0.25
Dwell time (μs)	0.5	0.5	0.5	0.5
Processed echoes	50	50	50	50
Number of scans	703040	816456	777936	521968
Echo delay τ1 (μs)	500	500	500	500
Ring-down delay τ2 (μs)	10	10	10	10
WURST-pulse length (μs)	10	10	10	10
Sweep width (kHz)	600	600	600	600
WURST-pulse shape parameter	10	10	10	10
$\nu_{\text{rf, max}}^{\text{opt, static}}$ [a] (kHz)	15.9	15.9	15.9	15.9

[a] The $\nu_{\text{rf, max}}^{\text{opt, static}}$ was calibrated using neat $Ti(O^iPr)_4$ at room temperature.

Table S11: Measurement details of static $^{47/49}$Ti WCPMG NMR (28.2 T) experiments of the TiO_2 references displayed in Figure S13.

	Anatase	Rutile
Larmor frequency (MHz)	67.6391	67.6329
MAS (kHz)	0	0
Recycle delay (s)	0.25	0.25
Dwell time (μs)	0.5	0.5
Processed echoes	25	100
Number of scans	2552	30736
Echo delay τ1 (μs)	1000	500
Ring-down delay τ2 (μs)	10	10
WURST-pulse length (μs)	10	10
Sweep width (kHz)	600	600
WURST-pulse shape parameter	10	10
$\nu_{\text{rf, max}}^{\text{opt, static}}$ [a] (kHz)	15.9	15.9

[a] The $\nu_{\text{rf, max}}^{\text{opt, static}}$ was calibrated using neat $Ti(O^iPr)_4$ at room temperature.

H.2.2 Additional $^{47/49}$Ti NMR Spectra

H.2.2.1 Static $^{47/49}$Ti NMR Spectra of TS-1 Samples

Static $^{47/49}$Ti WCPMG spectra (298 K, 28.2 T) of the dehydrated TS-1 samples studied in this work. The skyline projections were obtained with the same procedure as described in Section H.2.1

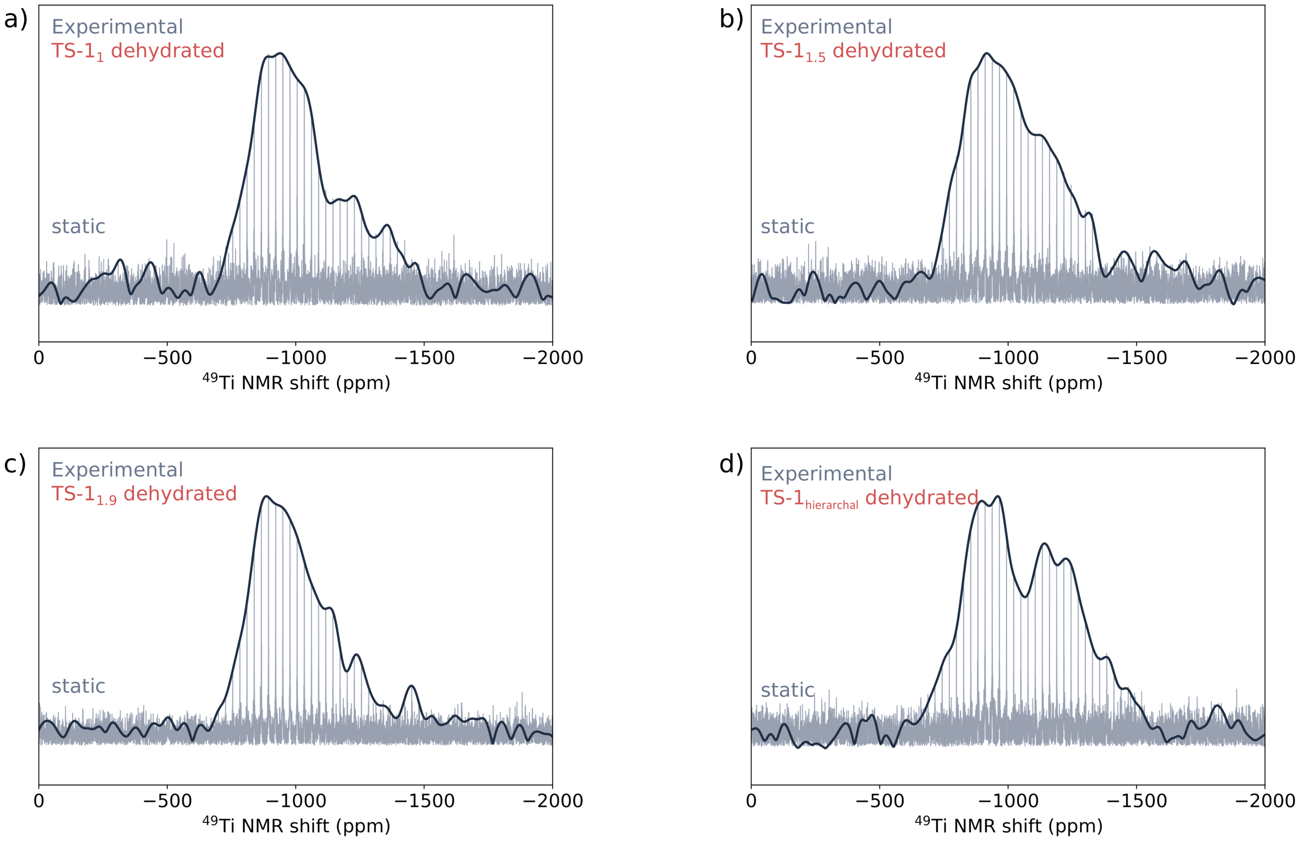

Figure S11: $^{47/49}$Ti NMR spectra of dehydrated TS-1 recorded at 298 K and 28.2 T (spikelet spectra in *grey*, envelope spectra in *dark blue*). Spectra referenced to $SrTiO_3$ at room temperature (-843 ppm). (a): TS-1_1, (b): TS-$1_{1.5}$, (c): TS-$1_{1.9}$ and (d): TS-$1_{\mathrm{hierarchical}}$.

H.2.2.2 $^{47/49}$Ti NMR Spectra of Hydrated TS-1 Samples

$^{47/49}$Ti CPMG-MAS spectra (20 kHz, 298 K, 28.2 T) of the hydrated TS-1 samples studied in this work.

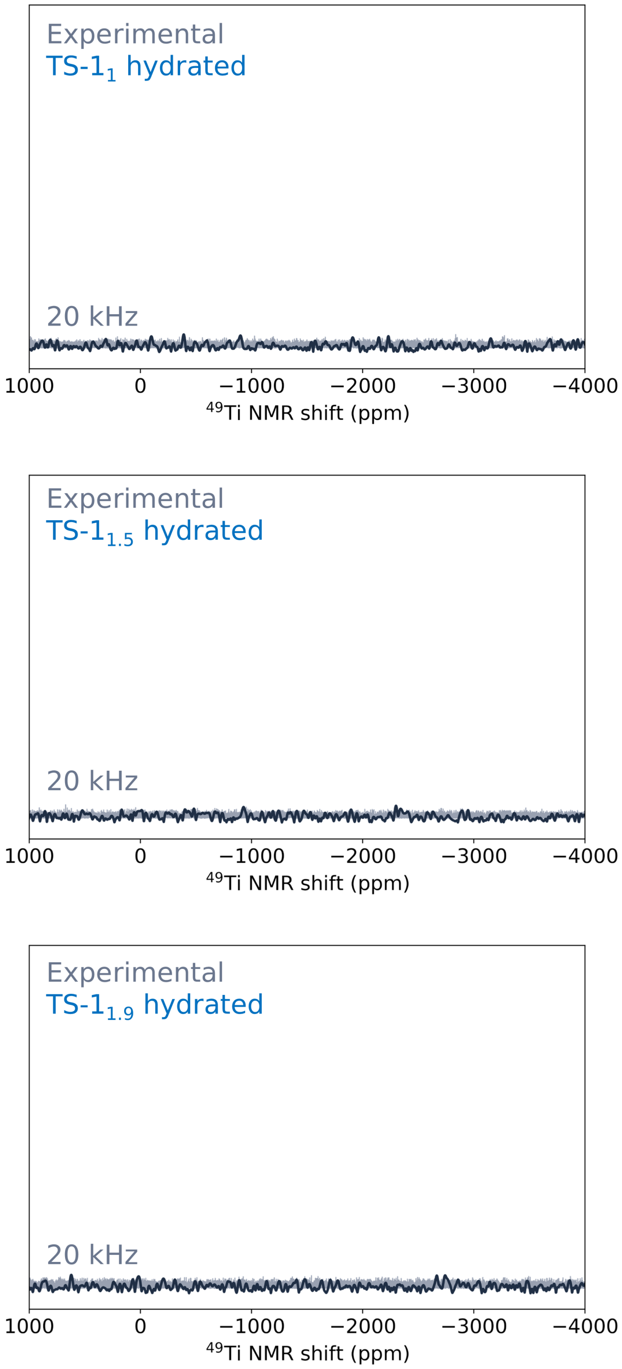

Figure S12: $^{47/49}$Ti NMR spectra of hydrated TS-1 recorded at 298 K and 28.2 T (spikelet spectra in *grey*, envelope spectra in *dark blue*). Spectra referenced to $SrTiO_3$ at room temperature (-843 ppm). (*Left*): TS-1$_1$, (*Middle*): TS-1$_{1.5}$, (*Right*): TS-1$_{1.9}$.

H.2.2.3 $^{47/49}$Ti NMR Spectra of TiO_2 References

Static and MAS (20 kHz) $^{47/49}$Ti (W)CPMG spectra (298 K, 28.2 T) of the TiO_2 references (anatase and rutile) studied in this work.

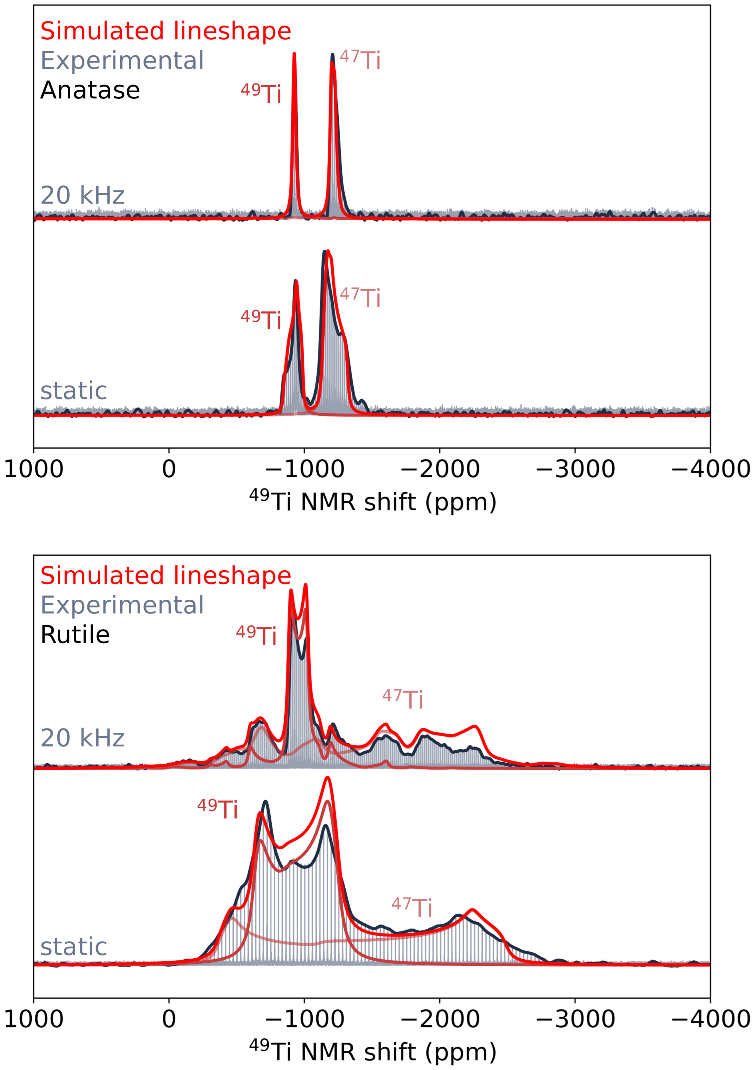

Figure S13: Static and MAS (20 kHz) $^{47/49}$Ti NMR spectra of TiO_2 references recorded at 298 K and 28.2 T (spikelet spectra in *grey*, envelope spectra in *dark blue*). Spectra referenced to $SrTiO_3$ at room temperature (-843 ppm). (*Top*): Anatase, (*Bottom*): Rutile.

H.3 ^{17}O NMR Measurements

All ^{17}O NMR chemical shifts were referenced with respect to H_2O measured at room temperature (0 ppm).

H.3.1 ^{17}O Labeling of Silicalite-1 and TS-1

The as-prepared Silicalite-1 and TS-1 samples (100 mg) were impregnated with 100 μL of $H_2{}^{17}O$ (90%, supplied from Cortecnet; 40% for Silicalite-1 sample) and the resulting slurry was left for at least seven days (TS-1_1 and TS-$1_{1.5}$ eight days; TS-$1_{1.9}$ and TS-$1_{\mathrm{hierarchical}}$ for 9 days). The resulting material was subjected to the same dehydration step as the none labeled material (ca. 400 °C under high vacuum for 10 h, heating ramp 200 °C/h).

H.3.2 Experimental Details of ^{17}O 1D NMR Measurements

Dfs pulses[25,26] were used for signal enhancement for all ^{17}O measurements (pulse length = 500 μs, $\Delta\nu$= ± 100 to ± 500 kHz, ν_{rf} = 12.6 kHz) acquired.
The static ^{17}O WCPMG spectra were processed as described in Section H.2.1. The ^{17}O NMR spectra were obtained by coadding the individual echoes of the WCPMG-echo train in the time domain. The resulting full echoes were then apodised using a Hamming window function with α = 0.54. The experimental parameters are summarized in Tables S12 and S13.

Table S12: Measurement details of static ^{17}O WCPMG NMR (16.4 T) experiments of the dehydrated ^{17}O labeled Silicalite-1 and TS-1 samples (prepared as discussed in Section H.3.1) displayed in Figure 1 and Section H.3.3.

	Silicalite-1	TS-1_1	TS-$1_{1.5}$	TS-$1_{1.9}$	TS-$1_{\mathrm{hierarchical}}$
Larmor frequency (MHz)	94.9318	94.9318	94.9318	94.9318	94.9318
MAS (kHz)	0	0	0	0	0
Recycle delay (s)	10	10	10	10	10
Dwell time (μs)	0.5	0.5	0.5	0.5	0.5
Processed echoes	20	20	20	20	20
Number of scans	16112	5576	1288	3592	1976
Echo delay τ1 (μs)	1000	1000	1000	1000	1000
Ring-down delay τ2 (μs)	11	11	11	11	11
WURST-pulse length (μs)	50	50	50	50	50
Sweep width (kHz)	1000	1000	1000	1000	1000
WURST-pulse shape parameter	80	80	80	80	80
$\nu_{\mathrm{rf,\ max}}^{\mathrm{opt,\ static}\,a}$ (kHz)	12.3	12.3	12.3	12.3	12.3
Decoupling (kHz)	68.9 (spinal 64)	68.9 (spinal 64)	68.9 (spinal 64)	68.9 (spinal 64)	68.9 (spinal 64)

[a] The $\nu_{\mathrm{rf,\ max}}^{\mathrm{opt,\ static}}$ was calibrated on H_2O at room temperature.

Table S13: Measurement details of static ^{17}O WCPMG NMR (9.4 T) experiment of the dehydrated ^{17}O labeled TS-$1_{1.9}$ (prepared as discussed in Section H.3.1) displayed in Figure 1 and Section H.3.3.

	TS-$1_{1.9}$
Larmor frequency (MHz)	54.2679
MAS (kHz)	0
Recycle delay (s)	1
Dwell time (μs)	0.5
Processed echoes	20
Number of scans	44824
Echo delay τ1 (μs)	1000
Ring-down delay τ2 (μs)	11
WURST-pulse length (μs)	50
Sweep width (kHz)	1000
WURST-pulse shape parameter	80
$\nu_{\mathrm{rf,\ max}}^{\mathrm{opt,\ static}\,a}$ (kHz)	12.3
Decoupling (kHz)	86.7 (spinal 64)

[a] The $\nu_{\mathrm{rf,\ max}}^{\mathrm{opt,\ static}}$ was calibrated on H_2O at room temperature.

The ^{17}O MAS NMR spectra were recorded with Hahn-echo pulse sequences and the experimental parameters are summarized in Table S14 and S15.

Table S14: Measurement details of MAS ^{17}O Hahn-echo NMR (16.4 T) experiments of the dehydrated ^{17}O labeled Silicalite-1 and TS-1 samples (prepared as discussed in Section H.3.1) displayed in Figure 1 and Section H.3.3.

	Silicalite-1	$TS\text{-}1_1$	$TS\text{-}1_{1.5}$	$TS\text{-}1_{1.9}$	$TS\text{-}1_{hierarchical}$
Larmor frequency (MHz)	94.9318	94.9318	94.9318	94.9318	94.9318
MAS (kHz)	(5, 8, 14)	(5, 8, 14)	(5, 8, 14)	(5, 8, 14)	(5, 8, 14)
Recycle delay (s)	10	10	10	10	10
Dwell time (μs)	1.25	1.25	1.25	1.25	1.25
Number of scans	(5480, 2616, 5024)	(2872, 4736, 2456)	(1696, 4336, 232)	(4888, 5256, 1464)	(2144, 4376, 2656)
Ring-down delay τ (μs)	6.5	6.5	6.5	6.5	6.5
90 ° Pulse length P_1 (μs)	2.56	2.56	2.56	2.56	2.56
180 ° Pulse length P_2 (μs)	5.12	5.12	5.12	5.12	5.12
$\nu_{RF}{}^{a}$ (kHz)	32.6	32.6	32.6	32.6	32.6
Decoupling (kHz)	68.9 (spinal 64)	68.9 (spinal 64)	68.9 (spinal 64)	68.9 (spinal 64)	68.9 (spinal 64)

[a] The ν_{RF} was calibrated on H_2O at room temperature.

Table S15: Measurement details of MAS ^{17}O Hahn-echo NMR (9.4 T) experiments of the dehydrated ^{17}O labeled $TS\text{-}1_{1.9}$ (prepared as discussed in Section H.3.1) displayed in Section H.3.3.

	$TS\text{-}1_{1.9}$
Larmor frequency (MHz)	54.2679
MAS (kHz)	(5, 8, 14)
Recycle delay (s)	1
Dwell time (μs)	1.25
Number of scans	(16336, 21280, 4608)
Ring-down delay τ (μs)	10
90 ° Pulse length P_1 (μs)	2.0
180 ° Pulse length P_2 (μs)	4.0
$\nu_{RF}{}^{a}$ (kHz)	41.7
Decoupling (kHz)	86.7 (spinal 64)

[a] The ν_{RF} was calibrated on H_2O at room temperature.

H.3.3 Additional ^{17}O NMR Spectra

In addition to the ^{17}O NMR spectra of TS-$1_{1.5}$ shown in the main text Figure 1, we have conducted the same set of experiments for all studied TS-1 samples (TS-1_1, TS-$1_{1.9}$ and TS-$1_{hierarchical}$), as well as a Ti-free Silicalite-1 sample. In order to increase the confidence in the extracted ^{17}O NMR signatures, one TS-1 (TS-$1_{1.9}$) sample was measured at two different fields (9.4 T and 16.4 T).

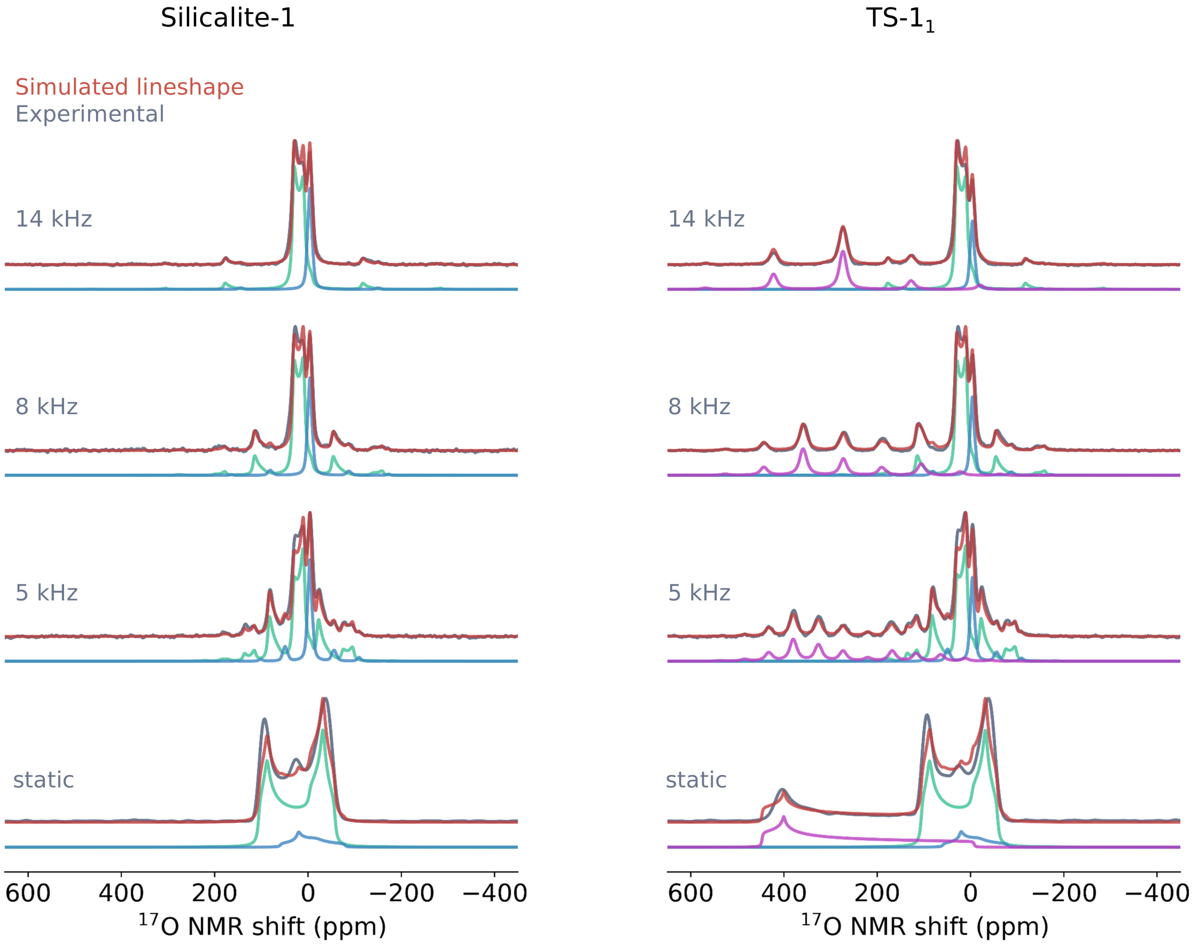

Figure S14: ^{17}O ssNMR (16.4 T) spectra at different spinning speeds of Silicalite-1 and TS-1_1, fitted with the ^{17}O signature of Si-OH (*blue*), Si-O-Si (*green*) and Ti-O-Si (*violet*) (298 K, referenced to H_2O at room temperature (0 ppm)).

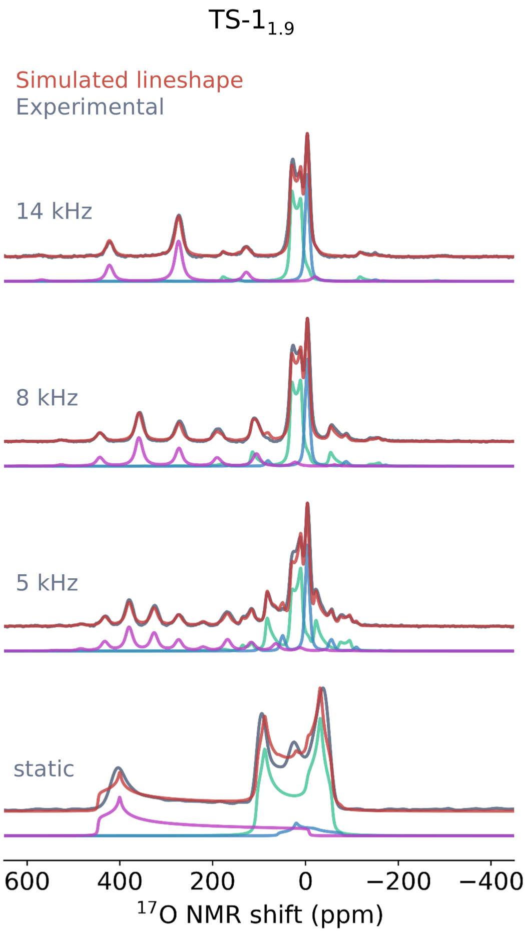

Figure S15: ^{17}O ssNMR (16.4 T) spectra at different spinning speeds of TS-1$_{1.9}$ and TS-1$_{\text{hierarchical}}$, fitted with the ^{17}O signature of Si-OH (*blue*), Si-O-Si (*green*) and Ti-O-Si (*violet*) (298 K, referenced to H_2O at room temperature (0 ppm)).

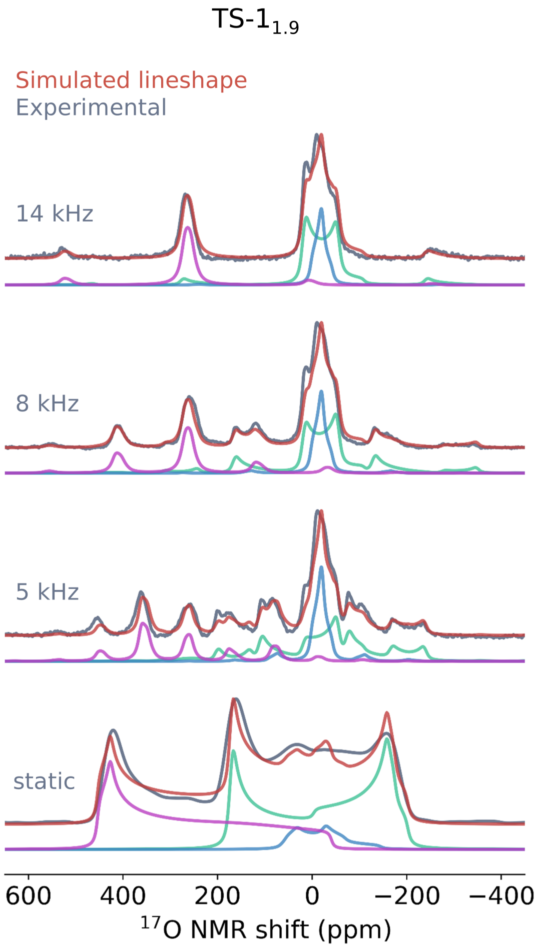

Figure S16: ^{17}O ssNMR (9.4 T) spectra at different spinning speeds of TS-1$_{1.9}$, fitted with the ^{17}O signature of Si-OH (*blue*), Si-O-Si (*green*) and Ti-O-Si (*violet*) (298 K, referenced to H_2O at room temperature (0 ppm)).

H.3.4 2D ^{17}O Magic-Angle-Turning (MAT)

2D ^{17}O magic-angle-turning (MAT) spectra were collected for all zeolites studied in this work and the experimental parameters are summarized in Table S16. The spectra of TS-$1_{1.5}$ is shown in Figure 1, whereas the spectra for the other studied zeolites are shown below. Dfs pulses[25,26] were used for signal enhancement of all 2D ^{17}O measurements (pulse length = 500 μs, $\Delta\nu = \pm$ 100 to $\pm$ 500 kHz, ν_{rf} = 12.6 kHz) acquired.

Table S16: Measurement details of 2D ^{17}O Magic-angle-turning (MAT) (16.4 T) experiments of the dehydrated ^{17}O labeled zeolites (prepared as discussed in Section H.3.1) displayed in Figures S17 and S18.

	Silicalite-1	TS-1_1	TS-$1_{1.5}$	TS-$1_{1.9}$	TS-$1_{\text{hierarchical}}$
Larmor Frequency (MHz)	94.9271	94.9271	94.9271	94.9271	94.9271
MAS rate (kHz)	5	5	5	5	5
Recycle Delay (s)	3.5	3.5	3.5	3.5	3.5
Number of Scans	336	336	336	336	336
Acquisition Time (μs)	1000	1000	1000	1000	1000
Rf-Amplitude (kHz)	32.6	32.6	32.6	32.6	32.6
MAT constant	2/3	2/3	2/3	2/3	2/3
Number of complex increments[a]	128	128	128	128	128
Spectral width in the direct (indirect) dimension (kHz)	400 (80)	400 (80)	400 (80)	400 (80)	400 (80)

[a] echo/antiecho-acquisition scheme was used

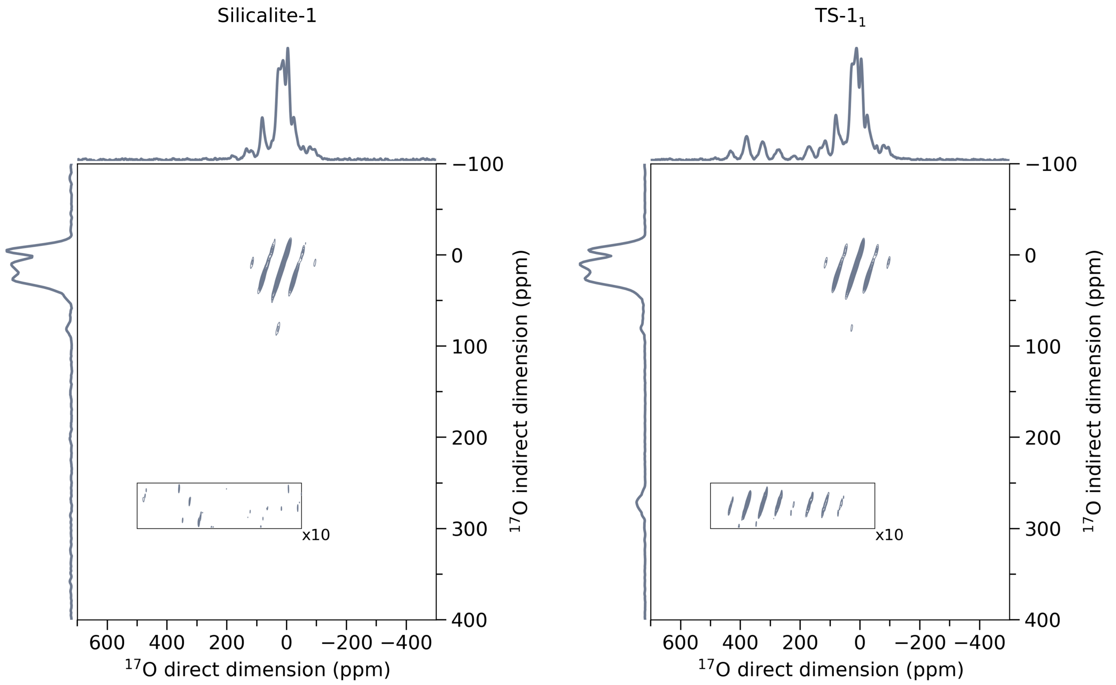

Figure S17: ^{17}O MAT (16.4 T) spectra of Silicalite-1 and TS-1_1 (298 K, referenced to H_2O at room temperature (0 ppm)).

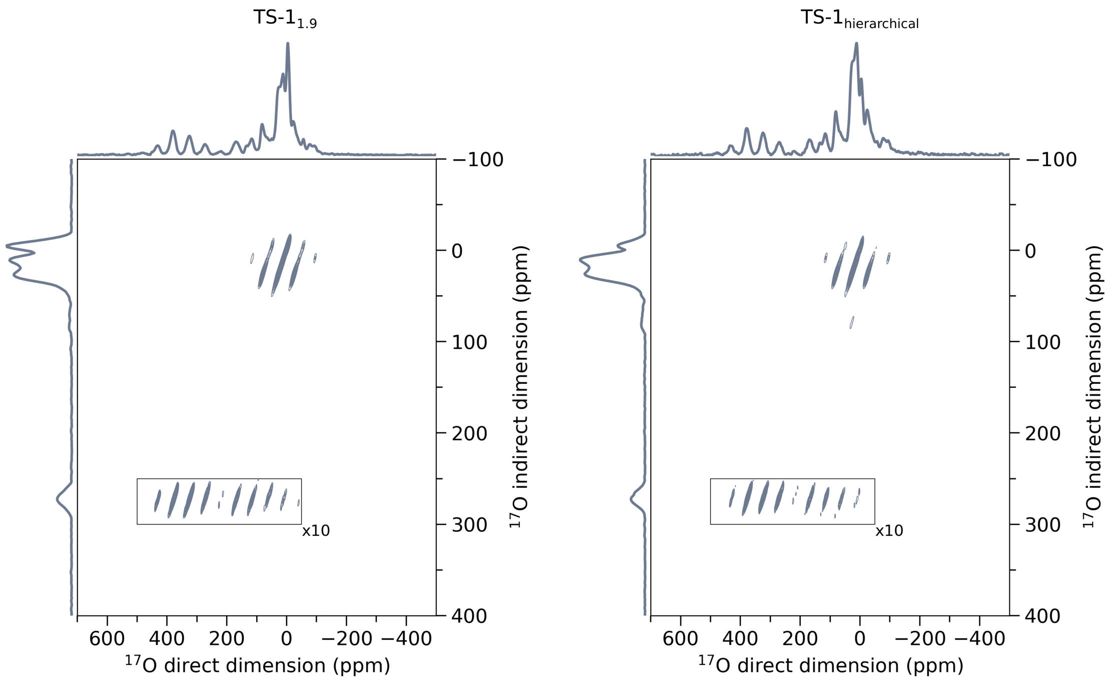

Figure S18: ^{17}O MAT (16.4 T) spectra of TS-1$_{1.9}$ and TS-1$_{hierarchical}$ (298 K, referenced to H_2O at room temperature (0 ppm)).

H.4 Fitting of Solid State NMR Spectra

All $^{47/49}Ti$ NMR spectra of the molecular library and all ^{17}O NMR spectra were fitted using int2QUAD model in DmFit.[20] For $^{47/49}Ti$ NMR spectra, only the central transition, δ_{iso} and EFG parameters were fitted. In case both isotopes (^{49}Ti and ^{47}Ti) were observable the respective signatures were fitted with the dependent parameters. For the ^{17}O NMR spectra also the CSA parameters were included into the fit. For CSA, the Herzfeld-Berger convention was used, where δ_{iso} is the isotropic chemical shift, $\Omega = \delta_{11} - \delta_{33}$, the span, and $\kappa = 3(\delta_{22} - \delta_{iso})/\Omega$ the skew. For EFG parameters, quadrupole coupling constant (C_Q) and asymmetry parameter (η_Q) were used.

Table S17: The fitted parameters for the $^{47/49}Ti$ solid-state NMR spectra of the TiO_2 references presented in this work.

Sample	Nuclei	MAS (kHz)	Amplitude	Line broadening (Hz)	δ_{iso} (ppm)	Ω (ppm)	κ	C_Q (MHz)	η_Q	φ	χ	ψ
**Anatase**	^{49}Ti	0	7.2×10^7	1000	-918	140	-0.87	3.9	0.0	0	30	0
**Anatase**	^{47}Ti	0	8.1×10^7	1500	-1185	140	-0.87	4.7	0.0	0	30	0
**Anatase**	^{49}Ti	20	2.3×10^8	1500	-918	140	-0.87	3.9	0.0	0	30	0
**Anatase**	^{47}Ti	20	1.9×10^8	1500	-1185	140	-0.87	4.7	0.0	0	30	0
**Rutile**	^{49}Ti	0	1.3×10^9	6000	-850	-	-	14.0	0.1	-	-	-
**Rutile**	^{47}Ti	0	3.0×10^8	6000	-1117	-	-	16.9	0.1	-	-	-
**Rutile**	^{49}Ti	20	7.0×10^8	2000	-850	-	-	14.0	0.1	-	-	-
**Rutile**	^{47}Ti	20	1.7×10^8	6000	-1117	-	-	16.9	0.1	-	-	-

Table S18: The fitted parameters for the $^{47/49}Ti$ solid-state NMR spectra of the molecular library presented in this work.

Sample	Nuclei	MAS (kHz)	Amplitude (ppm)	Line broadening (Hz)	δ_{iso} (ppm)	C_Q (MHz)	η_Q
**1**	^{49}Ti	0	5.9×10^4	1500	-933	1.7	0.40
**1**	^{47}Ti	0	3.2×10^4	1500	-1200	2.0	0.40
**2**	^{49}Ti	0	1.2×10^5	700	-914	4.0	0.90
**2**	^{47}Ti	0	1.2×10^4	700	-1181	4.8	0.90
**3**	^{49}Ti	0	1.2×10^5	6000	-716	13.1	0.92
**4**	^{49}Ti	0	8.6×10^4	4000	-776	28.7	0.05
**5**	^{49}Ti	0	1.0×10^5	15000	-840	22.1	0.25
**6**	^{49}Ti	0	3.2×10^5	10000	-798	16.9	1.0

Table S19: The fitted parameters for the ^{17}O solid-state NMR spectra presented in this work.

Sample	MAS (kHz)	Species	Amplitude	Line broadening (Hz)	δ_{iso} (ppm)	Ω (ppm)	κ	C_Q (MHz)	η_Q	φ	χ	ψ
**Silicalite-1**	0	Si-OH	9.3×10^3	500	3	120	0.00	2.8	1.0	0	20	0
**Silicalite-1**	0	Si-O-Si	5.9×10^4	600	39	80	0.38	5.4	0.1	0	10	0
**Silicalite-1**	5	Si-OH	1.9×10^3	500	3	120	0.00	2.8	1.0	0	20	0
**Silicalite-1**	5	Si-O-Si	1.7×10^3	600	39	80	0.38	5.4	0.1	0	10	0
**Silicalite-1**	8	Si-OH	1.1×10^3	500	3	120	0.00	2.8	1.0	0	20	0
**Silicalite-1**	8	Si-O-Si	1.0×10^3	600	39	80	0.38	5.4	0.1	0	10	0
**Silicalite-1**	14	Si-OH	2.3×10^3	500	3	120	0.00	2.8	1.0	0	20	0
**Silicalite-1**	14	Si-O-Si	2.2×10^3	600	39	80	0.38	5.4	0.1	0	10	0
**TS-1**$_1$	0	Si-OH	3.6×10^3	500	3	120	0.00	2.8	1.0	0	20	0
**TS-1**$_1$	0	Si-O-Si	2.3×10^4	600	39	80	0.38	5.4	0.1	0	10	0
**TS-1**$_1$	0	Ti-O-Si	4.2×10^3	300	280	444	0.73	3.0	0.2	0	20	0
**TS-1**$_1$	5	Si-OH	1.5×10^3	500	3	120	0.00	2.8	1.0	0	20	0
**TS-1**$_1$	5	Si-O-Si	1.6×10^4	600	39	80	0.38	5.4	0.1	0	10	0
**TS-1**$_1$	5	Ti-O-Si	7.6×10^2	300	280	444	0.73	3.0	0.2	0	20	0
**TS-1**$_1$	8	Si-OH	3.1×10^3	500	3	120	0.00	2.8	1.0	0	20	0
**TS-1**$_1$	8	Si-O-Si	3.6×10^3	600	39	80	0.38	5.4	0.1	0	10	0
**TS-1**$_1$	8	Ti-O-Si	2.0×10^3	300	280	444	0.73	3.0	0.2	0	20	0
**TS-1**$_1$	14	Si-OH	1.5×10^3	500	3	120	0.00	2.8	1.0	0	20	0
**TS-1**$_1$	14	Si-O-Si	2.1×10^3	600	39	80	0.38	5.4	0.1	0	10	0
**TS-1**$_1$	14	Ti-O-Si	1.7×10^3	300	280	444	0.73	3.0	0.2	0	20	0
**TS-1**$_{1.5}$	0	Si-OH	5.5×10^2	500	3	120	0.00	2.8	1.0	0	20	0
**TS-1**$_{1.5}$	0	Si-O-Si	4.1×10^3	600	39	80	0.38	5.4	0.1	0	10	0
**TS-1**$_{1.5}$	0	Ti-O-Si	7.6×10^2	300	280	444	0.73	3.0	0.2	0	20	0
**TS-1**$_{1.5}$	5	Si-OH	1.3×10^3	500	3	120	0.00	2.8	1.0	0	20	0
**TS-1**$_{1.5}$	5	Si-O-Si	6.7×10^2	600	39	80	0.38	5.4	0.1	0	10	0
**TS-1**$_{1.5}$	5	Ti-O-Si	4.4×10^2	300	280	444	0.73	3.0	0.2	0	20	0
**TS-1**$_{1.5}$	8	Si-OH	4.1×10^3	500	3	120	0.00	2.8	1.0	0	20	0
**TS-1**$_{1.5}$	8	Si-O-Si	2.3×10^3	600	39	80	0.38	5.4	0.1	0	10	0
**TS-1**$_{1.5}$	8	Ti-O-Si	1.8×10^3	300	280	444	0.73	3.0	0.2	0	20	0
**TS-1**$_{1.5}$	14	Si-OH	2.0×10^2	500	3	120	0.00	2.8	1.0	0	20	0
**TS-1**$_{1.5}$	14	Si-O-Si	1.4×10^2	600	39	80	0.38	5.4	0.1	0	10	0
**TS-1**$_{1.5}$	14	Ti-O-Si	1.5×10^2	300	280	444	0.73	3.0	0.2	0	20	0
**TS-1**$_{1.9}$	0	Si-OH	1.8×10^3	500	3	120	0.00	2.8	1.0	0	20	0
**TS-1**$_{1.9}$	0	Si-O-Si	1.3×10^4	600	39	80	0.38	5.4	0.1	0	10	0
**TS-1**$_{1.9}$	0	Ti-O-Si	3.1×10^3	300	280	444	0.73	3.0	0.2	0	20	0
**TS-1**$_{1.9}$	5	Si-OH	3.5×10^3	500	3	120	0.00	2.8	1.0	0	20	0
**TS-1**$_{1.9}$	5	Si-O-Si	2.2×10^3	600	39	80	0.38	5.4	0.1	0	10	0
**TS-1**$_{1.9}$	5	Ti-O-Si	1.5×10^3	300	280	444	0.73	3.0	0.2	0	20	0
**TS-1**$_{1.9}$	8	Si-OH	4.9×10^3	500	3	120	0.00	2.8	1.0	0	20	0
**TS-1**$_{1.9}$	8	Si-O-Si	3.1×10^3	600	39	80	0.38	5.4	0.1	0	10	0
**TS-1**$_{1.9}$	8	Ti-O-Si	2.5×10^3	300	280	444	0.73	3.0	0.2	0	20	0
**TS-1**$_{1.9}$	14	Si-OH	1.4×10^3	500	3	120	0.00	2.8	1.0	0	20	0
**TS-1**$_{1.9}$	14	Si-O-Si	9.3×10^2	600	39	80	0.38	5.4	0.1	0	10	0
**TS-1**$_{1.9}$	14	Ti-O-Si	1.1×10^3	300	280	444	0.73	3.0	0.2	0	20	0

Table S20: The fitted parameters for the ^{17}O solid-state NMR spectra presented in this work.

Sample	MAS (kHz)	Species	Amplitude	Line broadening (Hz)	δ_{iso} (ppm)	Ω (ppm)	κ	C_Q (MHz)	η_Q	φ	χ	ψ
**TS-1$_{hierarchical}$**	0	Si-OH	9.4 $\times 10^2$	500	3	120	0.00	2.8	1.0	0	20	0
**TS-1$_{hierarchical}$**	0	Si-O-Si	4.6 $\times 10^3$	600	39	80	0.38	5.4	0.1	0	10	0
**TS-1$_{hierarchical}$**	0	Ti-O-Si	1.2 $\times 10^3$	300	280	444	0.73	3.0	0.2	0	20	0
**TS-1$_{hierarchical}$**	5	Si-OH	6.2 $\times 10^2$	500	3	120	0.00	2.8	1.0	0	20	0
**TS-1$_{hierarchical}$**	5	Si-O-Si	9.9 $\times 10^2$	600	39	80	0.38	5.4	0.1	0	10	0
**TS-1$_{hierarchical}$**	5	Ti-O-Si	5.2 $\times 10^3$	300	280	444	0.73	3.0	0.2	0	20	0
**TS-1$_{hierarchical}$**	8	Si-OH	1.4 $\times 10^3$	500	3	120	0.00	2.8	1.0	0	20	0
**TS-1$_{hierarchical}$**	8	Si-O-Si	2.7 $\times 10^3$	600	39	80	0.38	5.4	0.1	0	10	0
**TS-1$_{hierarchical}$**	8	Ti-O-Si	1.8 $\times 10^3$	300	280	444	0.73	3.0	0.2	0	20	0
**TS-1$_{hierarchical}$**	14	Si-OH	7.6 $\times 10^2$	500	3	120	0.00	2.8	1.0	0	20	0
**TS-1$_{hierarchical}$**	14	Si-O-Si	1.7 $\times 10^3$	600	39	80	0.38	5.4	0.1	0	10	0
**TS-1$_{hierarchical}$**	14	Ti-O-Si	1.5 $\times 10^3$	300	280	444	0.73	3.0	0.2	0	20	0

Table S21: The integral ratios of fitted species in the ^{17}O solid-state MAS NMR spectra presented in this work.

Sample	MAS	Species	Integral ratio
**Silicalite-1**	5	Si-OH	24
**Silicalite-1**	5	Si-O-Si	76
**Silicalite-1**	8	Si-OH	24
**Silicalite-1**	8	Si-O-Si	76
**Silicalite-1**	14	Si-OH	26
**Silicalite-1**	14	Si-O-Si	74
**TS-1**$_1$	5	Si-OH	15
**TS-1**$_1$	5	Si-O-Si	60
**TS-1**$_1$	5	Ti-O-Si	25
**TS-1**$_1$	8	Si-OH	15
**TS-1**$_1$	8	Si-O-Si	60
**TS-1**$_1$	8	Ti-O-Si	26
**TS-1**$_1$	14	Si-OH	14
**TS-1**$_1$	14	Si-O-Si	60
**TS-1**$_1$	14	Ti-O-Si	26
**TS-1**$_{1.5}$	5	Si-OH	26
**TS-1**$_{1.5}$	5	Si-O-Si	47
**TS-1**$_{1.5}$	5	Ti-O-Si	27
**TS-1**$_{1.5}$	8	Si-OH	24
**TS-1**$_{1.5}$	8	Si-O-Si	47
**TS-1**$_{1.5}$	8	Ti-O-Si	28
**TS-1**$_{1.5}$	14	Si-OH	23
**TS-1**$_{1.5}$	14	Si-O-Si	49
**TS-1**$_{1.5}$	14	Ti-O-Si	29
**TS-1**$_{1.9}$	5	Si-OH	22
**TS-1**$_{1.9}$	5	Si-O-Si	48
**TS-1**$_{1.9}$	5	Ti-O-Si	30
**TS-1**$_{1.9}$	8	Si-OH	22
**TS-1**$_{1.9}$	8	Si-O-Si	48
**TS-1**$_{1.9}$	8	Ti-O-Si	30
**TS-1**$_{1.9}$	14	Si-OH	23
**TS-1**$_{1.9}$	14	Si-O-Si	47
**TS-1**$_{1.9}$	14	Ti-O-Si	30
**TS-1**$_{\mathbf{hierarchical}}$	5	Si-OH	11
**TS-1**$_{\mathbf{hierarchical}}$	5	Si-O-Si	61
**TS-1**$_{\mathbf{hierarchical}}$	5	Ti-O-Si	29
**TS-1**$_{\mathbf{hierarchical}}$	8	Si-OH	9
**TS-1**$_{\mathbf{hierarchical}}$	8	Si-O-Si	60
**TS-1**$_{\mathbf{hierarchical}}$	8	Ti-O-Si	31
**TS-1**$_{\mathbf{hierarchical}}$	14	Si-OH	9
**TS-1**$_{\mathbf{hierarchical}}$	14	Si-O-Si	60
**TS-1**$_{\mathbf{hierarchical}}$	14	Ti-O-Si	31

Table S22: The fitted parameters for the ^{17}O solid-state NMR (9.4 T) spectra presented in this work.

Sample	MAS (kHz)	Species	Amplitude	Line broadening (ppm)	δ_{iso} (ppm)	Ω (ppm)	κ	C_Q (MHz)	η_Q	φ	χ	ψ
**TS-1**$_{1.9}$	0	Si-OH	1.2 $\times 10^5$	500	3	120	0.00	2.8	1.0	0	20	0
**TS-1**$_{1.9}$	0	Si-O-Si	6.2 $\times 10^5$	600	39	80	0.38	5.4	0.1	0	10	0
**TS-1**$_{1.9}$	0	Ti-O-Si	4.0 $\times 10^5$	800	280	444	0.73	3.0	0.2	0	20	0
**TS-1**$_{1.9}$	5	Si-OH	4.6 $\times 10^4$	500	3	120	0.00	2.8	1.0	0	20	0
**TS-1**$_{1.9}$	5	Si-O-Si	1.9 $\times 10^4$	600	39	80	0.38	5.4	0.1	0	10	0
**TS-1**$_{1.9}$	5	Ti-O-Si	2.1 $\times 10^4$	800	280	444	0.73	3.0	0.2	0	20	0
**TS-1**$_{1.9}$	8	Si-OH	7.6 $\times 10^4$	500	3	120	0.00	2.8	1.0	0	20	0
**TS-1**$_{1.9}$	8	Si-O-Si	4.3 $\times 10^4$	600	39	80	0.38	5.4	0.1	0	10	0
**TS-1**$_{1.9}$	8	Ti-O-Si	5.9 $\times 10^4$	1000	280	444	0.73	3.0	0.2	0	20	0
**TS-1**$_{1.9}$	14	Si-OH	1.5 $\times 10^4$	800	3	120	0.00	2.8	1.0	0	20	0
**TS-1**$_{1.9}$	14	Si-O-Si	1.0 $\times 10^4$	600	39	80	0.38	5.4	0.1	0	10	0
**TS-1**$_{1.9}$	14	Ti-O-Si	1.1 $\times 10^4$	500	280	444	0.73	3.0	0.2	0	20	0

H.5 Extended Czjzek Simulations

The numerical lineshapes for the $^{47/49}$Ti CPMG-MAS NMR spectra (Figures 2 and 3 in the main text) are based on the extension of the Czjzek model.[28,29] The bivariate distributions for the ^{49}Ti quadrupole parameters $C_Q(^{49}Ti)$ and $\eta_Q(^{49}Ti)$ were created using a laboratory-built Python script. For the 2D histograms, $2 \cdot 10^5$ random EFG tensors were sampled and binned into 51^2 pixel (51 bins per dimension), covering intervals of $C_Q \in [0, 15]$ MHz and $\eta_Q \in [0, 1]$. Note that for C_Q only the absolute value was considered. The corresponding $^{47/49}$Ti NMR spectra were simulated using the SIMPSON simulation package.[30] For the ^{47}Ti NMR signal, a fixed isotropic offset of -267 ppm with respect to the ^{49}Ti isotropic chemical shift was considered. The ^{47}Ti quadrupole parameters were derived from the ^{49}Ti quadrupole parameters according to $C_Q(^{47}Ti) = 1.22 * C_Q(^{49}Ti)$ and $\eta_Q(^{47}Ti) = \eta_Q(^{49}Ti)$. Lastly, all contributions with probability $< 5 \cdot 10^{-4}$ were excluded from the lineshape simulation.

Table S23: The extended Czjzek simulation parameters of the ^{49}Ti nuclei used for the respective TS-1 sample.

Sample	δ_{iso} (ppm)	$C_{Q,0}$ (MHz)	$\eta_{Q,0}$	ϵ
**TS-1$_1$**	-900	7.6	0.2	0.7
**TS-1$_{1.5}$**	-900	7.2	0.2	0.7
**TS-1$_{1.9}$**	-900	7.2	0.2	0.7

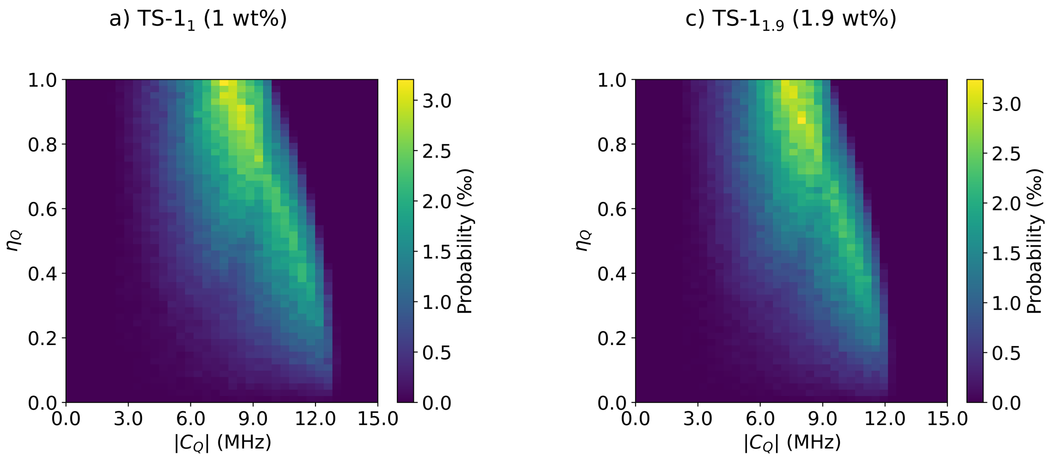

Figure S19: 2D histograms of the ^{49}Ti quadrupole coupling parameters used for the extended Czjzek model simulation of TS-1$_1$ and TS-1$_{1.9}$ in Figure 3 (the distribution of TS-1$_{1.5}$ is shown in the main text Figure 2b).

H.6 Spin Counting

In order to estimate the Ti wt% faction of anatase in TS-$1_{\text{hierarchical}}$ we turned to spin counting. Therefore we compared the $^{47/49}$Ti MAS NMR spectra of hydrated TS-$1_{\text{hierarchical}}$ and anatase nanoparticles (see Figure 4aII), measured with similar acquisition parameters (differences: number of scans (anatase) = 208, number of scans (TS-1) = 370728, m(anatase) = 46.6 mg, m(TS-1) = 24.3 mg, see Section H.2.1 for further details). Since both spectra were acquired with CMPG detection, one should note, that the intensity ratio between distinct species could be distorted due to different effective T2 relaxation times. In the following analysis we assume similar effective T2 relaxation constants for both extra-framework anatase-like TiO_2 and bulk anatase TiO_2. The resulting signal to noise ratios (S/N) are given by 36.8 for anatase and 10.4 for TS-$1_{\text{hierarchical}}$. Since ca. double the sample could be packed for anatase compared to hydrated TS-$1_{\text{hierarchical}}$ and the S/N is by a factor of 3.5 higher in the spectrum of anatase compared to the spectrum of hydrated TS-$1_{\text{hierarchical}}$, we expect a similar S/N for anatase (adjusting for both these factors: (i) same sample mass and (ii) same S/N) to be achieved in *ca.* 61 scans $(((1.9)^2 \cdot 208)/(3.5)^2)$. In a last step we calculate what Ti wt% loading of extra-framework TiO_2 (anatase) we expect in the TS-$1_{\text{hierarchical}}$, knowing that the Ti wt% of anatase is 60%:

The Ti wt% in TS-$1_{\text{hierarchical}}$ attributed to TiO_2 is smaller than the Ti wt% in pure anatase, by an unknown scaling factor a

$$\text{Ti wt\% from TiO}_2 \text{ fraction in TS-1} = \frac{\text{Ti wt\% in anatase}}{a} \tag{H.1}$$

Similarly, we expect the number of scans required for hydrated TS-1 (only anatase-like feature present) to be proportional to the number of scans needed for anatase (adjusting for both factors sample mass and S/N) and the square of the scaling factor a

$$\text{number of scans (TS-1)} = \text{number of scans (anatase)} \cdot a^2 \tag{H.2}$$

By combining both expressions we obtain:

$$\begin{aligned} \text{Ti wt\% from TiO}_2 \text{ fraction in TS-1} &= \sqrt{\frac{\text{number of scans (anatase)} \cdot (\text{Ti wt\% in anatase})^2}{\text{number of scans (TS-1)}}} \\ &= \sqrt{\frac{61 \cdot 60^2}{370728}} = 0.77 \end{aligned} \tag{H.3}$$

From this analysis we could therefore concluded, that *ca.* 0.8 Ti wt% of the total 1.9 Ti wt% in TS-$1_{\text{hierarchical}}$ is anatase.

H.7 Discussion of Extra-framework TiO_2 in high weight loading classical TS-1

Additionally, we explored a high weight loading *classical* TS-1 (3.5 Ti wt%) catalyst containing large amounts of extra-framework TiO_2 as confirmed by UV-Vis spectroscopy (see Figure S1), referred to as TS-$1_{3.5\ \text{extra-framework}}$. Not surprisingly, TS-$1_{3.5\ \text{extra-framework}}$ therefore displays $^{47/49}$Ti NMR signatures in both the hydrated and dehydrated state (see Figure S20I), which points towards the presence of extra-framework TiO_2 sites. This is in drastic contrast to the other *classical* TS-1 samples (1.0 - 1.9 wt%), for which the $^{47/49}$Ti NMR signal vanishes upon hydration.

In fact, the $^{47/49}$Ti NMR signature of TS-$1_{3.5\ \text{extra-framework}}$ in the hydrate state is reminiscent of the signature of the hydrated TS-$1_{\text{hierarchical}}$. However, it does not match exactly the signature of anatase TiO_2 nanoparticles (see Figure S20II). Whereby most notable is the change in the ratio between the two NMR active Ti nuclei (^{47}Ti and ^{49}Ti). The origin of this change is proposed to be relaxation, since the ^{47}Ti nuclei relaxes faster compared to the ^{49}Ti nuclei, due to its larger quadrupole moment, leading to over-pronounced ^{47}Ti intensities for long relaxing sites. Since, the remaining signature of the hydrated TS-$1_{3.5\ \text{extra-framework}}$ displays lower ^{47}Ti intensities compared to the anatase TiO_2 nanoparticles, we postulate that the observed species in hydated TS-$1_{3.5\ \text{extra-framework}}$ has faster relaxation properties. Therefore, we propose that this signature corresponds to smaller anatase-like TiO_x clusters.

Subtraction of the $^{47/49}$Ti NMR signature of TS-$1_{3.5\ \text{extra-framework}}$ in the hydrated state from the corresponding signature in the dehydrated state reveals that the remaining signature matches the spectrum of the dehydrated model TS-$1_{1.5}$ (see Figure S20III). Therefore, this signature was assigned to framework Ti sites.

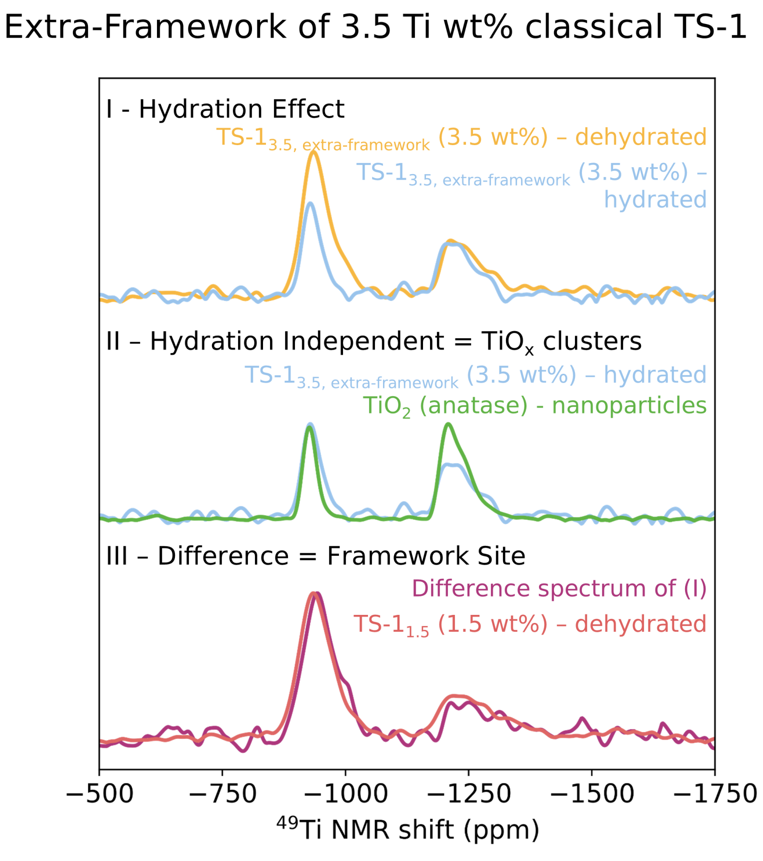

Figure S20: Detection of extra-framework TiO_x clusters in a high weight loading classical TS-1 catalyst (TS-$1_{3.5\ \text{extra-framework}}$). (I) Effect of hydration on the $^{47/49}$Ti NMR signature of TS-$1_{3.5\ \text{extra-framework}}$. (II) $^{47/49}$Ti NMR signature of hydrated TS-$1_{3.5\ \text{extra-framework}}$ superimposed with the signature of TiO_2 (anatase). (III) Difference spectrum of the normalised TS-$1_{3.5\ \text{extra-framework}}$ hydrated/dehydrated spectra plotted with the spectrum of dehydrated TS-$1_{1.5}$.

I DFT Calculations

I.1 General Details

All computations were performed on the high-performance computing (HPC) cluster EULER (Erweiterbarer, Umweltfreundlicher, Leistungsfähiger ETH-Rechner).

I.2 Periodic Geometry Optimizations

The periodic geometry optimization calculations[31] were performed with the BP86[32,33] GGA functional in combination with an all-electron DZP basis set[34] for all main group elements and an all-electron TZP basis set[34] for Ti, using the Amsterdam Modeling Suite (AMS)[35] BAND (version 2023.101)[36,37] program suite. Unless specifically noted, the calculations were performed with fixed lattice parameters, default convergence criteria and the relativity level settings set to none. In case elements of the fifth or higher row are present in the structure, the scalar relativistic zeroth order regular approximation (ZORA)[38–40] approach was employed.

I.2.1 Molecules

Depending on whether the experimental NMR data was obtained via solution or solid state NMR the periodic optimization of Ti molecules was varied. In order to compare with solution NMR data the initial guesses of the molecules were placed in a large unit cell of 20.0 Å × 20.0 Å × 20.0 Å to ensure the unperturbed optimization (these structures are labeled with the * symbol). The same protocol was employed for all organic molecules and methyltrioxorhenium (MTO). For the comparison with the solid state $^{47/49}$Ti NMR data of the Ti-complexes molecular library (**1-6** and **IX-XII**) the experimental cif files were used as initial guess and periodically optimized: [Ti(OTBOS)$_4$] (**1**) (CCDC no. 2205478),[5] [Ti(O^{i}Pr)(OTBOS)$_3$] (**2**) (CCDC no. 1882540),[12] [Ti(NMe$_2$)(OTBOS)$_3$] (**3**) (CCDC no. 2361357),[7] [TiCl(OTBOS)$_3$] (**4**) (CCDC no. 2482638), Ti-pentadentate-salan (**5**) (CCDC no. 2306102)[8] and [Ti$_2$O$_2$(acac)$_4$] (**6**) (CCDC no. 177977),[13] TiCp$_2$Cl$_2$ (**IX**) (CCDC no. 113637),[41] TiCp*2Cl$_2$ (**X**) (CCDC no. 1235253)[42] TiCp*Cl$_3$, (**XI**) (CCDC no. 194086),[43] [H$_2$NMe$_2$]$_2$[TiCl$_6$] (**XII**) (CCDC no.197282).[44] For [Ti(OTBOS)$_4$] (**1**) and [Ti(O^{i}Pr)(OTBOS)$_3$] (**2**) the convergence criteria were adjusted (Gradients 0.05 Hartree/Å, Step 0.05 Å).

I.2.2 Silicalite-1 and TS-1 Framework (T-Site) Model Generation

For the zeolitic structures the parent Silicalite-1[17] (ICSD Collection Code: 280364, ICSD release 2025.1)[18] structure were used, with the lattice parameters adjusted to the experimentally obtained values at 296 K (see Section G, Table S4). For the Silicalite-1 model the reported structure was directly used for the periodic structure optimization. For TS-1s, the different T-site models (T1-T12) were obtained via isomorphous substitution of Si with Ti in the respective T-Site. In case of dinuclear model generation two neighboring Si were replaced with Ti (in this work T7-T7). After the periodic optimization, clusters of the different T-sites were cut and terminated in the third coordination sphere by fluorine atoms and used without further optimization, yielding clusters of the following form Ti(OSiF$_3$)$_4$ (Si(OSiF$_3$)$_4$ for the Silicalite-1 model).

I.2.3 TS-1 Framework Associated Site Model Generation

For the framework associated sites (**B-F**) the T-site with the $^{47/49}$Ti NMR parameters closest to the experimental values of TS-1 (T8, **A**, see Section I.3.2) was used as starting point. For defect generation neighboring Si atoms were removed and dangling bonds were terminated by hydrogen atoms. For sites due to hydration, the addition of water molecules were used to form hydrated bonds. After the periodic structure optimization the same procedure, as for the TS-1 framework (T-Site) models, was used for the cluster generation.

I.3 ^{49}Ti NMR Parameter Calculations

The optimized structures employed in the NMR parameter calculation were obtained as described in Section I.2. $^{47/49}$Ti NMR parameter calculations[45–47] employed an all-electron scalar relativistic ZORA[38–40] approach using the hybrid B3LYP[48,49] functional and TZ2P[34] basis sets on all elements, in the Amsterdam Modeling Suite (AMS)[35] ADF[50] (version 2023.101) program suite. The numerical quality was set to very good and symmetry was set to NOSYM.

I.3.1 ^{49}Ti NMR Parameter Benchmark and Molecular Model Systems

I.3.1.1 ^{49}Ti Chemical Shift

The calculated ^{47}Ti NMR shielding values of the benchmark structures yielded very good agreement (linear relationship) with the experimental ^{49}Ti chemical shift values, similar to what has been previously reported.[51–54] The benchmark set (**I*-IX***) is shown below. In general, the ^{49}Ti chemical shift of the computed structures i ($\delta_{\text{calc},i}(^{49}\text{Ti})$) were obtained from the calculated ^{47}Ti NMR shielding values ($\sigma_{\text{calc},i}(^{47}\text{Ti})$) via the correlation obtained from the benchmark set (Equation I.1).

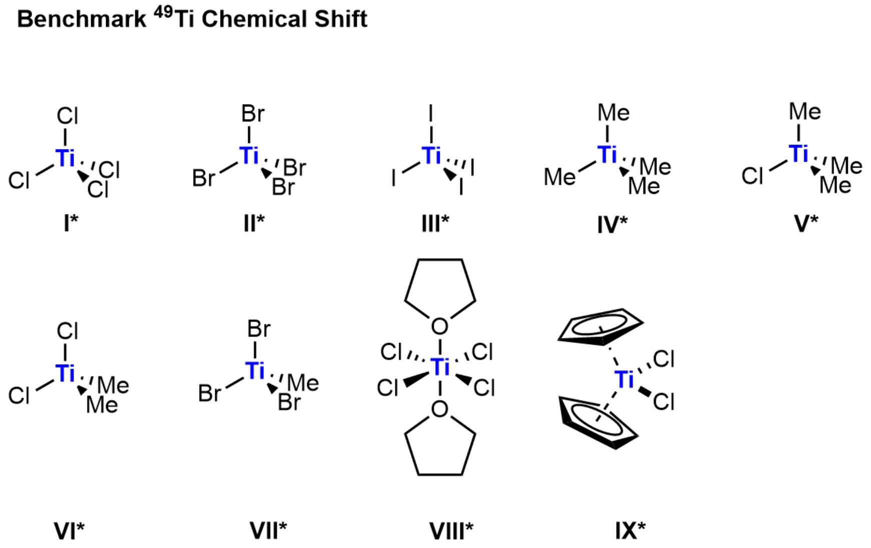

Figure S21: Library of Ti-complexes with perviously reported solution ^{49}Ti NMR chemical shifts.

$$\delta_{\text{calc},i}(^{49}\text{Ti}) = -860.0 - 1.007 \times \sigma_{\text{calc},i}(^{47}\text{Ti}) \quad \text{(I.1)}$$

Table S24: Summary of computed and experimental ^{49}Ti solution NMR parameters of the chemical shift benchmark set (see Figure S21). Calculated shielding $\sigma_{\text{calc}}(^{47}\text{Ti})$ obtained via the protocol described in Section I.3, calculated chemical shift $\delta_{\text{calc}}(^{49}\text{Ti})$, obtained via the correlation plotted in Figure S22, and experimental ^{49}Ti NMR chemical shift $\delta_{\text{exp}}(^{49}\text{Ti})$ taken from literature.[52,55,56]

	Formula	$\sigma_{\text{calc}}(^{47}\text{Ti})$ (ppm)	$\delta_{\text{calc}}(^{49}\text{Ti})$ (ppm)	$\delta_{\text{exp}}(^{49}\text{Ti})$ (ppm)
**I***	$TiCl_4$	-901	47	0
**II***	$TiBr_4$	-1402	553	483[55]
**III***	TiI_4	-2165	1320	1278[55]
**IV***	$TiMe_4$	-2171	1326	1325[52]
**V***	$TiMe_3Cl$	-1957	1111	1188[52]
**VI***	$TiMe_2Cl_2$	-1672	824	907[52]
**VII***	$TiMeBr_3$	-1683	835	825[56]
**VIII***	*trans*-$TiCl_4(thf)_2$	-672	-183	-240[56]
**IX***	$TiCp_2Cl_2$	-20	-840	-772[55]

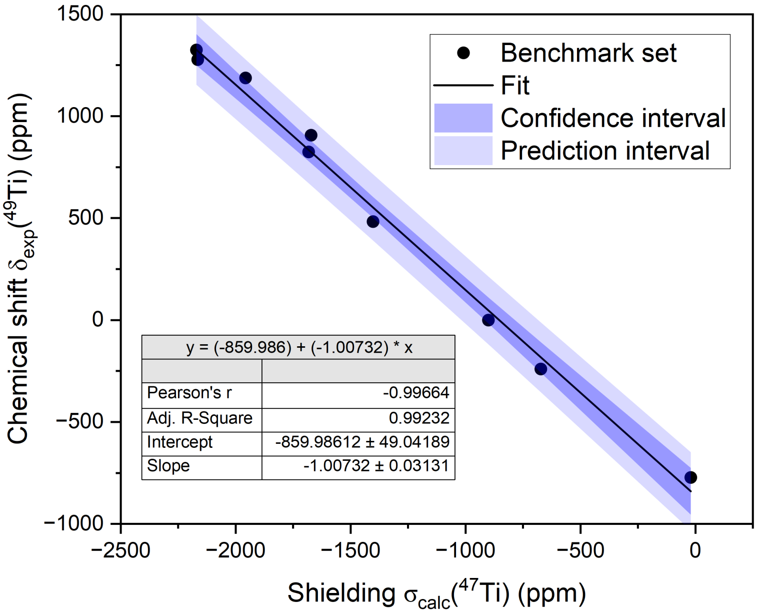

Figure S22: Correlation between experimental chemical shift $\delta_{\text{exp}}(^{49}\text{Ti})$ values taken from literature[52,55,56] and the respective calculated shielding $\sigma_{\text{calc}}(^{47}\text{Ti})$ values.

I.3.1.2 ^{49}Ti Quadrupole coupling constant

For the evaluation of the ^{49}Ti quadrupole coupling constant calculations ($C_{Q,\ calc}$), computed values were compared with the experimental values ($C_{Q,\ exp}$), reported in literature[21,54] (**IX-XII**) and measured by us (**1-6**). Overall, the calculations showed very good agreement (linear relationship with a slope close to 1) with the experimental values (see Figure S24).

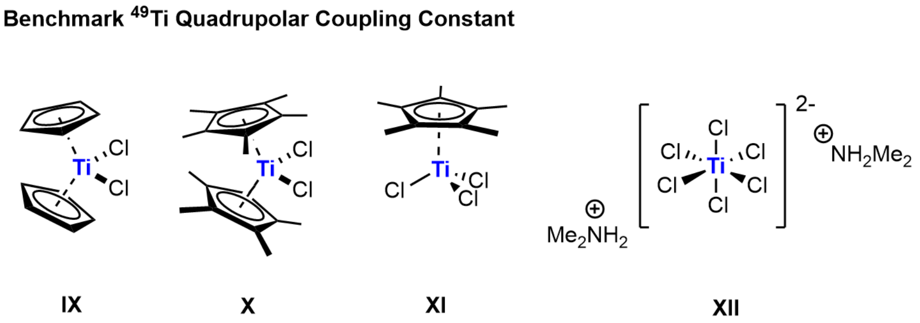

Figure S23: Library of Ti-complexes with perviously reported ^{49}Ti quadrupole coupling constants.

Table S25: Calculated quadrupole coupling constants via the protocol described in Section I.3 and experimental quadrupole coupling constants taken from literature.[21,54]

	Formula	$C_{Q,\ calc}$ (MHz)	$C_{Q,\ exp}$ (MHz)
**IX**	$TiCp_2Cl_2$	6.5	4.2[21]
**X**	$TiCp^*_2Cl_2$	5.7	5.5[21]
**XI**	$TiCp^*Cl_3$	2.7	3.0[21]
**XII**	$[H_2NMe_2]_2[TiCl_6]$	2.3	3.0[54]

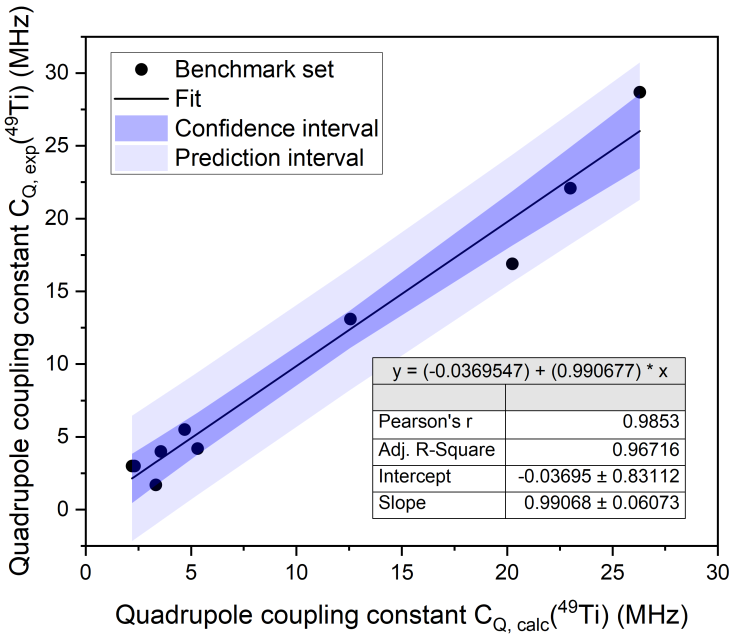

Figure S24: Correlation between the experimental $C_{Q,\ exp}(^{49}Ti)$ values taken from literature[21,54] (see Figure S23) and of the molecular library (see Figure S25) measured by us with the respective calculated $C_{Q,\ calc}(^{49}Ti)$ values.

I.3.1.3 ^{49}Ti NMR Signatures of the Molecular Library

The molecular library of Ti complexes (**1-6**) reminiscent of framework or possible framework associated Ti sites in TS-1 was used to estimate the error in the DFT protocol, including the periodic optimization and the ^{49}Ti NMR parameter calculations ($\delta_{iso}(^{49}Ti)$ and $C_Q(^{49}Ti)$, see Section I.5).

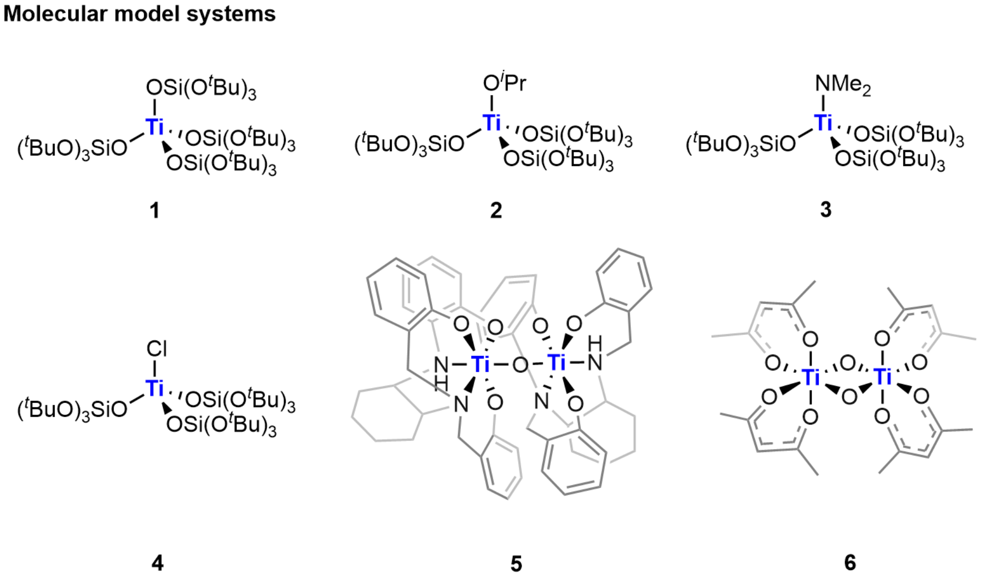

Figure S25: Molecular library of Ti-complexes closely related to the studied Ti-sites in TS-1.

Table S26: Summary of computed and experimental ^{49}Ti solid-state NMR parameters of the molecular library (**1-6**). Calculated shielding $\sigma_{calc}(^{47}Ti)$ obtained via the protocol described in Section I.3, calculated chemical shift $\delta_{calc}(^{49}Ti)$, obtained via the correlation plotted in Figure S22, and experimental Ti NMR chemical shift $\delta_{exp}(^{49}Ti)$.

	Formula	$\sigma_{calc}(^{47}Ti)$ (ppm)	$\delta_{calc}(^{49}Ti)$ (ppm)	$\delta_{exp}(^{49}Ti)$ (ppm)
**1**	$[Ti(OTBOS)_4]$	58	-918	-933
**2**	$[Ti(O^iPr)(OTBOS)_3]$	68	-929	-914
**3**	$[Ti(NMe_2)(OTBOS)_3]$	-202	-657	-716
**4**	$[TiCl(OTBOS)_3]$	-94	-765	-776
**5**	Ti-pentadentate-salan	-16	-844	-840
**6**	$[Ti_2O_2(acac)_4]$	-91	-768	-798

Table S27: Calculated ^{49}Ti quadrupole coupling constants via the protocol described in Section I.3 and the experimentally obtained quadrupole coupling constants.

	Formula	$C_{Q,\ calc}$ (MHz)	$C_{Q,\ exp}$ (MHz)
**1**	$[Ti(OTBOS)_4]$	3.3	1.7
**2**	$[Ti(O^iPr)(OTBOS)_3]$	3.6	4.0
**3**	$[Ti(NMe_2)(OTBOS)_3]$	12.6	13.1
**4**	$[TiCl(OTBOS)_3]$	26.3	28.7
**5**	Ti-pentadentate-salan	23.0	22.1
**6**	$[Ti_2O_2(acac)_4]$	20.2	16.9

For a direct comparison of the computed $^{47/49}$Ti NMR parameters and the experimentally obtained values, the experimental data are plotted along with the simulated lineshape based on the computed $^{47/49}$Ti NMR parameters (in case the fitted ^{49}Ti NMR signature has a $C_Q \leq 10$ MHz the ^{47}Ti NMR signature was also fitted, in case of larger C_Q values, the ^{47}Ti NMR was not observed).

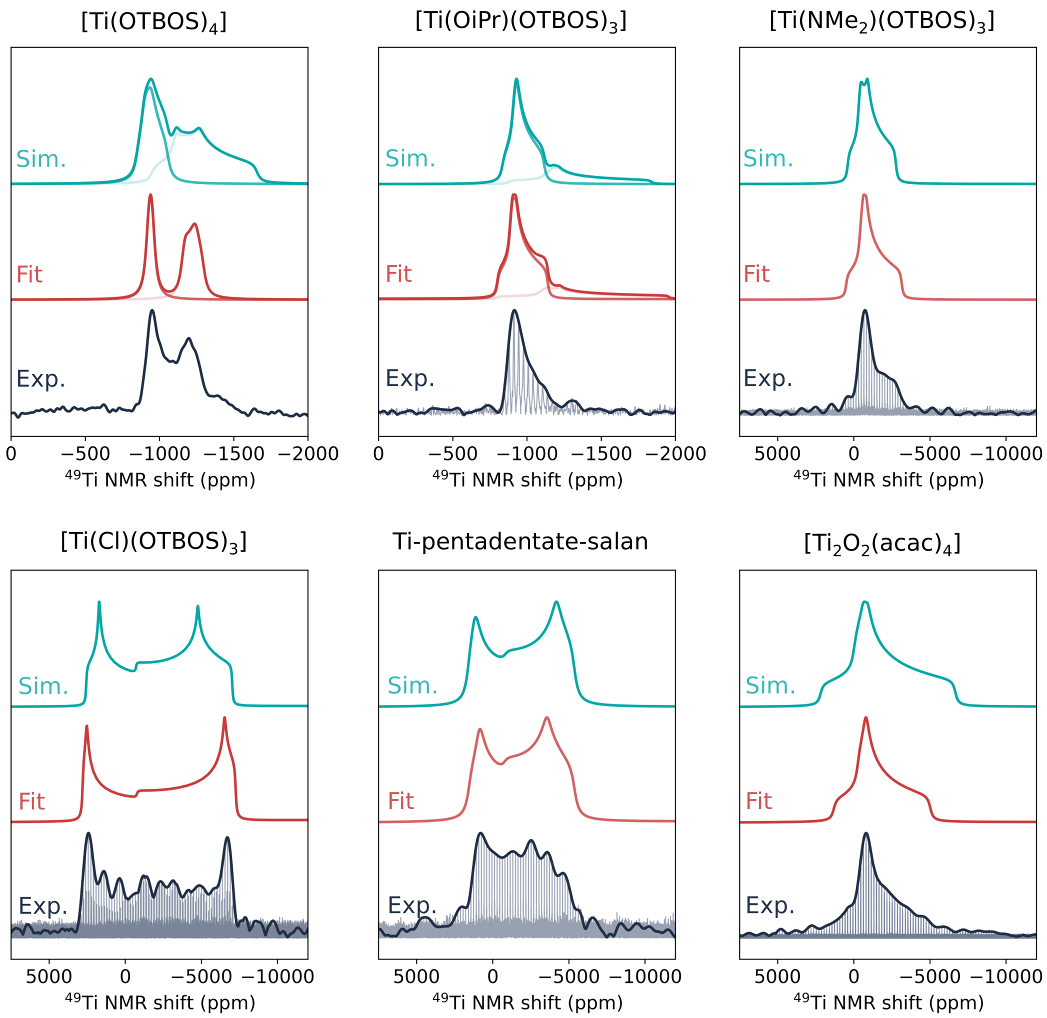

Figure S26: $^{47/49}$Ti NMR spectra of the molecular library of Ti complexes (**1**-**6**) in *grey*, with the respective fits (*red*) and simulated lineshape based on the computed $^{47/49}$Ti NMR parameters (*turquoise*).

I.3.2 ^{49}Ti NMR Signatures of Ti-zeotypes

The ^{49}Ti NMR parameters of the different T-sites in the investigated Ti-zeotypes were calculated from the $Ti(OSiF_3)_4$ cluster models obtained as described in Section I.2.2.

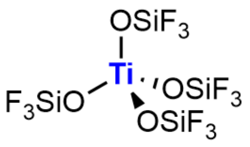

Figure S27: T-site cluster models for mononuclear ($Ti(OSiF_3)_4$).

I.3.2.1 Ti Speciation: TS-1 Framework and Framework Associated Sites

In terms of Ti site speciation we considered the following structures as possible candidates: Fully incorporated Ti framework structure (**A**), tetrahedral titanol (**B**) and dititanol (**C**) sites, and higher coordination Ti sites, namely hexa-coordinated dititanol species (**D**), penta-coordinated bridging titanol (**E**) and hexa-coordinated bridging dititanol (**F**) sites. The computed ^{49}Ti NMR parameters are summarized in the Table S28.

Table S28: Summary of ^{49}Ti NMR signatures of framework and framework associated sites for TS-$1_{1.5}$.

Species	TS-$1_{1.5}$	
	δ_{iso}(ppm)	C_Q (MHz)
**A**	-883	6.7
**B**	-812	12.4
**C**	-765	20.2
**D**	-813	37.7
**E**	-870	31.5
**F**	-828	42.3

I.3.2.2 Ti Location in the Framework: TS-1 Framework T-Sites

In this section we focus on the Ti location in the TS-1 framework (T-sites). The computed ^{49}Ti NMR parameters are summarized in the Table S29.

Table S29: Summary of ^{49}Ti NMR signatures of all T-sites for the classical TS-1 samples with minimal amount of TiO_2.

T-site	TS-1_1		TS-$1_{1.5}$		TS-$1_{1.9}$	
	δ_{iso}(ppm)	C_Q (MHz)	δ_{iso}(ppm)	C_Q (MHz)	δ_{iso}(ppm)	C_Q (MHz)
**T1**	-863	7.0	-873	7.8	-876	7.9
**T2**	-863	11.0	-863	11.1	-859	11.0
**T3**	-853	8.3	-854	7.5	-854	7.7
**T4**	-890	10.4	-891	10.1	-891	10.4
**T5**	-829	7.5	-830	7.7	-828	8.5
**T6**	-854	10.3	-849	11.1	-855	11.1
**T7**	-840	9.0	-840	9.0	-839	8.0
**T8**	-880	7.9	-883	6.7	-882	7.3
**T9**	-843	11.0	-845	10.4	-843	10.2
**T10**	-824	9.5	-822	9.4	-823	9.5
**T11**	-839	10.7	-840	10.4	-841	10.0
**T12**	-856	9.9	-859	9.3	-858	9.9

Next, numeric simulations considering all selected T-sites (see manuscript for further details) and their associated calculated NMR parameters (see Table S29) were performed employing the DMFit software.[20] For these numeric simulations both the ^{47}Ti and ^{49}Ti isotope were considered. The ^{47}Ti NMR parameters were obtained based on the calculated ^{49}Ti NMR parameters, shifted by the resonant frequency difference between the two isotopes and with an increased quadrupole coupling based on the increased quadrupole moment. Further, equal population was assumed across the selected T-sites, hence the integral of all sites were matched. All η_Q values were set to 0.7, removing the site dependency on the asymmetry parameter and allowing to capture the experimental $^{47/49}$Ti NMR signatures. The MAS spectra are presented in the main text Figure 8. The static spectra are shown in Figure S28 for completeness, highlighting that both simulations considering either (i) all T-sites or (ii) selected T-sites are able to describe the observed static spectrum.

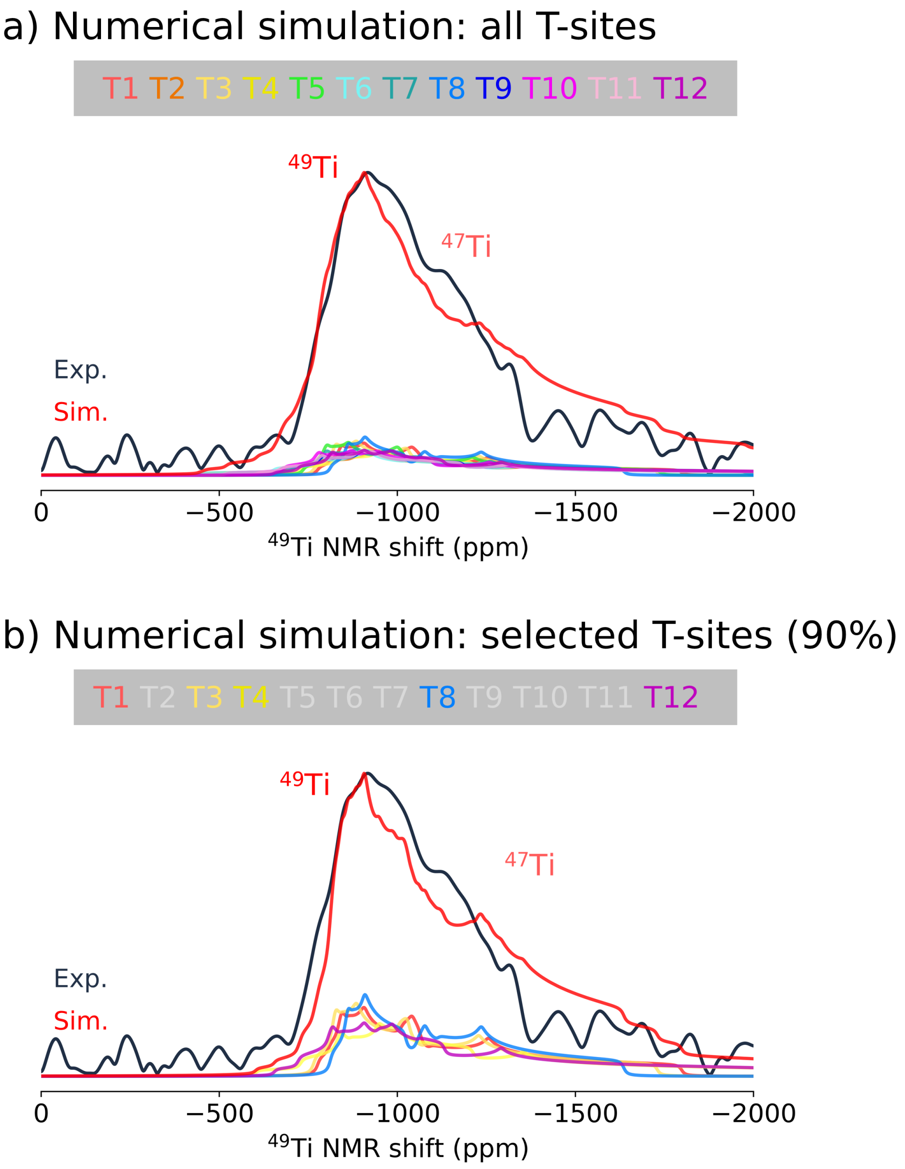

Figure S28: Comparison between the experimental $^{47/49}$Ti NMR signature of TS-$1_{1.5}$ and numeric simulations based on the calculated $^{47/49}$Ti NMR parameters δ_{iso} and C_Q (η_Q was set to 0.7 for all considered sites) for (a) all T-sites and (b) selected T-sites (below the 90% threshold), assuming equal population of the considered sites.

I.3.3 ^{49}Ti NMR Parameters Dependency of Ti(OSiF$_3$)$_4$ Cluster Model on Ti-O Bond Length, O-Ti-O and Ti-O-Si Bond Angle

In order to examine the ^{49}Ti NMR parameters dependency on structural variations of the tetrahedral environment, we started from the optimized framework model **A** (Ti(OSiF$_3$)$_4$, obtained as described in Section I.2). In a next step, selective bond length or bond angle perturbations (Ti-O bond length, O-Ti-O bond angel and Ti-O-Si bond angle) in the framework model **A** were introduced, while keeping the other atom positions fixed, as depicted in Figure S29. The ^{49}Ti NMR parameters were calculated for the resulting structures without further optimization. The obtained $\delta_{\text{iso}}(^{49}\text{Ti})$ and $C_Q(^{49}\text{Ti})$ values are depicted in Figure S30-S32. In a final step the ^{49}Ti NMR parameters of all geometrical perturbations were compared to the experimental values of the dominant Ti site in TS-1 (*red* line) and their associated 95% confidence limits based on the χ^2_R (*grey* lines, see Section I.5 for further details). In comparison to the ^{17}O NMR parameters (see Section I.4.3) the ^{49}Ti NMR parameters are significantly more responsive to small structural changes, hence most considered models fall outside the 95% confidence limits.

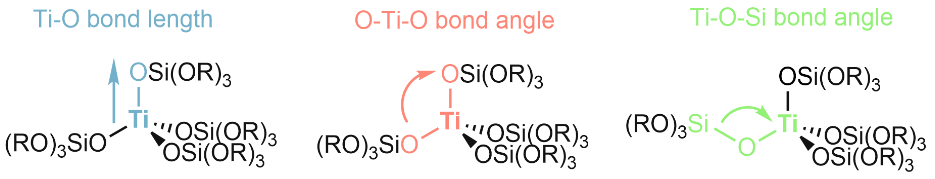

Figure S29: The dependency of the ^{49}Ti NMR parameters δ_{iso} and C_Q of the Ti(OSiF$_3$)$_4$ cluster model on the Ti-O bond length, O-Ti-O angle and Ti-O-Si angle.

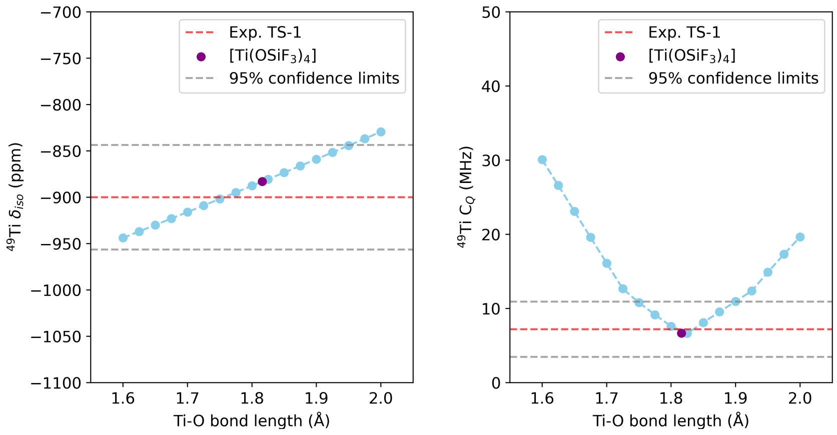

Figure S30: The dependency of the ^{49}Ti NMR parameters δ_{iso} and C_Q of the Ti(OSiF$_3$)$_4$ cluster model on the Ti-O bond length. As reference points the ^{49}Ti NMR parameters of the optimized framework model **A** are depicted (*violet*).

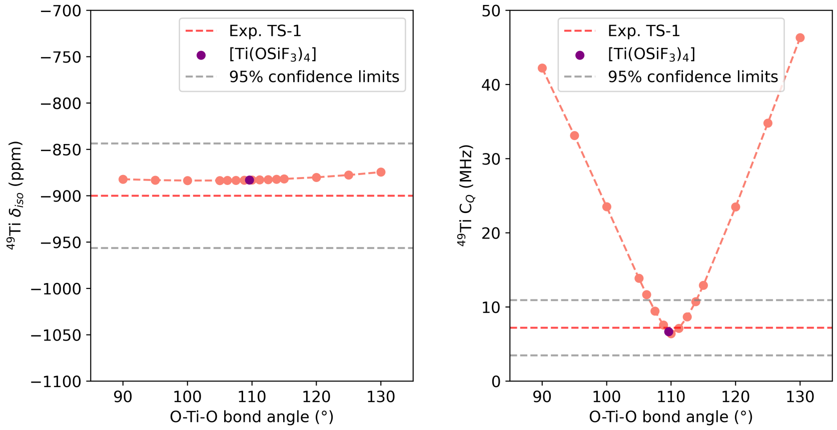

Figure S31: The dependency of the ^{49}Ti NMR parameters δ_{iso} and C_Q of the Ti(OSiF$_3$)$_4$ cluster model on the O-Ti-O bond angle. As reference points the ^{49}Ti NMR parameters of the optimized framework model **A** are depicted (*violet*).

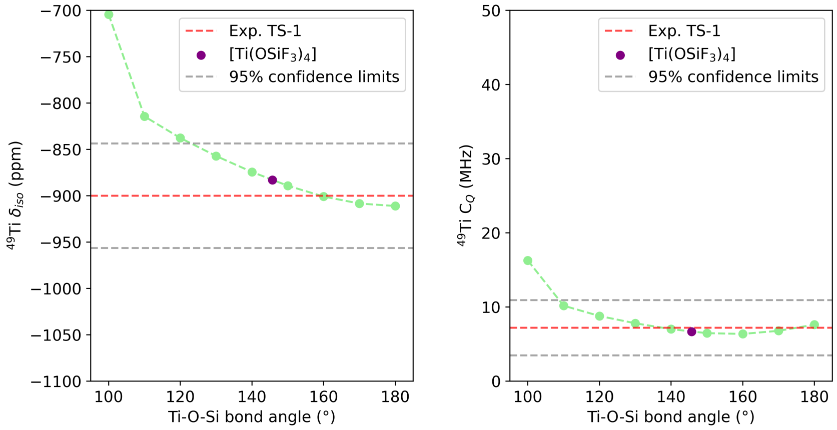

Figure S32: The dependency of the ^{49}Ti NMR parameters δ_{iso} and C_Q of the Ti(OSiF$_3$)$_4$ cluster model on the Ti-O-Si bond angle. As reference points the ^{49}Ti NMR parameters of the optimized framework model **A** are depicted (*violet*).

I.4 ^{17}O NMR Parameter Calculations

The optimized structures employed in the NMR parameter calculation were obtained as described in Section I.2. The ^{17}O NMR parameter calculations[45–47] employed an all-electron spin-orbit relativistic ZORA[38–40] approach using the hybrid PBE0[57]functional and TZ2P[34] basis sets on all elements, in the Amsterdam Modeling Suite (AMS)[35] ADF[50] (version 2023.101) program suite. The numerical quality was set to very good and symmetry was set to NOSYM.

I.4.1 Benchmark and Molecular Model Systems

I.4.1.1 ^{17}O Chemical Shift

The calculated ^{17}O NMR shielding values, obtained via the protocol described in Section I.4, of the benchmark structures yielded very good agreement (linear relationship) with the experimental ^{17}O chemical shift values, similar to what has been previously reported.[58] The benchmark set (**XIII***-**XXII***) is shown below. In general the ^{17}O chemical shift of the computed structures i were calculated via the correlation obtained from the benchmark set (Equation I.2).

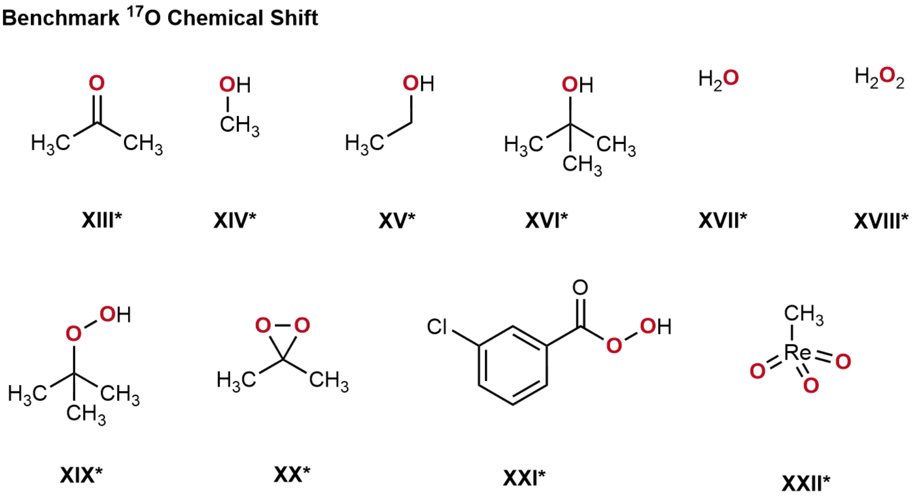

Figure S33: Library of small molecules with perviously reported solution ^{17}O NMR chemical shifts.

$$\delta_{\mathrm{calc},i}(^{17}\mathrm{O}) = 270.5 - 0.908 \times \sigma_{\mathrm{calc},i}(^{17}\mathrm{O}) \qquad \text{(I.2)}$$

Table S30: Summary of computed and experimental ^{17}O NMR parameters of the chemical shift benchmark set. Calculated shielding $\sigma_{\mathrm{calc}}(^{17}\mathrm{O})$ obtained via the protocol described in Section I.4, calculated chemical shift $\delta_{\mathrm{calc}}(^{17}\mathrm{O})$, obtained via the correlation plotted in Figure S34, and the experimental ^{17}O NMR chemical shift $\delta_{\mathrm{exp}}(^{17}\mathrm{O})$ taken from literature.[58–62]

	Formula	$\sigma_{\mathrm{calc}}(^{17}\mathrm{O})$ (ppm)	$\delta_{\mathrm{calc}}(^{17}\mathrm{O})$ (ppm)	$\delta_{\mathrm{exp}}(^{17}\mathrm{O})$ (ppm)
**XIII***	$(CH_3)_2CO$	-357	594	625[58]
**XIV***	CH_3OH	322	-21	-37[59]
**XV***	CH_3CH_2OH	290	8	6[59]
**XVI***	(tBu)OH	225	67	62[59]
**XVII***	H_2O	324	-23	0
**XVIII***	H_2O_2	83	196	195[58]
**XIX***	(tBu)OOH (O)	-22	291	246[60]
**XIX***	(tBu)OOH (OH)	72	206	206[60]
**XX***	DMDO	-38	305	302[61]
**XXI***	mCPBA (O)	-18	287	320[62]
**XXI***	mCPBA (OH)	-7	277	275[62]
**XXII***	MTO	-622	835	820[58]

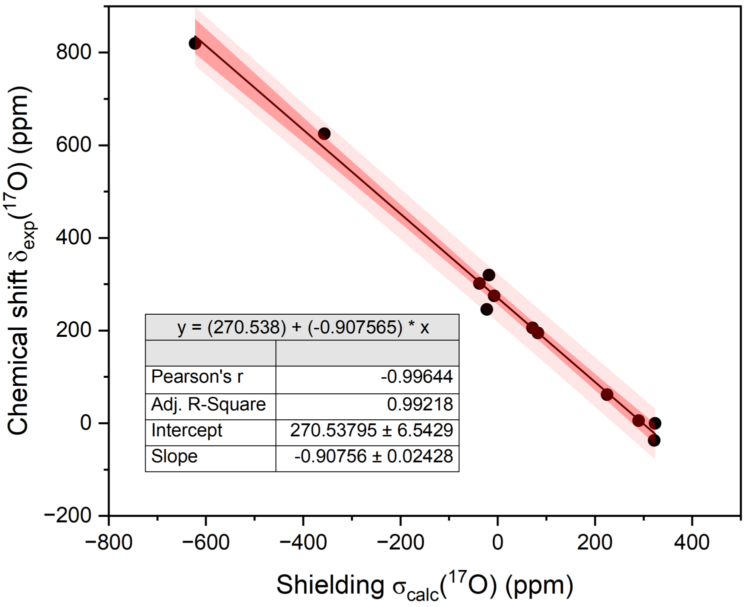

Figure S34: Correlation between experimental chemical shift $\delta_{\text{exp}}(^{17}\text{O})$ values taken from literature and the respective calculated shielding $\sigma_{\text{calc}}(^{17}\text{O})$.

I.4.1.2 ^{17}O Span

Assessment of the ^{17}O span (Ω_{calc}) calculations, computed values were compared with the experimental values (Ω_{exp}), reported in literature[58] (**XIII***, **XVIII*** and **XXII***). Overall, the calculations showed very good agreement with the experimental values (see Table S31).

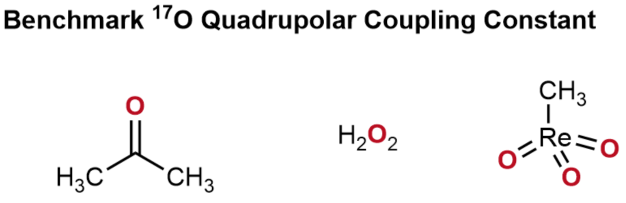

XIII* **XVIII*** **XXII***

Figure S35: Library of small molecules with perviously reported ^{17}O spans.

Table S31: Comparison between the calculated ^{17}O span obtained via the protocol described in Section I.4 and experimental span reported in literature.[58]

	Formula	Ω_{calc} (MHz)	Ω_{exp} (MHz)
**XIII***	$(CH_3)_2CO$	1128	1200[58]
**XVIII***	H_2O_2	443	370[58]
**XXII***	MTO	780	820[58]

I.4.1.3 ^{17}O Quadrupole Coupling Constant

For the assessment of the ^{17}O quadrupole coupling constant ($C_{Q,\ calc}$) calculations, computed values were compared with the experimental values ($C_{Q,\ exp}$), reported in literature[58] (**XIII***, **XVIII*** and **XXII***). Overall, the calculations showed very good agreement with the experimental values (see Table S32).

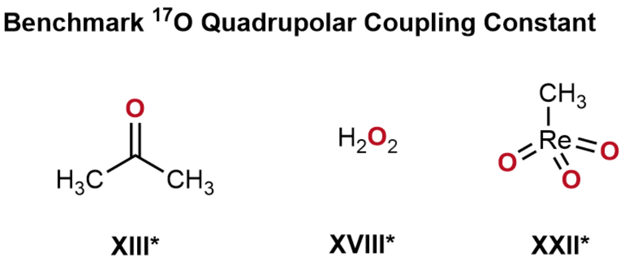

Figure S36: Library of small molecules with perviously reported ^{17}O quadrupole coupling constants.

Table S32: Calculated ^{17}O quadrupole coupling constants via the protocol described in Section I.4 and experimental quadrupole coupling constants reported in literature.[58]

	Formula	$C_{Q,\ calc}$ (MHz)	$C_{Q,\ exp}$ (MHz)
**XIII***	$(CH_3)_2CO$	12.0	12.0[58]
**XVIII***	H_2O_2	17.1	16.0[58]
**XXII***	MTO	4.5	4.0[58]

I.4.2 ^{17}O NMR Signatures of Ti-Zeotypes

In order to further validate the computational approach, the Si-O-Si ^{17}O NMR signature of the parent Silicalite-1 structure was calculated based a similar cluster model ($Si(OSiF_3)_4$) as for the Ti T-sites. The ^{17}O NMR signatures of the framework Ti site in TS-1 were calculated from the $Ti(OSiF_3)_4$ cluster models obtained as described in Section I.2.2. For dinuclear structure a larger clusters was cut from the periodically optimized lattice ($(F_3SiO)_3TiOTi(OSiF_3)_3$). The structures are displayed in Figure S37 with the oxygen atoms of interest highlighted in *red*. In case more than one oxygen atom of the same family is present the average was taken.

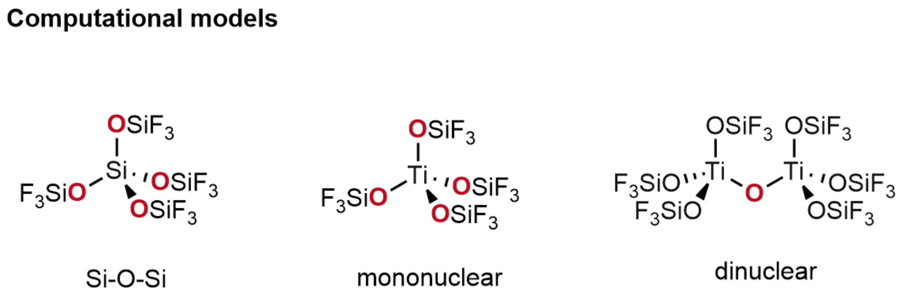

Figure S37: Cluster models of zeolite structures for the calculation of the ^{17}O NMR parameters. In case several equivalent oxygen atoms (marked *red*) are present the average is taken.

I.4.2.1 Silicalite-1 ^{17}O NMR signatures

The experimental ^{17}O NMR signature of Si-O-Si in the Silicalite-1 structure ($\delta_{\text{iso, exp}} = 39$ ppm, $\Omega_{\text{exp}} = 80$ ppm, $\kappa_{\text{exp}} = 0.38$, $C_{\text{Q, exp}} = 5.4$ MHz, $\eta_{\text{exp}} = 0.1$) agrees well with the averaged calculated ^{17}O NMR signature of the four O atoms in the cluster model ($\delta_{\text{iso, calc}} = 48$ ppm, $\Omega_{\text{calc}} = 84$ ppm, $\kappa_{\text{calc}} = 0.9$, $C_{\text{Q, calc}} = 6.1$ MHz, $\eta_{\text{calc}} = 0.25$), confirming that the cluster size is sufficiently large.

I.4.2.2 ^{17}O Sites in TS-1

Besides the ^{17}O NMR signature of Si-O-Si in the Silicalite-1 structure, the calculated ^{17}O NMR parameters of Ti-O-Si and Ti-O-Ti (mononuclear Ti sites (Ti-O-Si) and dinuclear Ti sites (Ti-O-Ti), see Table S33) allow us to obtain an estimate of the expected δ_{iso} and Ω for the Ti-^{17}O-Si and Ti-^{17}O-Ti signatures. Notably, the δ_{iso} increases by ca. 300 ppm for each Ti directly connected to the bridging oxygen, *i.e.* 48, 281 and 640 ppm for (Si-O-Si), (Ti-O-Si) and (Ti-O-Ti), respectively. In parallel, the Ω value increases from 84 ppm (Si-O-Si) to 425 ppm (Ti-O-Si) and 736 ppm (Ti-O-Ti). Based on those values, the most deshielded peak (*violet* in Figure 1) and the peak centered around ca. 40 ppm (*green* in Figure 1) in the ^{17}O NMR spectrum of TS-1 treated with $H_2{}^{17}O$ are confidently assigned to Ti-O-Si and Si-O-Si (Figure 1). Not observing Ti pairs in the pristine catalyst suggests, that the observed dinuclear sites in the catalyst are generated $in - situ$ upon addition of H_2O_2, highlighting the dynamic nature of these catalysts.

Table S33: Summary of calculated ^{17}O NMR signatures of the oxygen atoms for proposed sites in TS-1, namely Si-O-Si, Ti-O-Si and Ti-O-Ti.

Species	δ_{iso}(ppm)	Ω (ppm)	κ	C_Q (MHz)	η
Si-O-Si	48	84	0.9	6.1	0.25
Ti-O-Si (**A**)	281	425	0.77	4.0	0.01
Ti-O-Ti	640	736	0.71	1.00	0.41

I.4.3 ^{17}O NMR Parameters Dependency of $Ti(OSiF_3)_4$ Cluster Model on Ti-O Bond Length, O-Ti-O and Ti-O-Si Bond Angle

The ^{17}O NMR parameters dependency in a tetrahedral environment on structural changes was investigated similar as discussed before for the ^{49}Ti NMR signatures (see Section I.3.3). We therefore started from the optimized framework model **A** ($Ti(OSiF_3)_4$, obtained as described in Section I.2). In a next step, bond length and bond angle perturbations in the framework model **A** structure were introduced, while keeping the other atom positions fixed, as depicted in Figure S38. The ^{17}O NMR parameters were calculated for these structures without further optimization and the resulting $\delta_{\text{iso}}(^{17}\text{O})$ and $\Omega(^{17}\text{O})$ are depicted in Figure S39-S41. In a final step the ^{17}O NMR parameters of all geometrical perturbations were compared to the experimental values of the oxygen species assigned to the Ti-O-Si (*red* line) and their associated 95% confidence limits based on the χ^2_R (*grey* lines, see Section for further details I.5). From this analysis it becomes evident, that even large perturbations lead to rather small effects in the ^{17}O NMR signatures (most calculated models fall within the 95% confidence limits).

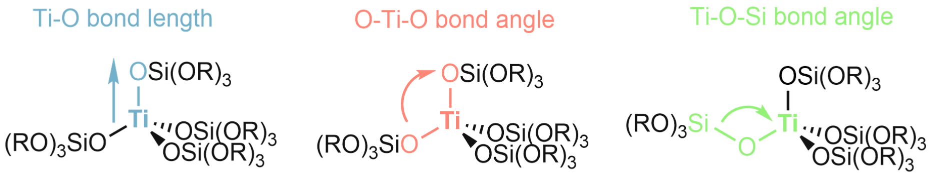

Figure S38: The dependency of the ^{17}O NMR parameters δ_{iso} and Ω of the $Ti(OSiF_3)_4$ cluster model on the Ti-O bond length, O-Ti-O angle and Ti-O-Si angle.

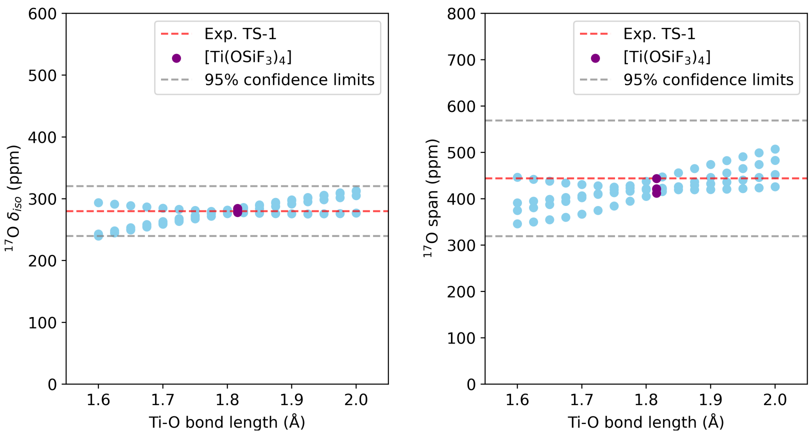

Figure S39: The dependency of the ^{17}O NMR parameters δ_{iso} and Ω of the $Ti(OSiF_3)_4$ cluster model on the Ti-O bond length. As reference points the ^{17}O NMR parameters of the optimized framework model **A** are depicted (*violet*).

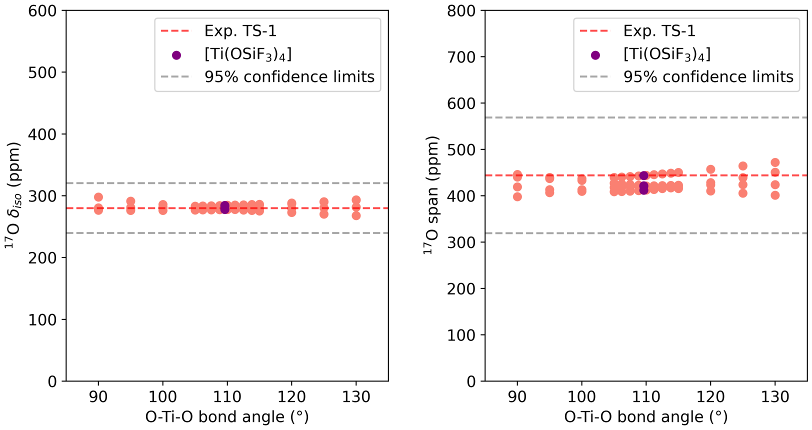

Figure S40: The dependency of the ^{17}O NMR parameters δ_{iso} and Ω of the $Ti(OSiF_3)_4$ cluster model on the O-Ti-O bond angle. As reference points the ^{17}O NMR parameters of the optimized framework model **A** are depicted (*violet*).

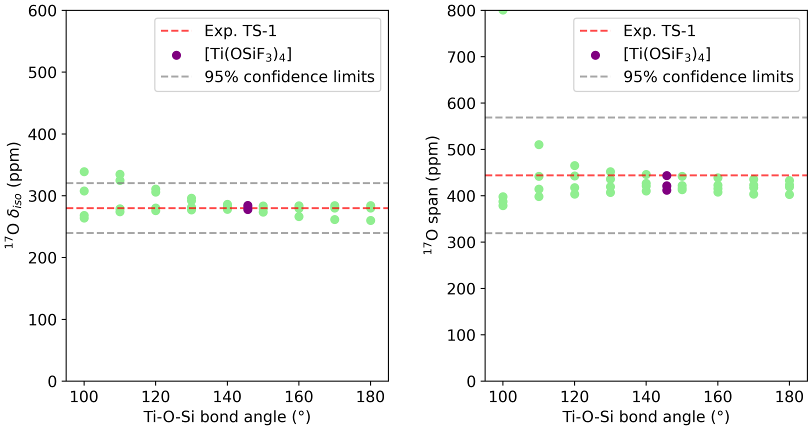

Figure S41: The dependency of the ^{17}O NMR parameters δ_{iso} and Ω of the $Ti(OSiF_3)_4$ cluster model on the Ti-O-Si bond angle. As reference points the ^{17}O NMR parameters of the optimized framework model **A** are depicted (*violet*).

I.5 Statistical Evaluation

I.5.1 Global Uncertainties in the Computational Protocol

The computational protocol including periodic geometry optimization and NMR parameter calculations was evaluated based on our performed benchmark study, which allowed us to extract the root-mean-square error (RMSE) (see Equation I.3) of the respective NMR parameter (δ_{iso} (^{49}Ti), C_Q(^{49}Ti), δ_{iso} (^{17}O), Ω (^{17}O) and C_Q(^{17}O)). The *a priori* determination of the global uncertainties for the predicted NMR parameters allowed for the quantitative comparison of the predicted and experimental NMR parameters as described below.[63,64]

$$\mathrm{RMSE} = \sqrt{\frac{\sum_{i=1}^{n}(x_{i,\mathrm{calc}} - x_{i,\mathrm{exp}})^2}{n}} \tag{I.3}$$

where $x_{i,\mathrm{calc}}$ are the calculated δ_{iso}, Ω or C_Q values and $x_{i,\mathrm{exp}}$ the corresponding experimental values.

For the RMSE calculations of the $^{47/49}$Ti NMR parameters, the experimental and calculated values of the molecular library of Ti complexes (closely related to the studied Ti-sites in Ti-zeotypes) was employed (see Tables S26 and S27). For the ^{17}O NMR parameters, the respective ^{17}O NMR chemical shift, Ω and C_Q benchmark sets were used (see Tables S30, S31 and S32).

Table S34: Summary of the RMSE values for the NMR parameters: δ_{iso} (^{49}Ti), C_Q(^{49}Ti), δ_{iso} (^{17}O), Ω (^{17}O) and C_Q(^{17}O).

	RMSE
δ_{iso} (^{49}Ti)	28.8 ppm
C_Q(^{49}Ti)	1.9 MHz
δ_{iso} (^{17}O)	20.6 ppm
Ω (^{17}O)	63.6 ppm
C_Q(^{17}O)	0.7 MHz

I.5.2 χ^2_R Statistic

The structural models of Ti sites in classical TS-1 with minimal amount of TiO_2 were ranked using a reduced chi-squared (χ^2_R) statistic.[65] This gives a quantitative measure of the agreement of the NMR parameters obtained via the given described NMR calculation protocol and the experimental NMR values,[63,64]

$$\chi^2_R = \frac{1}{N-f}\sum_{i}^{N}\frac{(x_i^{\mathrm{model}} - x_i^{\mathrm{exp}})^2}{s_i^2} \tag{I.4}$$

where x_i^{model} are the predicted δ_{iso} or C_Q values of the different structural models and x_i^{exp} the corresponding experimental values, N are all NMR parameters considered (we have focused on the ^{49}Ti NMR parameters, which were shown to be sensitive to the Ti speciation and Ti site distortions. Namely, the isotropic chemical shift δ_{iso} (^{49}Ti) and quadrupole coupling constant describing the quadrupole interaction of the the dominant local Ti environment ($C_{Q,0}$, see Extended Czjzek Simulations Section H.5) were used as the experimental values), f the number of adjustable model parameters, which in the case of direct model ranking is 0 and s_i^2 is the nuclide-specific weighting derived by setting s_i to the corresponding root-mean-square error derived from benchmark studies described in Section I.5.1. We further make the assumption that the residuals between theory and experiment are normally distributed, and therefore the χ^2_R statistic follows a reduced chi-squared distribution. We note however, that the size of the benchmark set is limited, due to the number of accessible reference molecules.

Table S35: Summary of χ_R^2 values based on δ_{iso} (^{49}Ti) and C_Q(^{49}Ti) of framework and framework associated sites for TS-$1_{1.5}$.

Species	TS-$1_{1.5}$
**A**	0.2
**B**	8.5
**C**	34.2
**D**	133.2
**E**	82.3
**F**	173.8

Table S36: Summary of χ_R^2 values based on δ_{iso} (^{49}Ti) and C_Q(^{49}Ti) of the T-sites of classical TS-1s with minimal amount of TiO_2.

T-site	TS-1_1	TS-$1_{1.5}$	TS-$1_{1.9}$
**T1**	0.8	0.5	0.4
**T2**	2.9	3.0	3.0
**T3**	1.5	1.3	1.3
**T4**	1.5	1.2	1.4
**T5**	3.0	3.0	3.4
**T6**	2.6	3.8	3.3
**T7**	2.6	2.6	2.3
**T8**	0.3	0.2	0.2
**T9**	3.9	3.2	3.3
**T10**	4.3	4.4	4.3
**T11**	3.9	3.6	3.2
**T12**	2.2	1.6	2.1

References

(1) Gordon, C. P.; Engler, H.; Tragl, A. S.; Plodinec, M.; Lunkenbein, T.; Berkessel, A.; Teles, J. H.; Parvulescu, A. N.; Copéret, C. Efficient epoxidation over dinuclear sites in titanium silicalite-1. *Nature* **2020**, *586*, DOI: 10.1038/s41586-020-2826-3.

(2) Lätsch, L.; Kaul, C. J.; Yakimov, A. V.; McEntee, R.; Baerdemaeker, T. D.; Parvulescu, A.-N.; Seidel, K.; Teles, J. H.; Copéret, C. Nature of Reactive Sites in TS-1 from 15N Solid-State NMR and Ti K-Edge X-Ray Absorption Spectroscopic Signatures Upon Pyridine Adsorption. *Journal of the American Chemical Society* **2024**, *146*, 29675–29683, DOI: 10.1021/jacs.4c10604, https://doi.org/10.1021/jacs.4c10604.

(3) McMullen, A. K.; Tilley, T. D.; Rheingold, A. L.; Geib, S. J. Preparation and Characterization of the Monomeric Copper(II) Siloxide Complex Cu[OSi(OCMe3)3]2(py)2. *Inorganic Chemistry* **1989**, *28*, DOI: 10.1021/ic00318a032.

(4) Manxzer, L. E.; Deaton, J.; Sharp, P.; Schrock, R. R. In 1982, DOI: 10.1002/9780470132524.ch31.

(5) Lätsch, L.; Kaul, C. J.; Yakimov, A. V.; Müller, I. B.; Hassan, A.; Perrone, B.; Aghazada, S.; Berkson, Z. J.; Baerdemaeker, T. D.; Parvulescu, A. N.; Seidel, K.; Teles, J. H.; Copéret, C. NMR Signatures and Electronic Structure of Ti Sites in Titanosilicalite-1 from Solid-State 47/49Ti NMR Spectroscopy. *Journal of the American Chemical Society* **2023**, *145*, DOI: 10.1021/jacs.2c09867.

(6) Takahiro, G.; Takayuki, K.; Yoshimoto, A. Crystallization Behavior of SiO2-TiO2 Ceramics Derived from Titanosiloxanes on Pyrolysis. *Journal of Sol-Gel Science and Technology* **1998**, *13*.

(7) Pérez-Pérez, J.; Gallardo-Garibay, A.; Martínez-Otero, D.; Hernández-Balderas, U.; Jancik, V. Formation of Titanosilicate N,N-dialkyl Carbamates by CO2 Insertion into the [(tBuO)3SiO]3TiNR2 Scaffold. *European Journal of Inorganic Chemistry* **2024**, *27*, e202400337, DOI: https://doi.org/10.1002/ejic.202400337, https://doi.org/10.1002/ejic.202400337.

(8) Lätsch, L.; Guda, S. A.; Romankov, V.; Wartmann, C.; Neudörfl, J. M.; Dreiser, J.; Berkessel, A.; Guda, A. A.; Copéret, C. Tracking Coordination Environment and Reaction Intermediates in Homogeneous and Heterogeneous Epoxidation Catalysts via Ti L2,3-Edge Near-Edge X-ray Absorption Fine Structures. *Journal of the American Chemical Society* **2024**, *146*, DOI: 10.1021/jacs.3c12831.

(9) Dolomanov, O. V.; Bourhis, L. J.; Gildea, R. J.; Howard, J. A.; Puschmann, H. OLEX2: A complete structure solution, refinement and analysis program. *Journal of Applied Crystallography* **2009**, *42*, DOI: 10.1107/S0021889808042726.

(10) Sheldrick, G. M. SHELXT - Integrated space-group and crystal-structure determination. *Acta Crystallographica Section A: Foundations of Crystallography* **2015**, *71*, DOI: 10.1107/S2053273314026370.

(11) Sheldrick, G. M. Crystal structure refinement with SHELXL. *Acta Crystallographica Section C: Structural Chemistry* **2015**, *71*, DOI: 10.1107/S2053229614024218.

(12) Noh, G.; Lam, E.; Alfke, J. L.; Larmier, K.; Searles, K.; Wolf, P.; Copéret, C. Selective Hydrogenation of CO2 to CH3OH on Supported Cu Nanoparticles Promoted by Isolated Ti IV Surface Sites on SiO2. *ChemSusChem* **2019**, *12*, DOI: 10.1002/cssc.201900134.

(13) Pathak, M.; Bohra, R.; Mehrotra, R. C.; Lorenz, I. P.; Piotrowski, H. Synthetic studies and structural aspects of novel metallacyclic compounds of titanium(IV) incorporating nitrogen, oxygen and sulphur: 1. Reactions of cis-dialkoxy-bis(acetylacetonato)titanium(IV) with alkoxyalkanols and the crystal structure of a new modification: di--oxo-bis[diacetylacetonatotitanium(IV)]. *Transition Metal Chemistry* **2003**, *28*, DOI: 10.1023/A:1022901918955.

(14) Ravel, B.; Newville, M. In *Journal of Synchrotron Radiation*, 2005; Vol. 12, DOI: 10.1107/S0909049505012719.

(15) Yakimov, A.; Guda, A.; Guda, S.; Protsenko, B.; Groppo, E.; Bolner, F. M.; Norsic, S.; Raynaud, J.; Monteil, V.; Copéret, C. Nature of Surface Sites in Ziegler-Natta Pre-Catalysts from Quantitative Analysis of Ti K-edge X-Ray Absorption Spectra. *ChemRxiv., This content is a preprint and has not been peer-reviewed.* **2025**, DOI: 10.26434/chemrxiv-2025-2dv1x.

(16) Toby, B. H.; Dreele, R. B. V. GSAS-II: The genesis of a modern open-source all purpose crystallography software package. *Journal of Applied Crystallography* **2013**, *46*, DOI: 10.1107/S0021889813003531.

(17) Artioli, G.; Lamberti, C.; Marra, G. L. Neutron powder diffraction study of orthorhombic and monoclinic defective silicalite. *Acta Crystallographica Section B: Structural Science* **2000**, *56*, DOI: 10.1107/S0108768199008927.

(18) Zagorac, D.; Muller, H.; Ruehl, S.; Zagorac, J.; Rehme, S. Recent developments in the Inorganic Crystal Structure Database: Theoretical crystal structure data and related features. *Journal of Applied Crystallography* **2019**, *52*, DOI: 10.1107/S160057671900997X.

(19) Cline, J. P.; Deslattes, R. D.; J L, S.; E G, K.; Hudson, L. T.; Henins, A *The certification of SRM 640c; the primary NIST line position SRM for powder diffraction*; tech. rep.; Australian X-ray Analytical Association, Melbourne , VIC (Australia), 1999.

(20) Massiot, D.; Fayon, F.; Capron, M.; King, I.; Calvé, S. L.; Alonso, B.; Durand, J. O.; Bujoli, B.; Gan, Z.; Hoatson, G. Modelling one- and two-dimensional solid-state NMR spectra. *Magnetic Resonance in Chemistry* **2002**, *40*, DOI: 10.1002/mrc.984.

(21) Rossini, A. J.; Hung, I.; Schurko, R. W. Solid-state 47/49Ti NMR of titanocene chlorides. *Journal of Physical Chemistry Letters* **2010**, *1*, DOI: 10.1021/jz1012017.

(22) Hung, I.; Gor'kov, P.; Gan, Z. Using the heteronuclear Bloch-Siegert shift of protons for B1 calibration of insensitive nuclei not present in the sample. *Journal of Magnetic Resonance* **2020**, *310*, DOI: 10.1016/j.jmr.2019.106636.

(23) Carr, H. Y.; Purcell, E. M. Effects of diffusion on free precession in nuclear magnetic resonance experiments. *Physical Review* **1954**, *94*, DOI: 10.1103/PhysRev.94.630.

(24) Meiboom, S.; Gill, D. Modified spin-echo method for measuring nuclear relaxation times. *Review of Scientific Instruments* **1958**, *29*, DOI: 10.1063/1.1716296.

(25) Kentgens, A. P.; Verhagen, R. Advantages of double frequency sweeps in static, MAS and MQMAS NMR of spin I=3/2 nuclei. *Chemical Physics Letters* **1999**, *300*, DOI: 10.1016/S0009-2614(98)01402-X.

(26) Iuga, D.; Schäfer, H.; Verhagen, R.; Kentgens, A. P. Population and Coherence Transfer Induced by Double Frequency Sweeps in Half-Integer Quadrupolar Spin Systems. *Journal of Magnetic Resonance* **2000**, *147*, DOI: 10.1006/jmre.2000.2192.

(27) Ēriks Kupče; Freeman, R. Adiabatic Pulses for Wideband Inversion and Broadband Decoupling. *Journal of Magnetic Resonance, Series A* **1995**, *115*, DOI: 10.1006/jmra.1995.1179.

(28) Caër, G. L.; Bureau, B.; Massiot, D. An extension of the Czjzek model for the distributions of electric field gradients in disordered solids and an application to NMR spectra of 71Ga in chalcogenide glasses. *Journal of Physics Condensed Matter* **2010**, *22*, DOI: 10.1088/0953-8984/22/6/065402.

(29) Vasconcelos, F.; Cristol, S.; Paul, J. F.; Delevoye, L.; Mauri, F.; Charpentier, T.; Caër, G. L. Extended Czjzek model applied to NMR parameter distributions in sodium metaphosphate glass. *Journal of Physics Condensed Matter* **2013**, *25*, DOI: 10.1088/0953-8984/25/25/255402.

(30) Bak, M.; Rasmussen, J. T.; Nielsen, N. C. SIMPSON: A General Simulation Program for Solid-State NMR Spectroscopy. *Journal of Magnetic Resonance* **2000**, *147*, DOI: 10.1006/jmre.2000.2179.

(31) Kadantsev, E. S.; Klooster, R.; Boeij, P. L. D.; Ziegler, T. The formulation and implementation of analytic energy gradients for periodic density functional calculations with STO/NAO Bloch basis set. *Molecular Physics* **2007**, *105*, DOI: 10.1080/00268970701598063.

(32) Becke, A. D. Density-functional exchange-energy approximation with correct asymptotic behavior. *Physical Review A* **1988**, *38*, DOI: 10.1103/PhysRevA.38.3098.

(33) Perdew, J. P. Density-functional approximation for the correlation energy of the inhomogeneous electron gas. *Physical Review B* **1986**, *33*, DOI: 10.1103/PhysRevB.33.8822.

(34) Lenthe, E. V.; Baerends, E. J. Optimized Slater-type basis sets for the elements 1-118. *Journal of Computational Chemistry* **2003**, *24*, DOI: 10.1002/jcc.10255.

(35) AMS, 2023, http://www.scm.com.

(36) Velde, G. T.; Baerends, E. J. Precise density-functional method for periodic structures. *Physical Review B* **1991**, *44*, DOI: 10.1103/PhysRevB.44.7888.

(37) BAND, 2023, http://www.scm.com.

(38) Lenthe, E. V.; Baerends, E. J.; Snijders, J. G. Relativistic regular two-component Hamiltonians. *The Journal of Chemical Physics* **1993**, *99*, DOI: 10.1063/1.466059.

(39) Lenthe, E. V.; Baerends, E. J.; Snijders, J. G. Relativistic total energy using regular approximations. *The Journal of Chemical Physics* **1994**, *101*, DOI: 10.1063/1.467943.

(40) Lenthe, E. V. Geometry optimizations in the zero order regular approximation for relativistic effects. *Journal of Chemical Physics* **1999**, *110*, DOI: 10.1063/1.478813.

(41) Nieger, M.; Hupfer, H. CCDC 113637: Experimental Crystal Structure Determination. **1999**, DOI: 10.5517/cc3t7qv..

(42) McKenzie, T. C.; Sanner, R. D.; Bercaw, J. E. The crystal and molecular structure of bis(pentamethylcyclopentadienyl)dichlorotitanium(IV). *Journal of Organometallic Chemistry* **1975**, *102*, DOI: 10.1016/S0022-328X(00)89390-0.

(43) Pevec, A. Crystal structure of (5C5Me5)TiCl3 and (5C5Me4H)TiCl3. *Acta Chim. Slov.* **2003**, *50*, 199–206.

(44) Rannabauer, S.; Schnick, W. Synthese, Kristallstruktur und spektroskopische Charakterisierung von Bis-(dimethylammonium)hexachlorotitanat [Me2NH2]2[TiCl6]/ Synthesis, Crystal Structure, and Spectroscopic Characterization of Bis-(dimethylammonium) Hexachlorotitanate [Me2NH2]2[TiCl6]. *Zeitschrift für Naturforschung B* **2003**, *58*, DOI: 10.1515/znb-2003-0509.

(45) Schreckenbach, G.; Ziegler, T. Calculation of NMR shielding tensors using gauge-including atomic orbitals and modern density functional theory. *Journal of Physical Chemistry* **1995**, *99*, DOI: 10.1021/j100002a024.

(46) Krykunov, M.; Ziegler, T.; Lenthe, E. V. Hybrid density functional calculations of nuclear magnetic shieldings using slater-type orbitals and the zeroth- order regular approximation. *International Journal of Quantum Chemistry* **2009**, *109*, DOI: 10.1002/qua.21985.

(47) Lenthe, E. V.; Baerends, E. J. Density functional calculations of nuclear quadrupole coupling constants in the zero-order regular approximation for relativistic effects. *Journal of Chemical Physics* **2000**, *112*, DOI: 10.1063/1.481433.

(48) Becke, A. D. Density-functional thermochemistry. III. The role of exact exchange. *The Journal of Chemical Physics* **1993**, *98*, DOI: 10.1063/1.464913.

(49) Stephens, P. J.; Devlin, F. J.; Chabalowski, C. F.; Frisch, M. J. Ab Initio calculation of vibrational absorption and circular dichroism spectra using density functional force fields. *Journal of Physical Chemistry* **1994**, *98*, DOI: 10.1021/j100096a001.

(50) ADF, 2023, http://www.scm.com.

(51) Bühl, M.; Mauschick, F. T. Density functional computation of 49Ti NMR chemical shifts. *Magnetic Resonance in Chemistry* **2004**, *42*, DOI: 10.1002/mrc.1405.

(52) Berger, S.; Bock, W.; Frenking, G.; Jonas, V.; Müller, F. NMR Data of Methyltitanium Trichloride and Related Organometallic Compounds. A Combined Experimental and Theoretical Study of MenXCl4-n(n = 0-4; X = C, Si, Sn, Pb, Ti). *Journal of the American Chemical Society* **1995**, *117*, DOI: 10.1021/ja00118a018.

(53) Koch, R.; Bruhn, T. Theoretical 49Ti NMR chemical shifts. *Journal of Molecular Modeling* **2006**, *12*, DOI: 10.1007/s00894-005-0081-z.

(54) Yakimov, A. V.; Kaul, C. J.; Kakiuchi, Y.; Sabisch, S.; Bolner, F. M.; Raynaud, J.; Monteil, V.; Berruyer, P.; Copéret, C. Well-Defined Ti Surface Sites in Ziegler-Natta Pre-Catalysts from 47/49Ti Solid-State Nuclear Magnetic Resonance Spectroscopy. *Journal of Physical Chemistry Letters* **2024**, *15*, DOI: 10.1021/acs.jpclett.3c03119.

(55) Hao, N.; Sayer, B. G.; Dénès, G.; Bickley, D. G.; Detellier, C.; McGlinchey, M. J. Titanium-47 and -49 nuclear magnetic resonance spectroscopy: Chemical applications. *Journal of Magnetic Resonance (1969)* **1982**, *50*, DOI: 10.1016/0022-2364(82)90030-0.

(56) Berger, S.; Bock, W.; Marth, C. F.; Raguse, B.; Reetz, M. T. 47/49Ti NMR of some titanium compounds. *Magnetic Resonance in Chemistry* **1990**, *28*, DOI: 10.1002/mrc.1260280617.

(57) Adamo, C.; Barone, V. Toward reliable density functional methods without adjustable parameters: The PBE0 model. *Journal of Chemical Physics* **1999**, *110*, DOI: 10.1063/1.478522.

(58) Ehinger, C.; Gordon, C. P.; Copéret, C. Oxygen transfer in electrophilic epoxidation probed by 17O NMR: Differentiating between oxidants and role of spectator metal oxo. *Chemical Science* **2019**, *10*, DOI: 10.1039/c8sc04868a.

(59) Crandall, J. K.; Centeno, M. A. Oxygen-17 Nuclear Magnetic Resonance. 1. Alcohols. *Journal of Organic Chemistry* **1979**, *44*, DOI: 10.1021/jo01321a042.

(60) Barieux, J. J.; Schirmann, J. P. 17O-enriched hydrogen peroxide and t.butyl hydroperoxide: Synthesis, characterization and some applications. *Tetrahedron Letters* **1987**, *28*, DOI: 10.1016/S0040-4039(00)96883-7.

(61) Cassidei, L.; Florentino, M.; Mello, R.; Sciacovelli, O.; Curci, R. Oxygen-17 and Carbon-13 Identification of the Dimethyldioxirane Intermediate Arising in the Reaction of Potassium Caroate with Acetone, 1987, DOI: 10.1021/jo00380a045.

(62) Antolini, L.; Benassi, R.; Ghelli, S.; Folli, U.; Sbardellati, S.; Taddei, F. The influence of steric constraints on the conformational properties and on the 17O NMR shielding of ortho-substituted perbenzoates. *Journal of the Chemical Society, Perkin Transactions 2* **1992**, DOI: 10.1039/p29920001907.

(63) Caulkins, B. G.; Young, R. P.; Kudla, R. A.; Yang, C.; Bittbauer, T. J.; Bastin, B.; Hilario, E.; Fan, L.; Marsella, M. J.; Dunn, M. F.; Mueller, L. J. NMR Crystallography of a Carbanionic Intermediate in Tryptophan Synthase: Chemical Structure, Tautomerization, and Reaction Specificity. *Journal of the American Chemical Society* **2016**, *138*, DOI: 10.1021/jacs.6b08937.

(64) Holmes, J. B.; Liu, V.; Caulkins, B. G.; Hilario, E.; Ghosh, R. K.; Drago, V. N.; Young, R. P.; Romero, J. A.; Gill, A. D.; Bogie, P. M.; Paulino, J.; Wang, X.; Riviere, G.; Bosken, Y. K.; Struppe, J.; Hassan, A.; Guidoulianov, J.; Perrone, B.; Mentink-Vigier, F.; Chang, C. E. A.; Long, J. R.; Hooley, R. J.; Mueser, T. C.; Dunn, M. F.; Mueller, L. J. Imaging active site chemistry and protonation states: NMR crystallography of the tryptophan synthase -aminoacrylate intermediate. *Proceedings of the National Academy of Sciences of the United States of America* **2022**, *119*, DOI: 10.1073/pnas.2109235119.

(65) Garland, C. W.; Shoemaker, D. P.; Nibler, J. W., *Experiments in Physical Chemistry*, 5th; McGraw-Hill: 1989, p 890.
